# Supplementary material for: The Core and Distinction of the Gut Microbiota in Chinese Populations across Geography and Ethnicity
Source: Microorganisms. 2020 Oct 14;8(10):1579. doi: 10.3390/microorganisms8101579 (PMC7602275; doi:10.3390/microorganisms8101579)
Supplement: Supplementary file 1 [file microorganisms-08-01579-s001.zip › Supplement/Figure S1 Venn diagram.pdf]

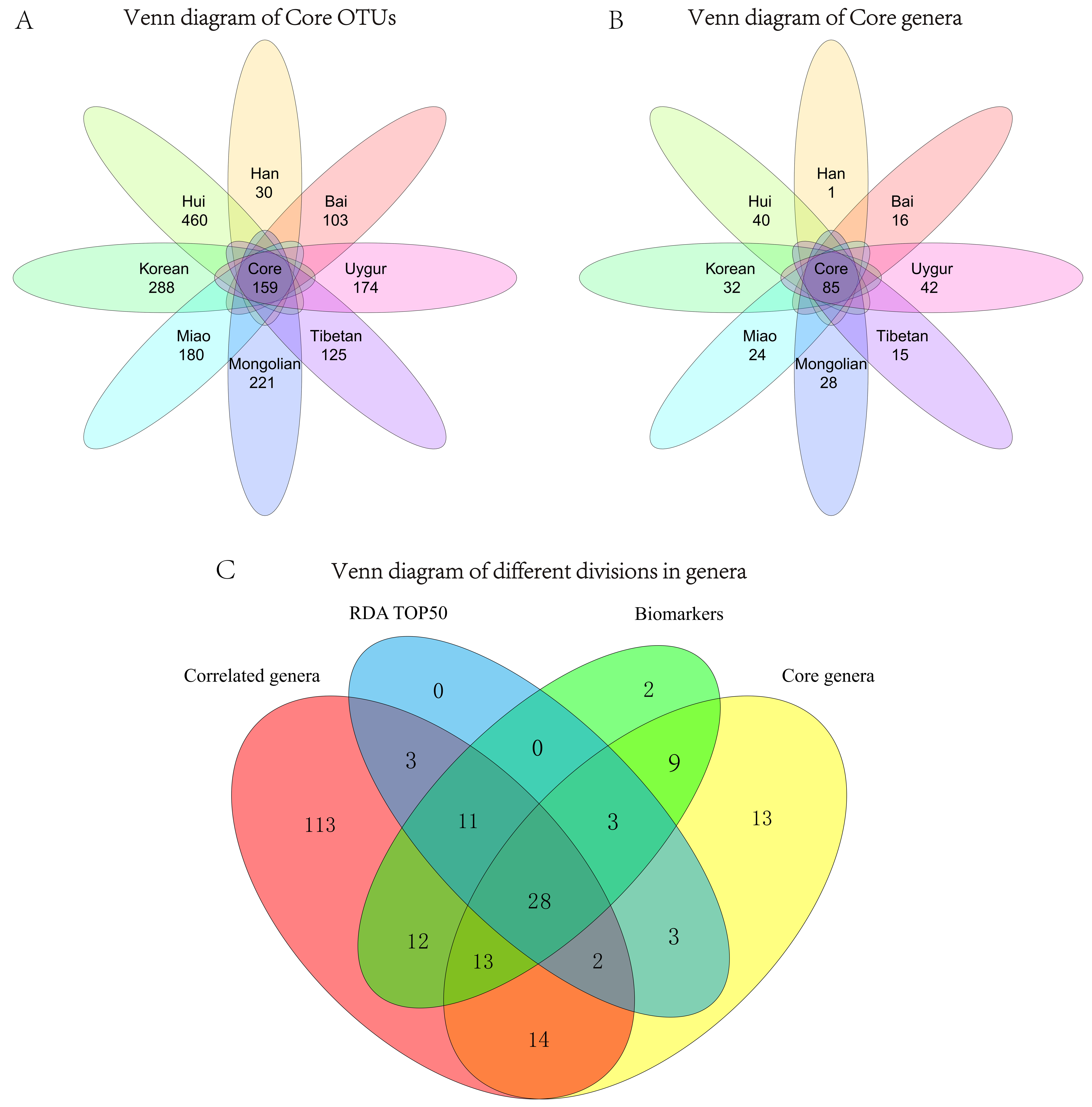
A
Venn diagram of Core OTUs
Han
30
Hui
460
Bai
103
Korean
288
Core
159
Uygur
174
Miao
180
Tibetan
125
Mongolian
221
B
Venn diagram of Core genera
Han
1
Hui
40
Bai
16
Korean
32
Core
85
Uygur
42
Miao
24
Tibetan
15
Mongolian
28
C
Venn diagram of different divisions in genera
RDA TOP50
Biomarkers
Correlated genera
Core genera
0
2
3
0
9
113
11
3
13
28
12
3
13
2
14
