# Supplementary material for: The Core and Distinction of the Gut Microbiota in Chinese Populations across Geography and Ethnicity
Source: Microorganisms. 2020 Oct 14;8(10):1579. doi: 10.3390/microorganisms8101579 (PMC7602275; doi:10.3390/microorganisms8101579)
Supplement: Supplementary file 1 [file microorganisms-08-01579-s001.zip › Supplement/Figure S2 Results of Variation decomposition.pdf]

Fig.S2 Combined explanation and exclusive explanation of all bacterium at each taxonomic level and KOs between geographic location and ethnicity by Variation decomposition;“Combined explanation”, “Geography exclusive explanation” and “Ethnicity exclusive explanation” was respectively expressed by loess lines (span = 0.5) in black, blue and red; The X-axis represents rank-ordered of the average of bacterium or KOs proportion; The Y-axis represents Adjusted R square.

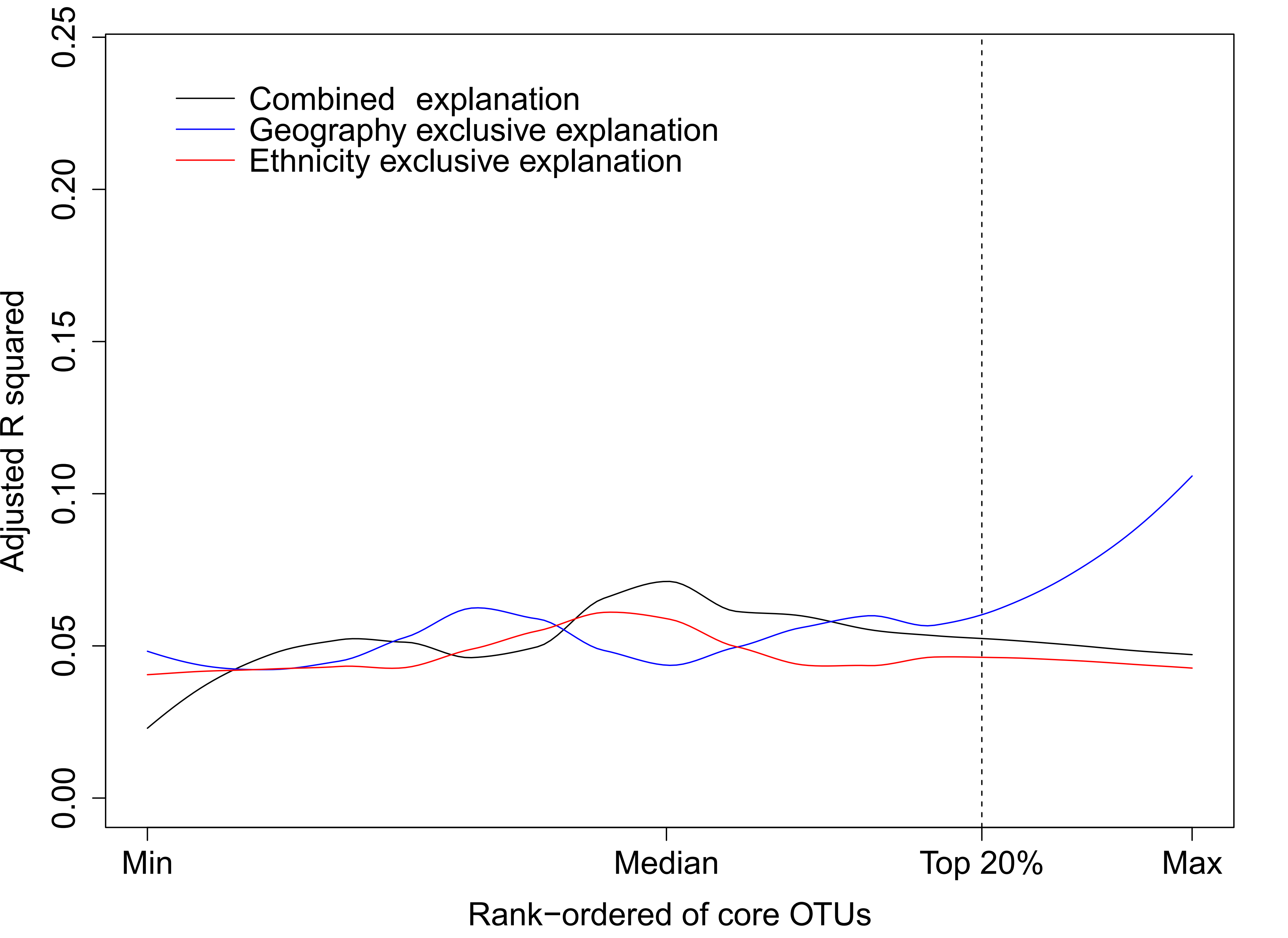

Combined explanation
Geography exclusive explanation
Ethnicity exclusive explanation
Adjusted R squared
0.00
0.05
0.10
0.15
0.20
0.25
Min
Median
Top 20%
Max
Rank−ordered of core OTUs

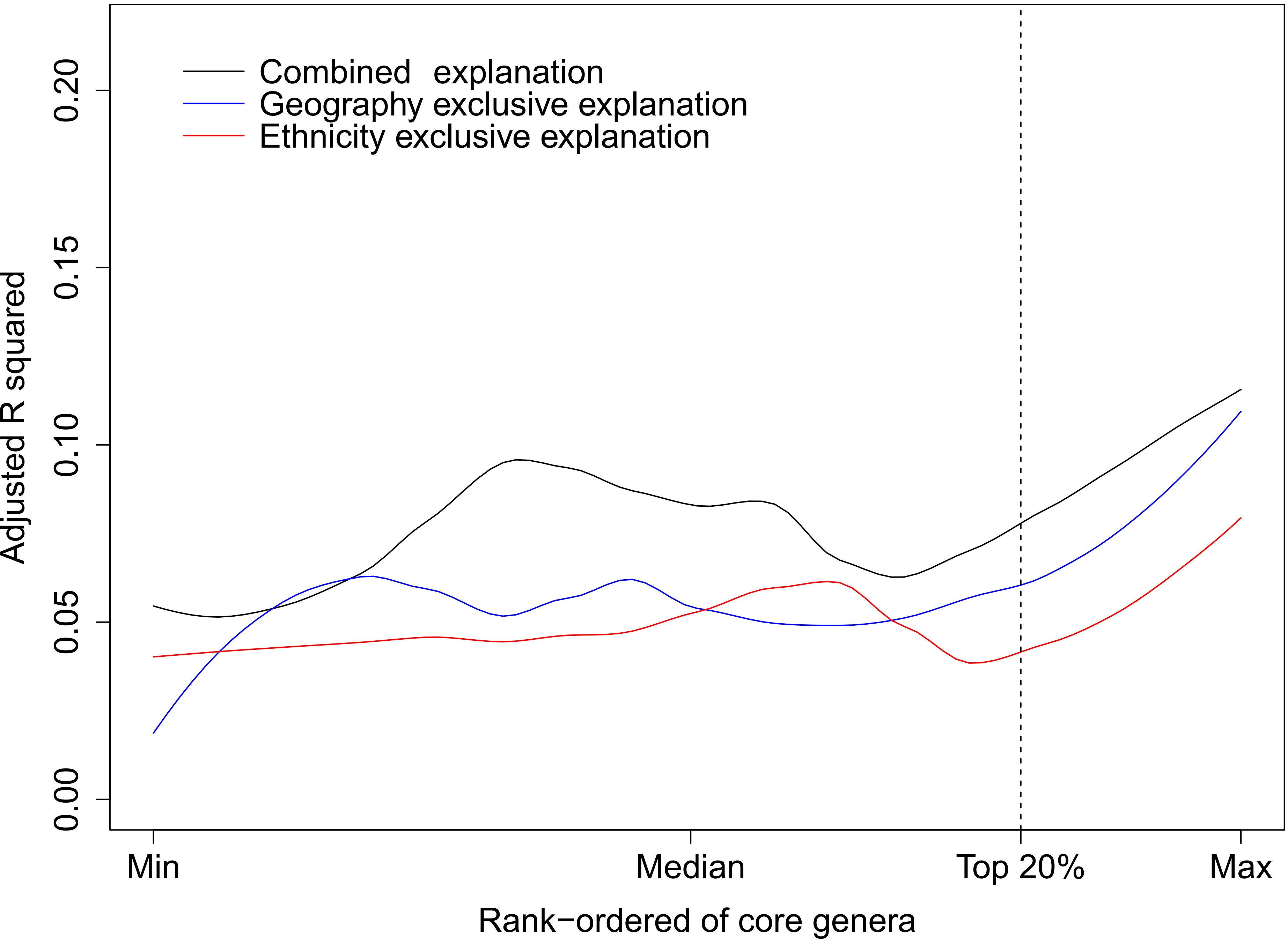
Combined explanation
Geography exclusive explanation
Ethnicity exclusive explanation
Adjusted R squared
0.00
0.05
0.10
0.15
0.20
Min
Median
Top 20%
Max
Rank−ordered of core genera

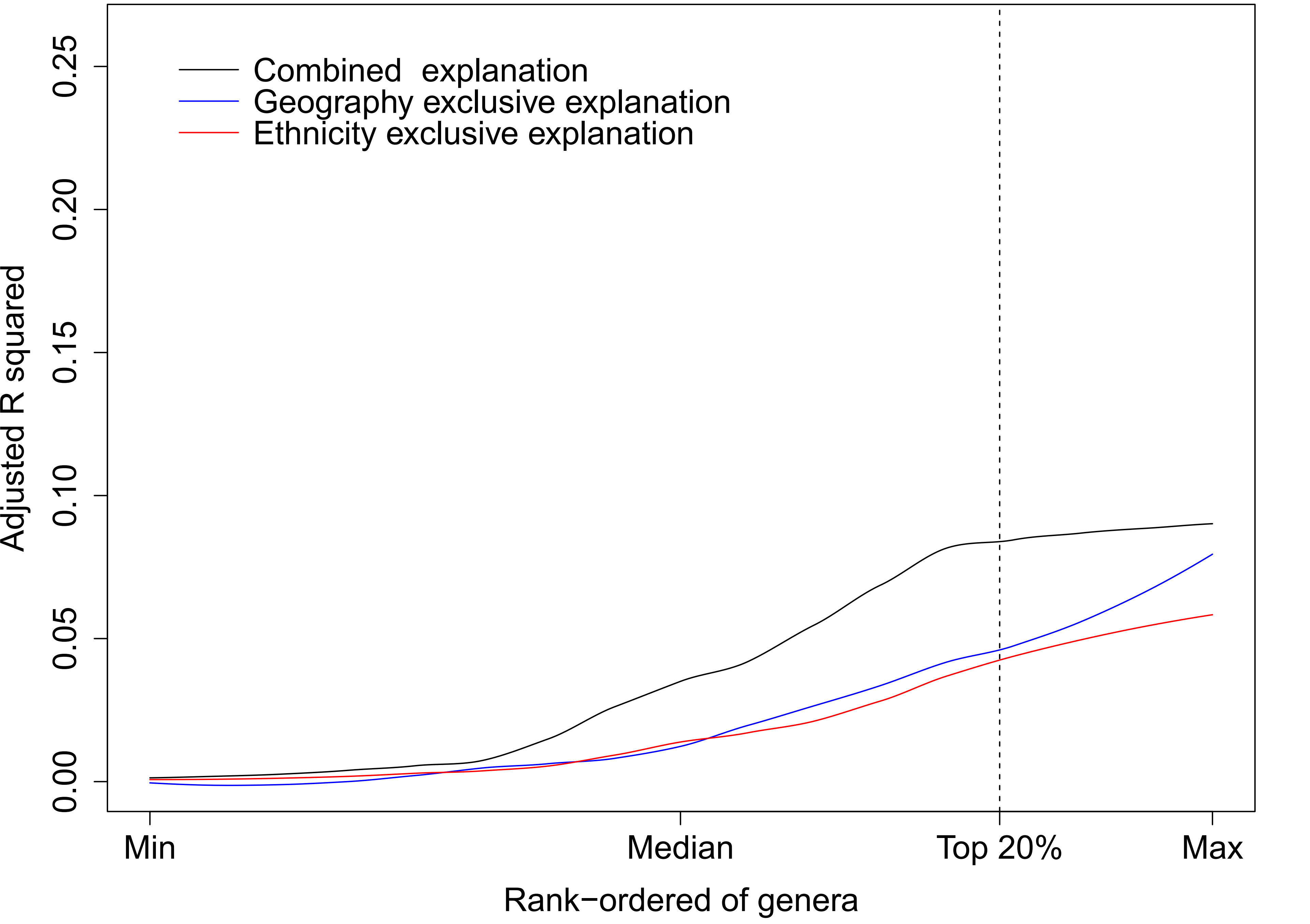
Combined explanation
Geography exclusive explanation
Ethnicity exclusive explanation
Adjusted R squared
0.00
0.05
0.10
0.15
0.20
0.25
Min
Median
Top 20%
Max
Rank−ordered of genera

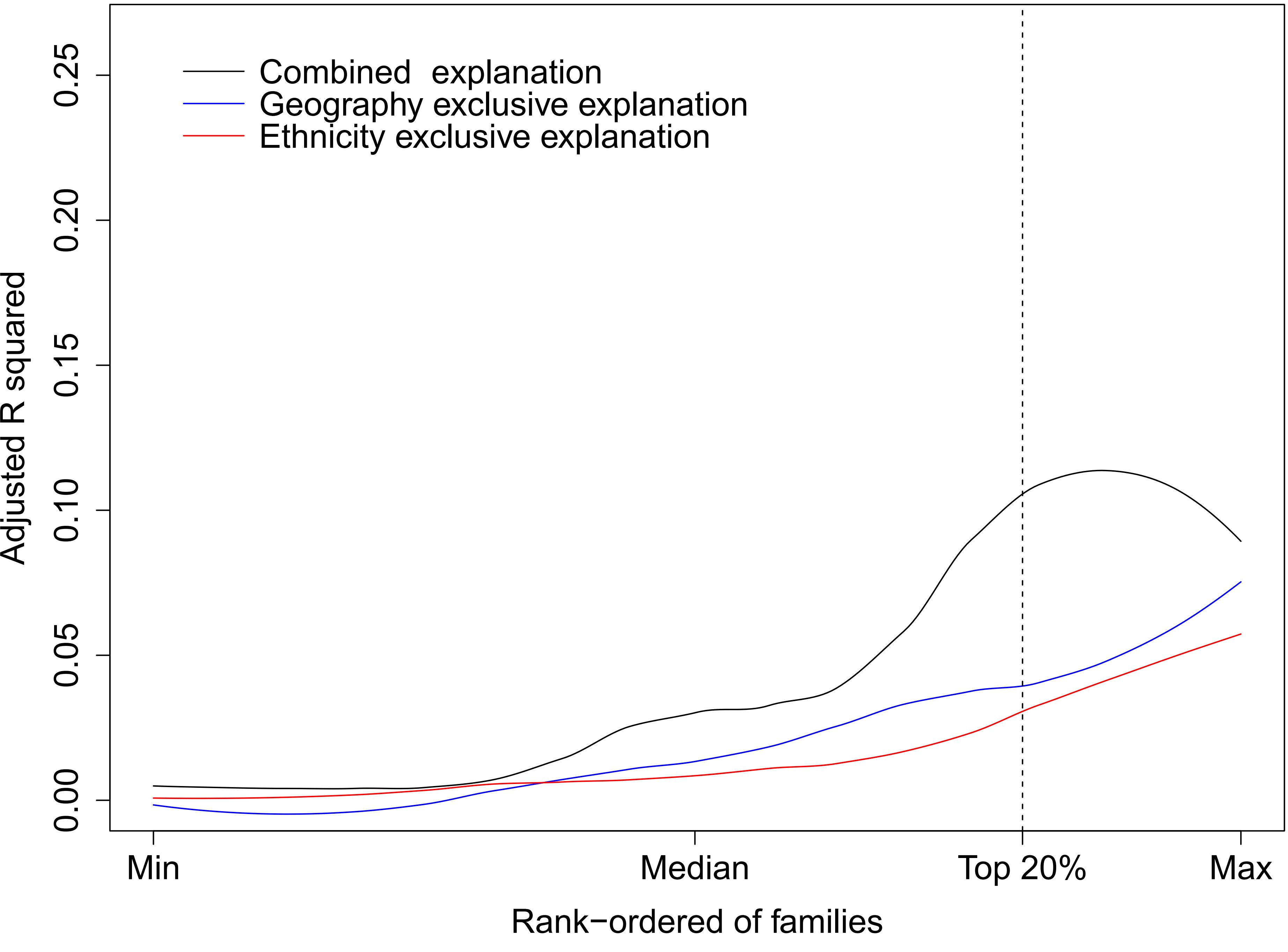

Combined explanation
Geography exclusive explanation
Ethnicity exclusive explanation
Adjusted R squared
0.00
0.05
0.10
0.15
0.20
0.25
Min
Median
Top 20%
Max
Rank−ordered of families

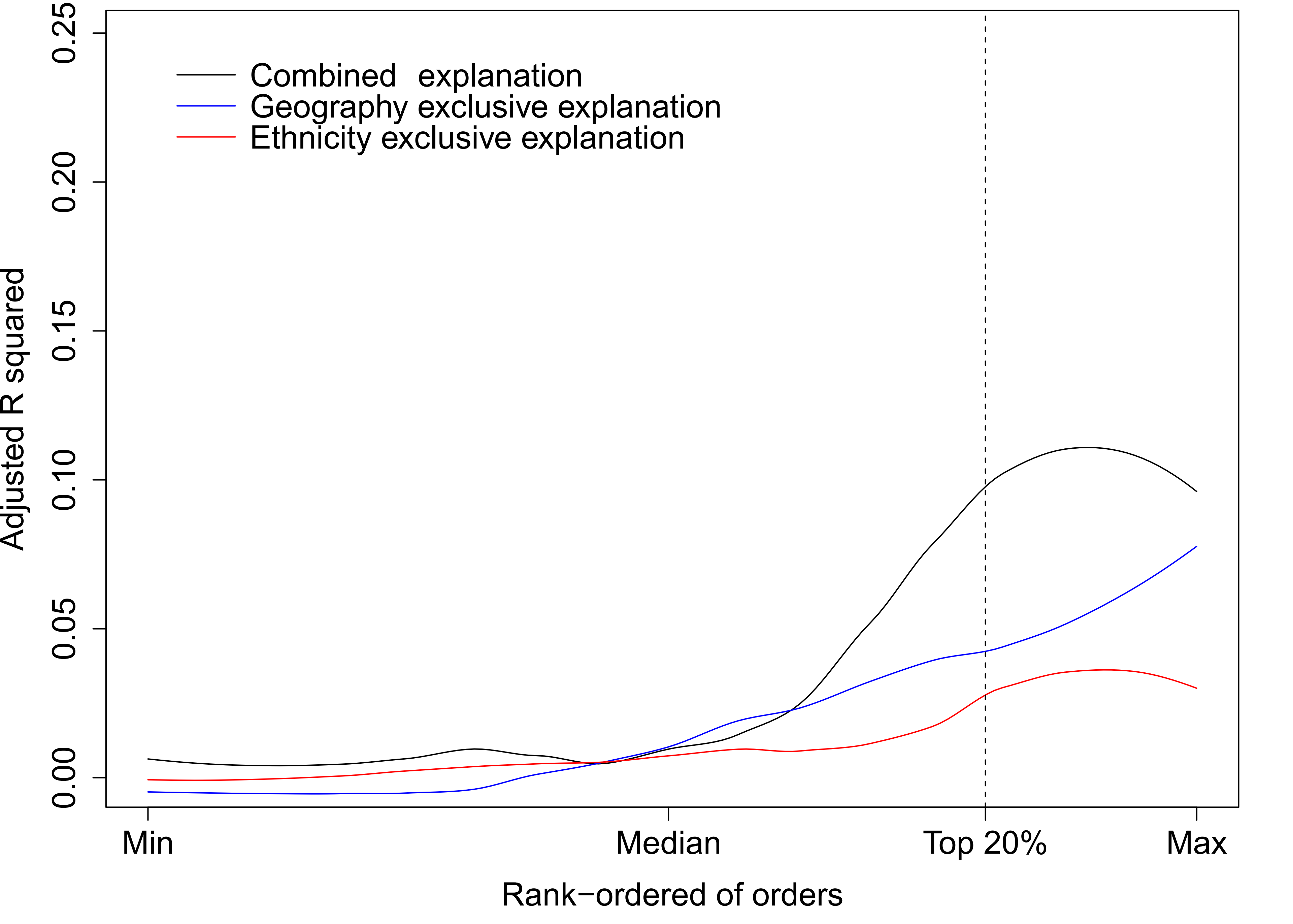

Combined explanation
Geography exclusive explanation
Ethnicity exclusive explanation
Adjusted R squared
0.00
0.05
0.10
0.15
0.20
0.25
Min
Median
Top 20%
Max
Rank−ordered of orders

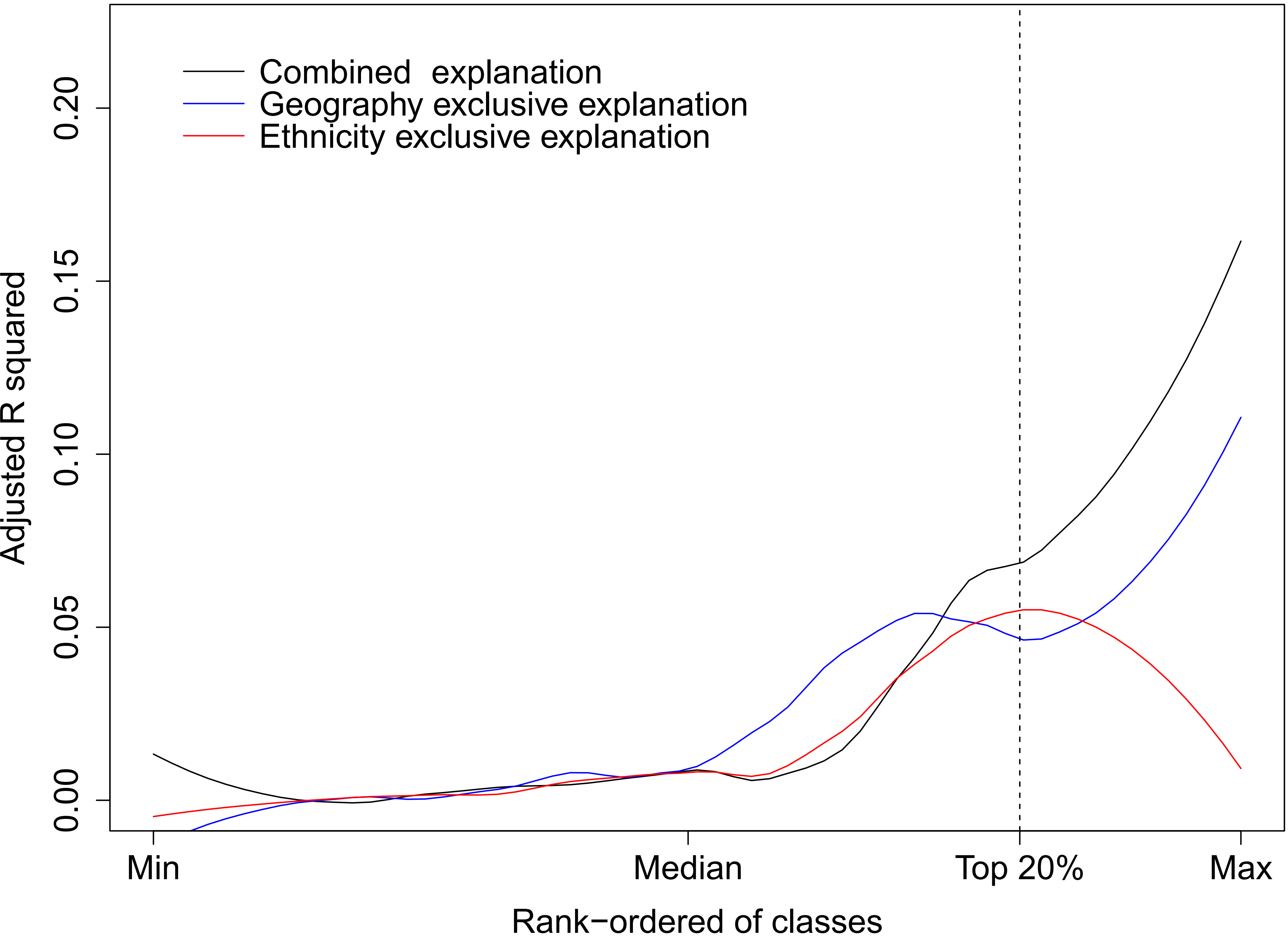

Combined explanation
Geography exclusive explanation
Ethnicity exclusive explanation
Adjusted R squared
0.00
0.05
0.10
0.15
0.20
Min
Median
Top 20%
Max
Rank−ordered of classes

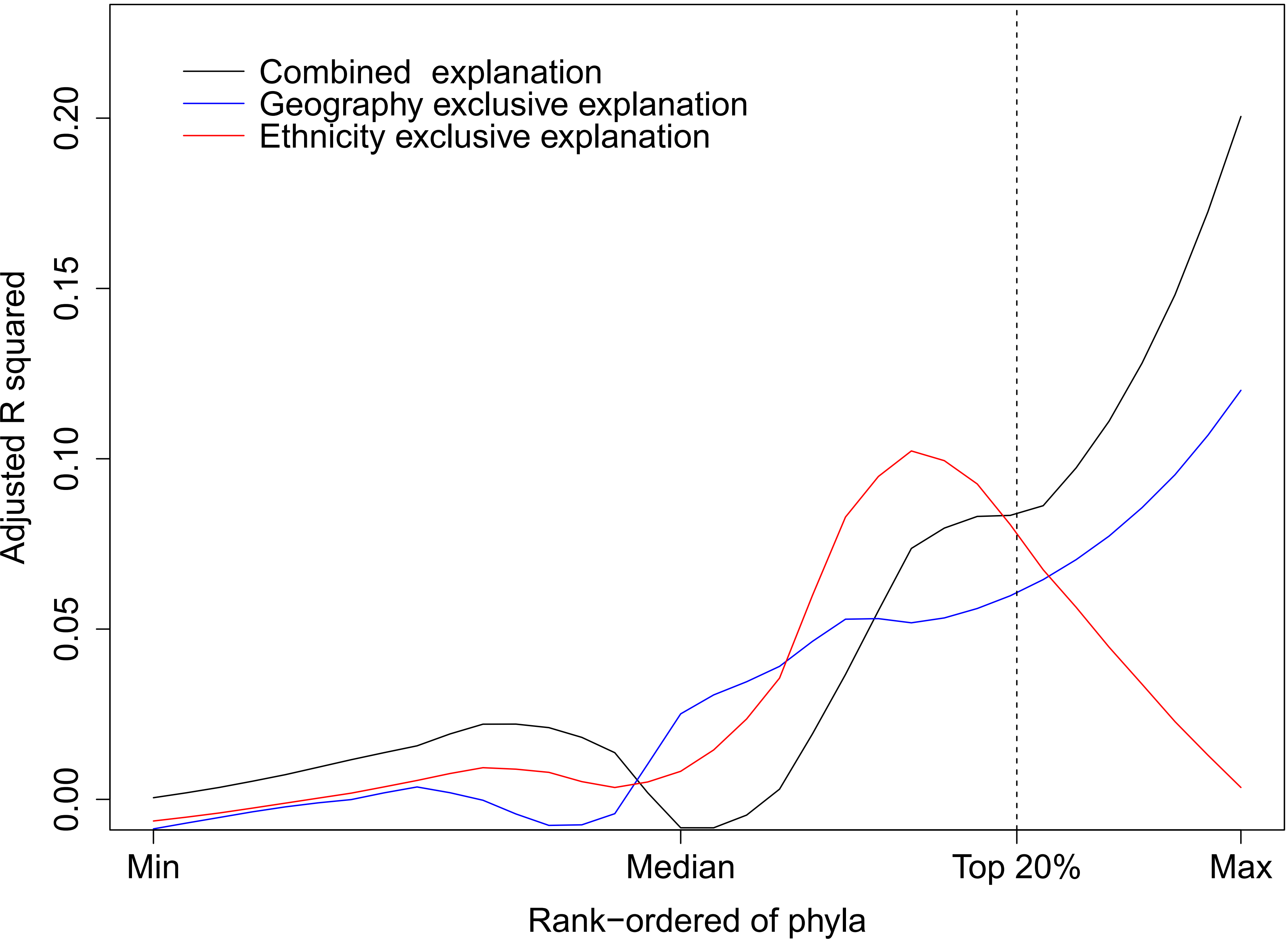
Combined explanation
Geography exclusive explanation
Ethnicity exclusive explanation
Adjusted R squared
0.00
0.05
0.10
0.15
0.20
Min
Median
Top 20%
Max
Rank−ordered of phyla

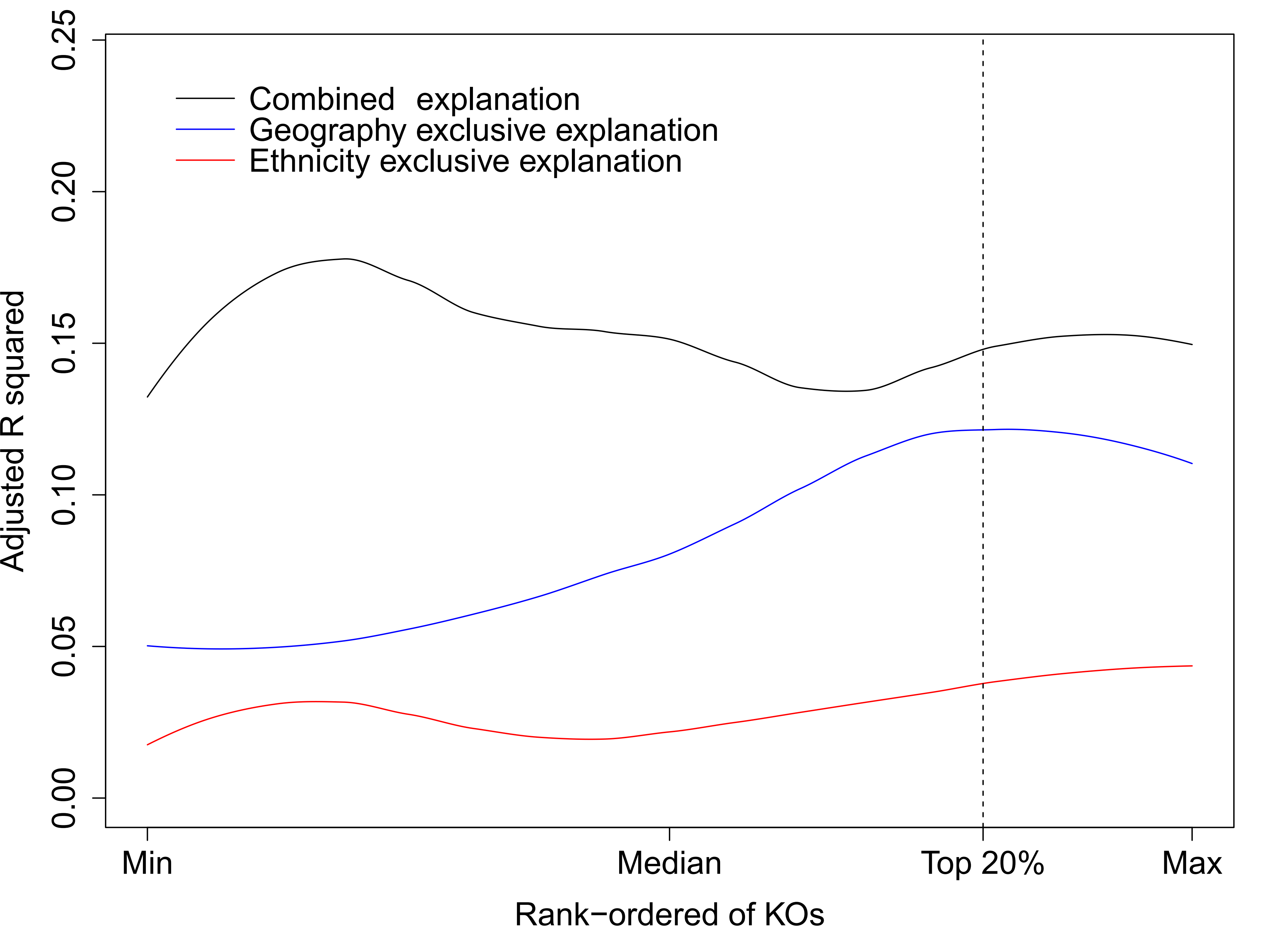

Combined explanation
Geography exclusive explanation
Ethnicity exclusive explanation
Adjusted R squared
0.00
0.05
0.10
0.15
0.20
0.25
Min
Median
Top 20%
Max
Rank−ordered of KOs
