# Supplementary material for: The Core and Distinction of the Gut Microbiota in Chinese Populations across Geography and Ethnicity
Source: Microorganisms. 2020 Oct 14;8(10):1579. doi: 10.3390/microorganisms8101579 (PMC7602275; doi:10.3390/microorganisms8101579)
Supplement: Supplementary file 1 [file microorganisms-08-01579-s001.zip › Supplement/Figure S3 Results of ANOCOV.pdf]

Fig.S3 Pvalue of of all bacterium at each taxonomic level and KOs between geographic location and ethnicity by two-ways ANOCOV ; According to Tablle S4, Y-axis : Pvalue of geography (p1); X-axis : Pvalue of ethnicity (p2); Pvalue of comparison in geography (p3); Pvalue of comparison in ethnicity (p4) .●: $p3 < 0.05$ and $p4 < 0.05$; ▲: $p3 \geq 0.05$ and $p4 < 0.05$; * : $p3 < 0.05$ and $p4 \geq 0.05$; ⊗ : $p3 \geq 0.05$ and $p4 \geq 0.05$.

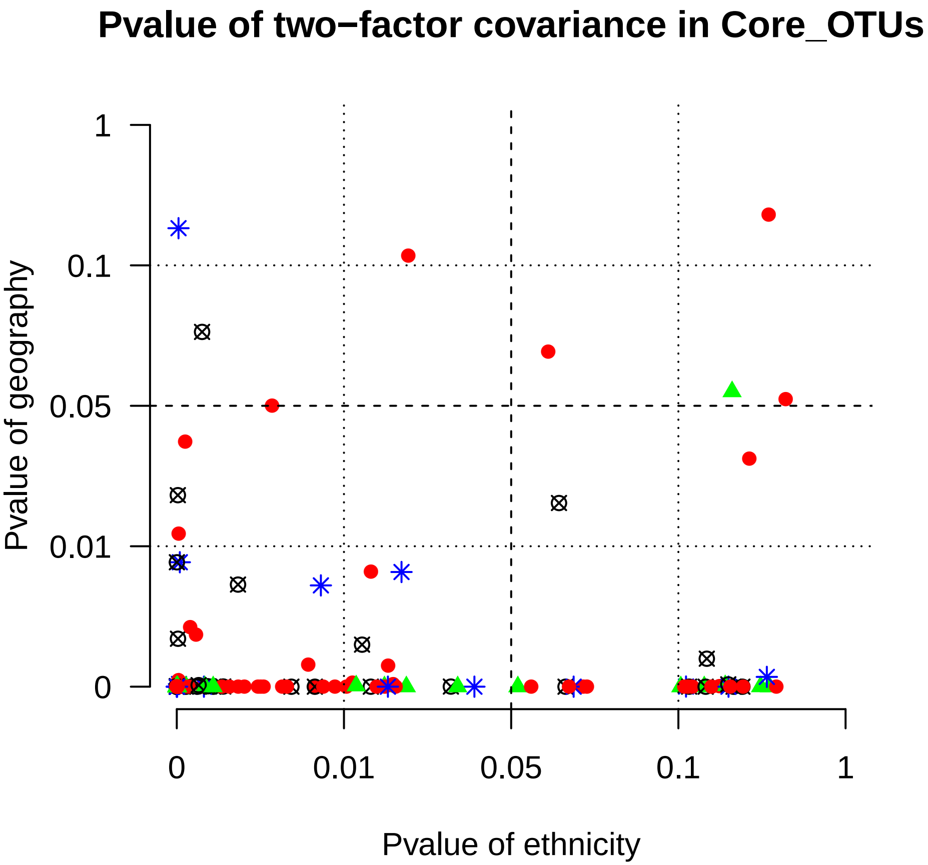

Pvalue of two−factor covariance in Core_OTUs
Pvalue of geography
Pvalue of ethnicity
1
0.1
0.05
0.01
0
0
0.01
0.05
0.1
1

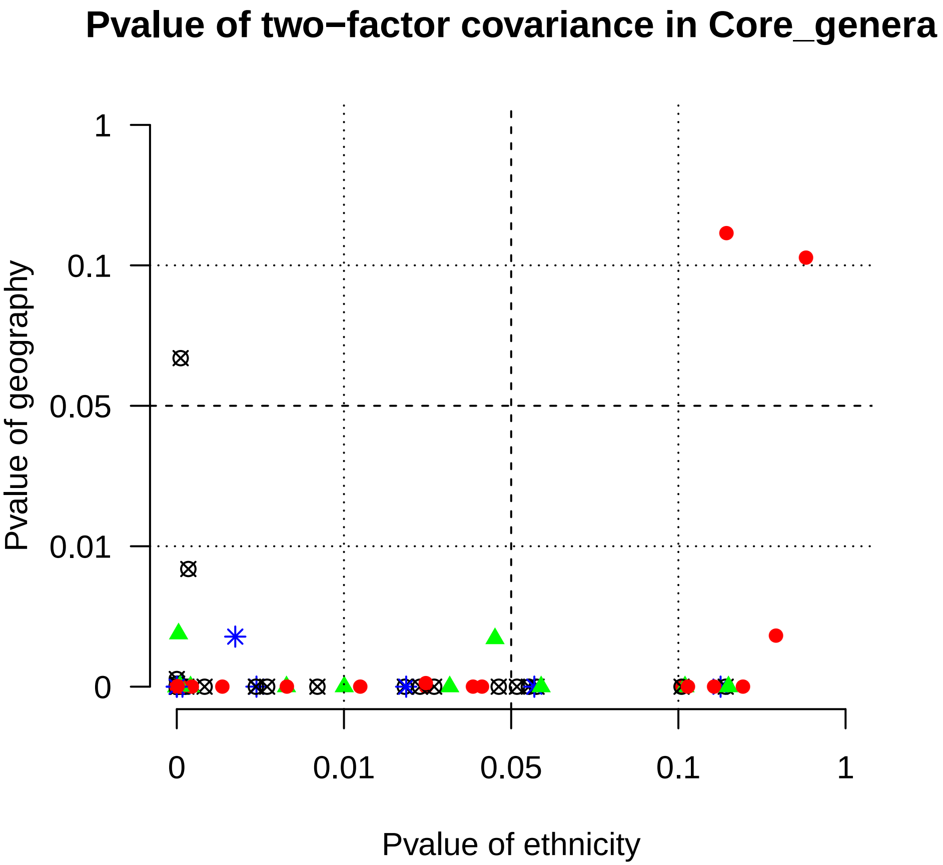

Pvalue of two−factor covariance in Core_genera
Pvalue of geography
1
0.1
0.05
0.01
0
Pvalue of ethnicity
0
0.01
0.05
0.1
1

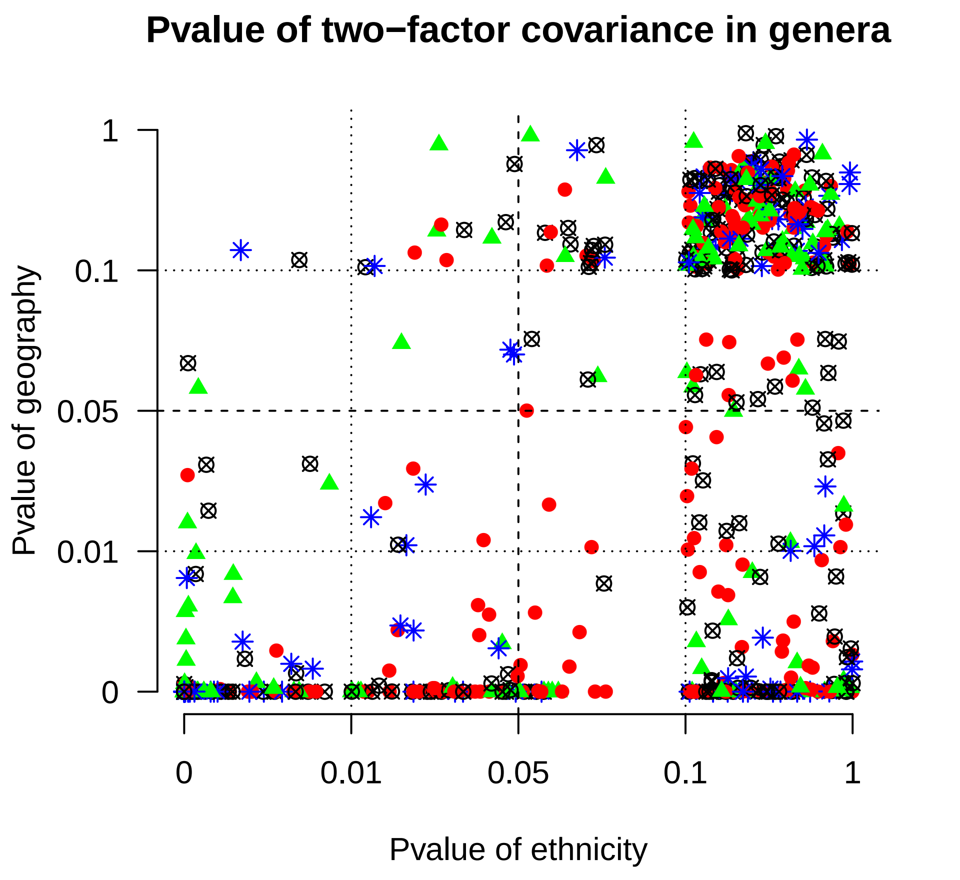
Pvalue of two−factor covariance in genera
Pvalue of geography
1
0.1
0.05
0.01
0
Pvalue of ethnicity
0
0.01
0.05
0.1
1

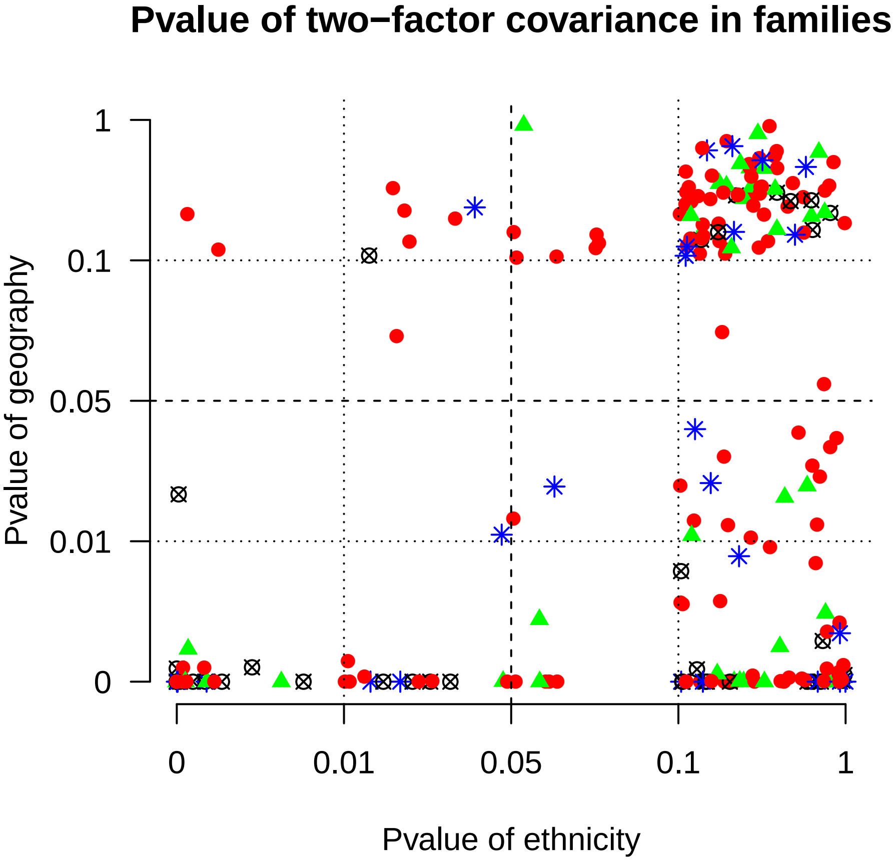

Pvalue of two−factor covariance in families
1
0.1
0.05
0.01
0
Pvalue of geography
0
0.01
0.05
0.1
1
Pvalue of ethnicity

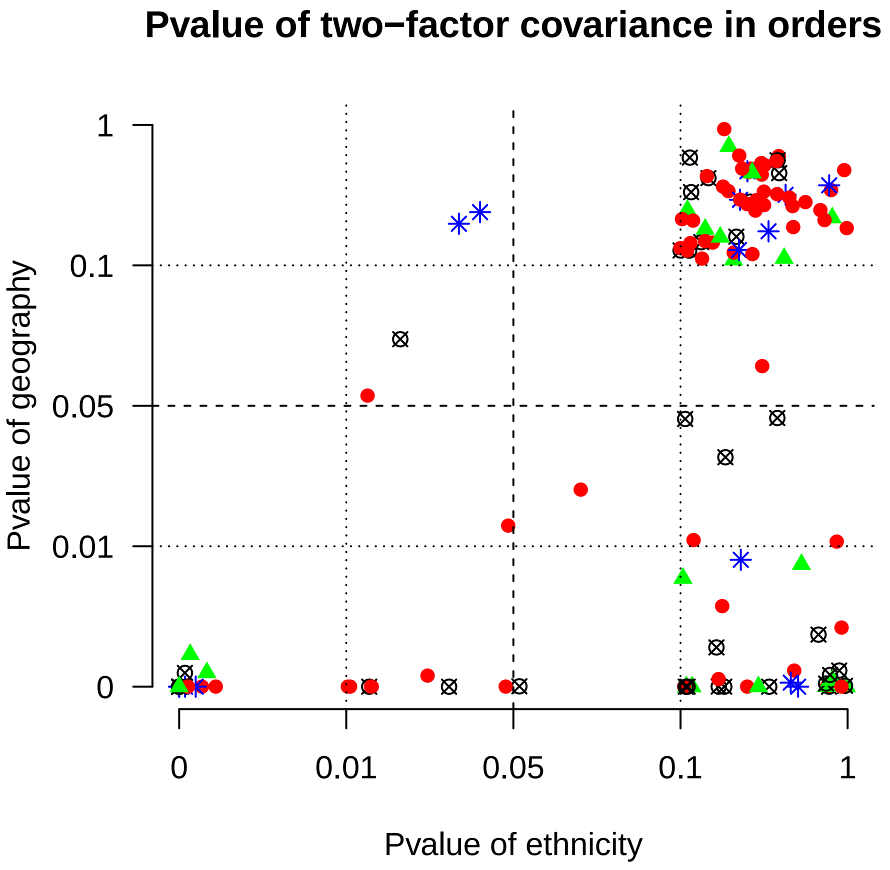
Pvalue of two−factor covariance in orders
Pvalue of geography
Pvalue of ethnicity
1
0.1
0.05
0.01
0
0
0.01
0.05
0.1
1

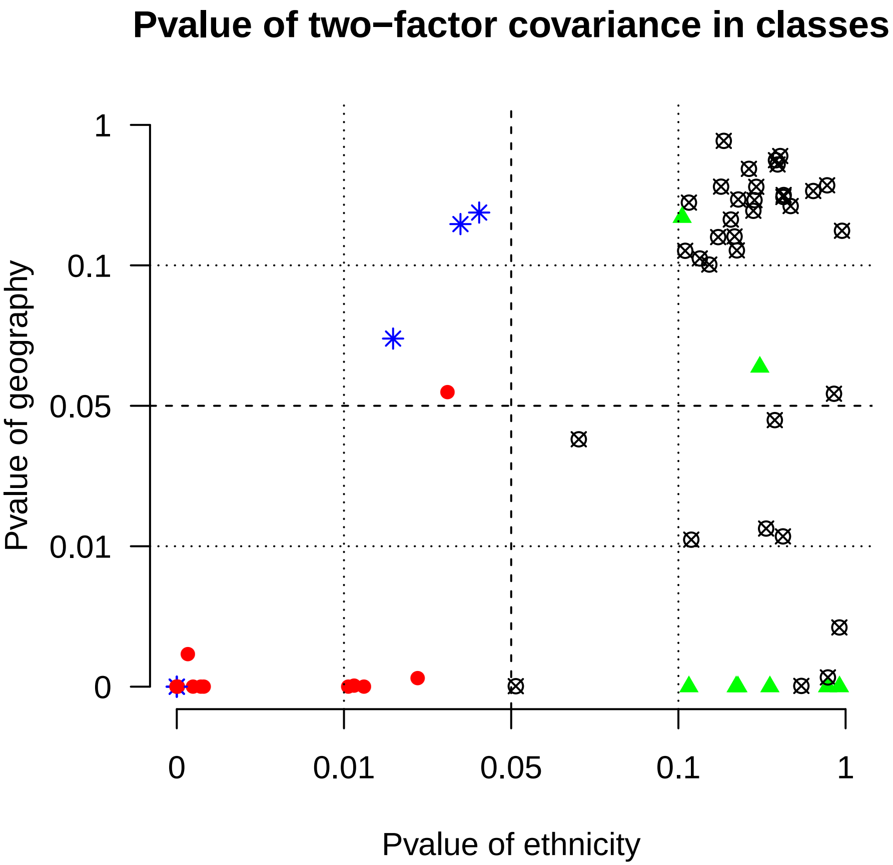

Pvalue of two−factor covariance in classes
Pvalue of geography
1
0.1
0.05
0.01
0
0
0.01
0.05
0.1
1
Pvalue of ethnicity

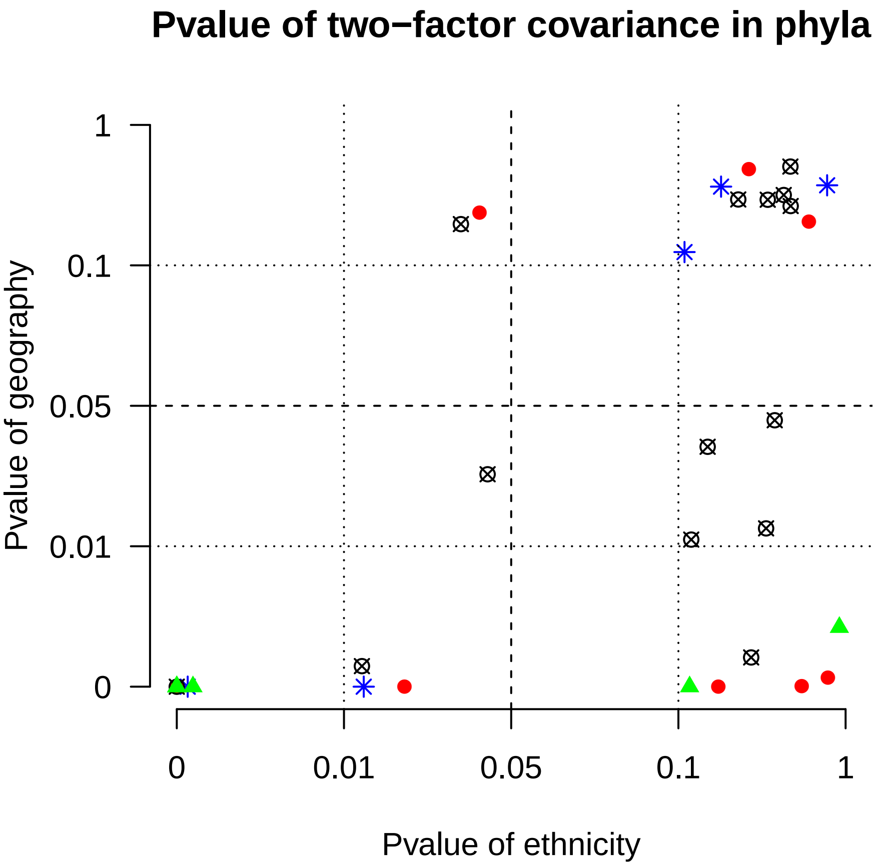
Pvalue of two−factor covariance in phyla
Pvalue of geography
1
0.1
0.05
0.01
0
Pvalue of ethnicity
0
0.01
0.05
0.1
1

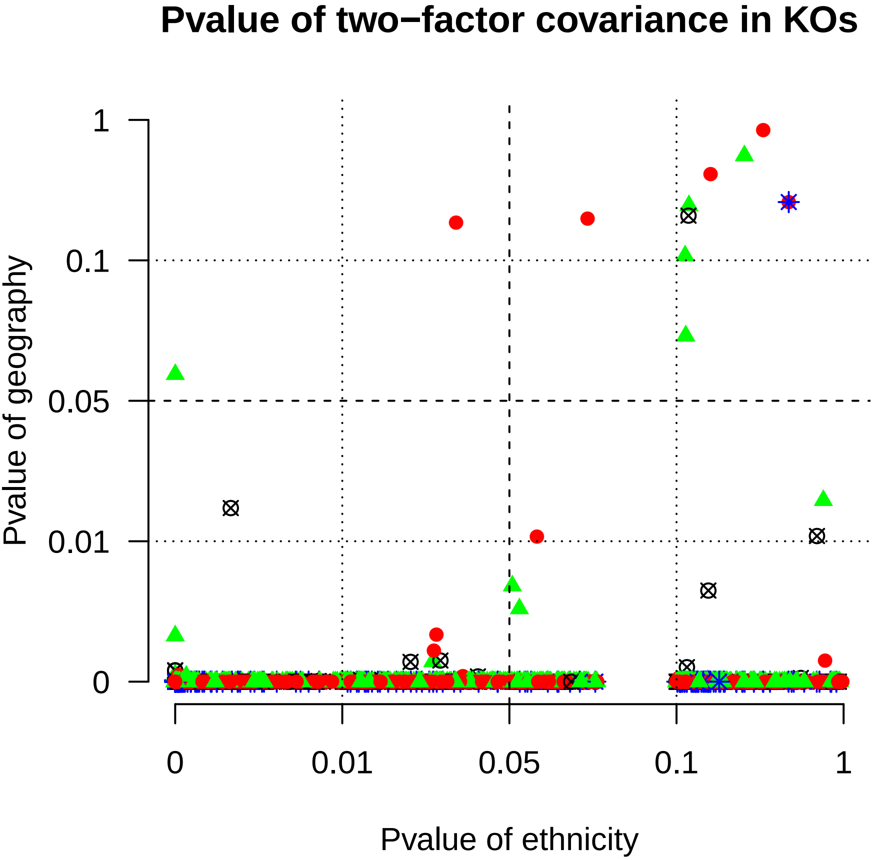
Pvalue of two−factor covariance in KOs
Pvalue of geography
1
0.1
0.05
0.01
0
0
0.01
0.05
0.1
1
Pvalue of ethnicity
