# Supplementary material for: The Core and Distinction of the Gut Microbiota in Chinese Populations across Geography and Ethnicity
Source: Microorganisms. 2020 Oct 14;8(10):1579. doi: 10.3390/microorganisms8101579 (PMC7602275; doi:10.3390/microorganisms8101579)
Supplement: Supplementary file 1 [file microorganisms-08-01579-s001.zip › Supplement/Figure S4 LEfSe.pdf]

Detected biomarkers by LEfSe analyses

LEfSe was used for comparing characteristic taxonomy among Han to non-Han in all groups; Hans to another Hans in different location; non-Hans to another non-Hans in different location; Hans to non-Hans in same location. Finally, biomarkers were obtained by four steps as follow.

Step 1: the Kruskall-Wallis test analyzes all features, testing whether the values in different classes are differentially distributed. Features violating the null hypothesis are further analyzed in Step 2. The significant threshold of pvalue ≤ 0.05 in Kruskall-Wallis test.

Step 2: the pairwise Wilcoxon test checks whether all pairwise comparisons between subclasses within different classes significantly agree with the class level trend. The significant threshold of pvalue ≤ 0.05 in Wilcoxon test.

Step 3: the resulting subset of vectors is used to build a Linear Discriminant Analysis model from which the relative difference among classes is used to rank the features. The final output thus consists of a list of features that are discriminative with respect to the classes, consistent with the subclass grouping within classes, and ranked according to the effect size with which they differentiate classes. The significant threshold of LDA SCORE(log10) > 2.

Step 4: Based on Step 3, the representative biomakers of Hans in a certain location must be the largest or smallest in all different locations, and there are significant differences compared with other locations. Similarly, the representative biomakers of non-Hans in a certain location must be the largest or smallest in all different locations, and there are significant differences compared with other locations.

Results as follow:

Differences are represented in the color of the most abundant class (Red and green represent different geographic location or ethnicity); LEfSe provides features that were differential among conditions of interest with statistical and biological significance, ranking them according to the effect size.

Hans to another Hans in different location: Pages 2-22

non-Hans to another non-Hans in different location: Pages 23-43

Hans to non-Hans in same location: Pages 44-50

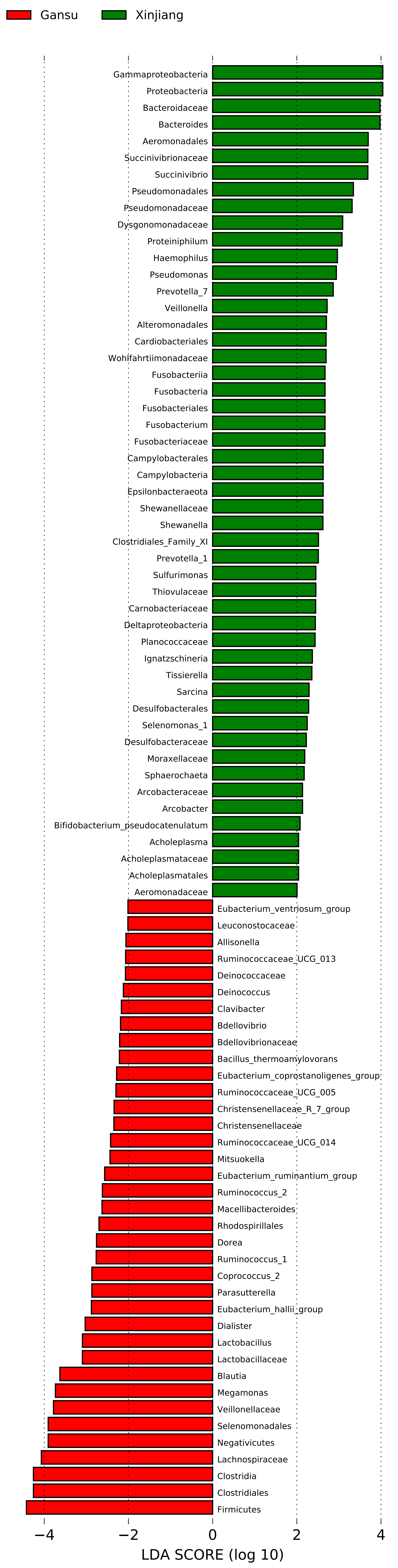

Gansu
Xinjiang
Gammaproteobacteria
Proteobacteria
Bacteroidaceae
Bacteroides
Aeromonadales
Succinivibrionaceae
Succinivibrio
Pseudomonadales
Pseudomonadaceae
Dysgonomonadaceae
Proteiniphilum
Haemophilus
Pseudomonas
Prevotella_7
Veillonella
Alteromonadales
Cardiobacteriales
Wohlfahrtiimonadaceae
Fusobacteriia
Fusobacteria
Fusobacteriales
Fusobacterium
Fusobacteriaceae
Campylobacterales
Campylobacteria
Epsilonbacteraeota
Shewanellaceae
Shewanella
Clostridiales_Family_XI
Prevotella_1
Sulfurimonas
Thiovulaceae
Carnobacteriaceae
Deltaproteobacteria
Planococcaceae
Ignatzschineria
Tissierella
Sarcina
Desulfobacterales
Selenomonas_1
Desulfobacteraceae
Moraxellaceae
Sphaerochaeta
Arcobacteraceae
Arcobacter
Bifidobacterium_pseudocatenulatum
Acholeplasma
Acholeplasmataceae
Acholeplasmatales
Aeromonadaceae
Eubacterium_ventriosum_group
Leuconostocaceae
Allisonella
Ruminococcaceae_UCG_013
Deinococcaceae
Deinococcus
Clavibacter
Bdellovibrio
Bdellovibrionaceae
Bacillus_thermoamylovorans
Eubacterium_coprostanoligenes_group
Ruminococcaceae_UCG_005
Christensenellaceae_R_7_group
Christensenellaceae
Ruminococcaceae_UCG_014
Mitsuokella
Eubacterium_ruminantium_group
Ruminococcus_2
Macellibacteroides
Rhodospirillales
Dorea
Ruminococcus_1
Coprococcus_2
Parasutterella
Eubacterium_hallii_group
Dialister
Lactobacillus
Lactobacillaceae
Blautia
Megamonas
Veillonellaceae
Selenomonadales
Negativicutes
Lachnospiraceae
Clostridia
Clostridiales
Firmicutes
−4
−2
0
2
4
LDA SCORE (log 10)

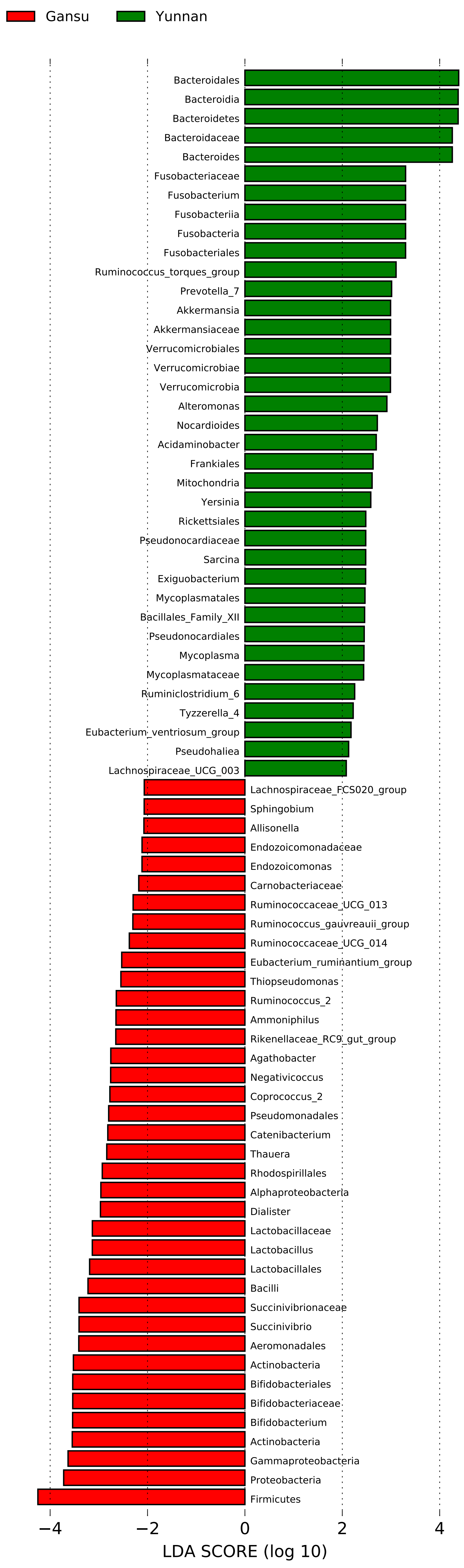
Gansu
Yunnan
Bacteroidales
Bacteroidia
Bacteroidetes
Bacteroidaceae
Bacteroides
Fusobacteriaceae
Fusobacterium
Fusobacteriia
Fusobacteria
Fusobacteriales
Ruminococcus_torques_group
Prevotella_7
Akkermansia
Akkermansiaceae
Verrucomicrobiales
Verrucomicrobiae
Verrucomicrobia
Alteromonas
Nocardioides
Acidaminobacter
Frankiales
Mitochondria
Yersinia
Rickettsiales
Pseudonocardiaceae
Sarcina
Exiguobacterium
Mycoplasmatales
Bacillales_Family_XII
Pseudonocardiales
Mycoplasma
Mycoplasmataceae
Ruminiclostridium_6
Tyzzerella_4
Eubacterium_ventriosum_group
Pseudohaliea
Lachnospiraceae_UCG_003
Lachnospiraceae_FCS020_group
Sphingobium
Allisonella
Endozoicomonadaceae
Endozoicomonas
Carnobacteriaceae
Ruminococcaceae_UCG_013
Ruminococcus_gauvreauii_group
Ruminococcaceae_UCG_014
Eubacterium_ruminantium_group
Thiopseudomonas
Ruminococcus_2
Ammoniphilus
Rikenellaceae_RC9_gut_group
Agathobacter
Negativicoccus
Coprococcus_2
Pseudomonadales
Catenibacterium
Thauera
Rhodospirillales
Alphaproteobacteria
Dialister
Lactobacillaceae
Lactobacillus
Lactobacillales
Bacilli
Succinivibrionaceae
Succinivibrio
Aeromonadales
Actinobacteria
Bifidobacteriales
Bifidobacteriaceae
Bifidobacterium
Actinobacteria
Gammaproteobacteria
Proteobacteria
Firmicutes
−4
−2
0
2
4
LDA SCORE (log 10)

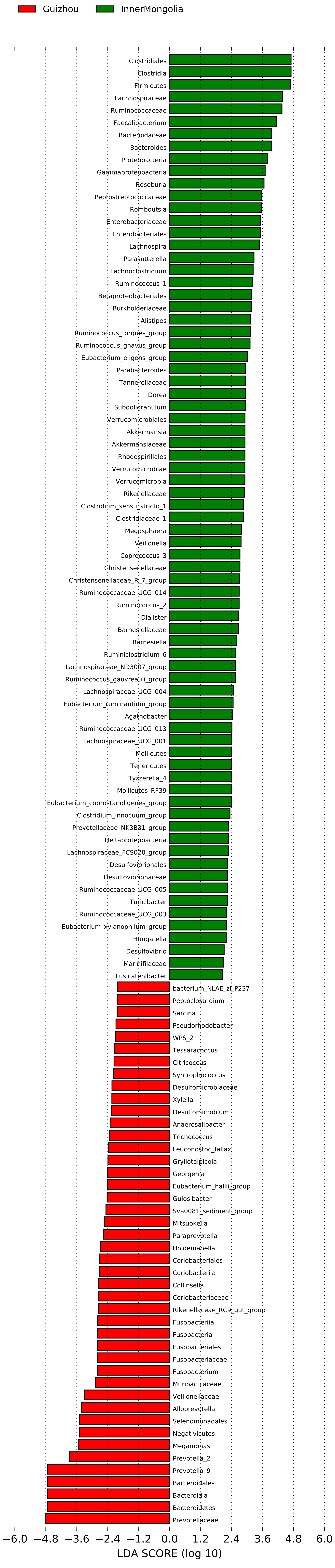

Guizhou
InnerMongolia
Clostridiales
Clostridia
Firmicutes
Lachnospiraceae
Ruminococcaceae
Faecalibacterium
Bacteroidaceae
Bacteroides
Proteobacteria
Gammaproteobacteria
Roseburia
Peptostreptococcaceae
Romboutsia
Enterobacteriaceae
Enterobacteriales
Lachnospira
Parasutterella
Lachnoclostridium
Ruminococcus_1
Betaproteobacteriales
Burkholderiaceae
Alistipes
Ruminococcus_torques_group
Ruminococcus_gnavus_group
Eubacterium_eligens_group
Parabacteroides
Tannerellaceae
Dorea
Subdoligranulum
Verrucomicrobiales
Akkermansia
Akkermansiaceae
Rhodospirillales
Verrucomicrobiae
Verrucomicrobia
Rikenellaceae
Clostridium_sensu_stricto_1
Clostridiaceae_1
Megasphaera
Veillonella
Coprococcus_3
Christensenellaceae
Christensenellaceae_R_7_group
Ruminococcaceae_UCG_014
Ruminococcus_2
Dialister
Barnesiellaceae
Barnesiella
Ruminiclostridium_6
Lachnospiraceae_ND3007_group
Ruminococcus_gauvreauii_group
Lachnospiraceae_UCG_004
Eubacterium_ruminantium_group
Agathobacter
Ruminococcaceae_UCG_013
Lachnospiraceae_UCG_001
Mollicutes
Tenericutes
Tyzzerella_4
Mollicutes_RF39
Eubacterium_coprostanoligenes_group
Clostridium_innocuum_group
Prevotellaceae_NK3B31_group
Deltaproteobacteria
Lachnospiraceae_FCS020_group
Desulfovibrionales
Desulfovibrionaceae
Ruminococcaceae_UCG_005
Turicibacter
Ruminococcaceae_UCG_003
Eubacterium_xylanophilum_group
Hungatella
Desulfovibrio
Marinifilaceae
Fusicatenibacter
bacterium_NLAE_zl_P237
Peptoclostridium
Sarcina
Pseudorhodobacter
WPS_2
Tessaracoccus
Citricoccus
Syntrophococcus
Desulfomicrobiaceae
Xylella
Desulfomicrobium
Anaerosalibacter
Trichococcus
Leuconostoc_fallax
Gryllotalpicola
Georgenia
Eubacterium_hallii_group
Gulosibacter
Sva0081_sediment_group
Mitsuokella
Paraprevotella
Holdemanella
Coriobacteriales
Coriobacteriia
Collinsella
Coriobacteriaceae
Rikenellaceae_RC9_gut_group
Fusobacteriia
Fusobacteria
Fusobacteriales
Fusobacteriaceae
Fusobacterium
Muribaculaceae
Veillonellaceae
Alloprevotella
Selenomonadales
Negativicutes
Megamonas
Prevotella_2
Prevotella_9
Bacteroidales
Bacteroidia
Bacteroidetes
Prevotellaceae
−6.0
−4.8
−3.6
−2.4
−1.2
0.0
1.2
2.4
3.6
4.8
6.0
LDA SCORE (log 10)

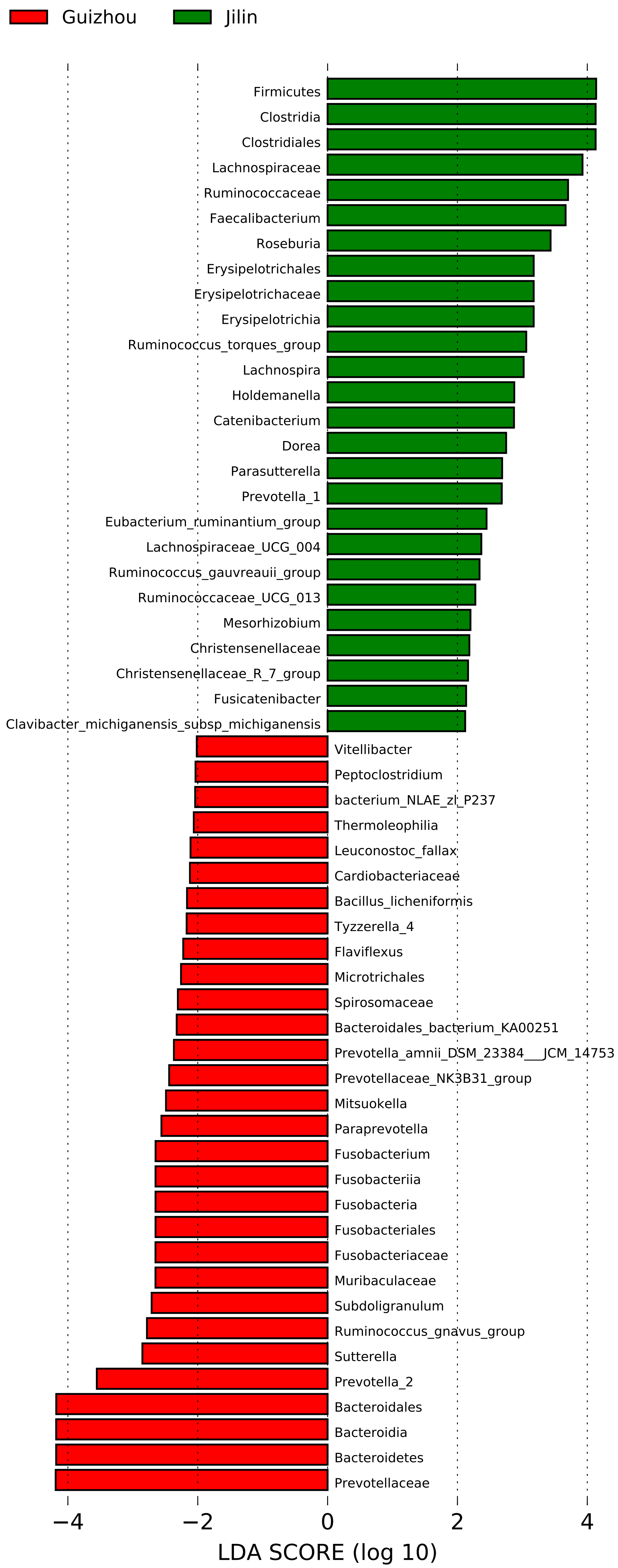

Guizhou
Jilin
Firmicutes
Clostridia
Clostridiales
Lachnospiraceae
Ruminococcaceae
Faecalibacterium
Roseburia
Erysipelotrichales
Erysipelotrichaceae
Erysipelotrichia
Ruminococcus_torques_group
Lachnospira
Holdemanella
Catenibacterium
Dorea
Parasutterella
Prevotella_1
Eubacterium_ruminantium_group
Lachnospiraceae_UCG_004
Ruminococcus_gauvreauii_group
Ruminococcaceae_UCG_013
Mesorhizobium
Christensenellaceae
Christensenellaceae_R_7_group
Fusicatenibacter
Clavibacter_michiganensis_subsp_michiganensis
Vitellibacter
Peptoclostridium
bacterium_NLAE_zl_P237
Thermoleophilia
Leuconostoc_fallax
Cardiobacteriaceae
Bacillus_licheniformis
Tyzzerella_4
Flaviflexus
Microtrichales
Spirosomaceae
Bacteroidales_bacterium_KA00251
Prevotella_amnii_DSM_23384___JCM_14753
Prevotellaceae_NK3B31_group
Mitsuokella
Paraprevotella
Fusobacterium
Fusobacteriia
Fusobacteria
Fusobacteriales
Fusobacteriaceae
Muribaculaceae
Subdoligranulum
Ruminococcus_gnavus_group
Sutterella
Prevotella_2
Bacteroidales
Bacteroidia
Bacteroidetes
Prevotellaceae
−4
−2
0
2
4
LDA SCORE (log 10)

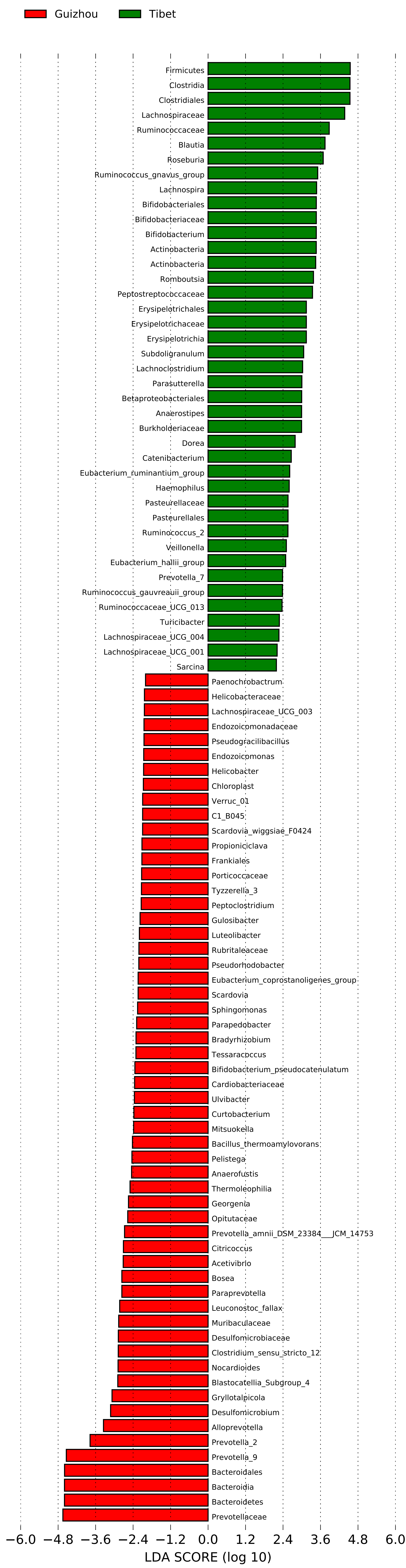
Guizhou
Tibet
Firmicutes
Clostridia
Clostridiales
Lachnospiraceae
Ruminococcaceae
Blautia
Roseburia
Ruminococcus_gnavus_group
Lachnospira
Bifidobacteriales
Bifidobacteriaceae
Bifidobacterium
Actinobacteria
Actinobacteria
Romboutsia
Peptostreptococcaceae
Erysipelotrichales
Erysipelotrichaceae
Erysipelotrichia
Subdoligranulum
Lachnoclostridium
Parasutterella
Betaproteobacteriales
Anaerostipes
Burkholderiaceae
Dorea
Catenibacterium
Eubacterium_ruminantium_group
Haemophilus
Pasteurellaceae
Pasteurellales
Ruminococcus_2
Veillonella
Eubacterium_hallii_group
Prevotella_7
Ruminococcus_gauvreauii_group
Ruminococcaceae_UCG_013
Turicibacter
Lachnospiraceae_UCG_004
Lachnospiraceae_UCG_001
Sarcina
Paenochrobactrum
Helicobacteraceae
Lachnospiraceae_UCG_003
Endozoicomonadaceae
Pseudogracilibacillus
Endozoicomonas
Helicobacter
Chloroplast
Verruc_01
C1_B045
Scardovia_wiggsiae_F0424
Propioniciclava
Frankiales
Porticoccaceae
Tyzzerella_3
Peptoclostridium
Gulosibacter
Luteolibacter
Rubritaleaceae
Pseudorhodobacter
Eubacterium_coprostanoligenes_group
Scardovia
Sphingomonas
Parapedobacter
Bradyrhizobium
Tessaracoccus
Bifidobacterium_pseudocatenulatum
Cardiobacteriaceae
Ulvibacter
Curtobacterium
Mitsuokella
Bacillus_thermoamylovorans
Pelistega
Anaerofustis
Thermoleophilia
Georgenia
Opitutaceae
Prevotella_amnii_DSM_23384__JCM_14753
Citricoccus
Acetivibrio
Bosea
Paraprevotella
Leuconostoc_fallax
Muribaculaceae
Desulfomicrobiaceae
Clostridium_sensu_stricto_12
Nocardioides
Blastocatellia_Subgroup_4
Gryllotalpicola
Desulfomicrobium
Alloprevotella
Prevotella_2
Prevotella_9
Bacteroidales
Bacteroidia
Bacteroidetes
Prevotellaceae
−6.0 −4.8 −3.6 −2.4 −1.2 0.0 1.2 2.4 3.6 4.8 6.0
LDA SCORE (log 10)

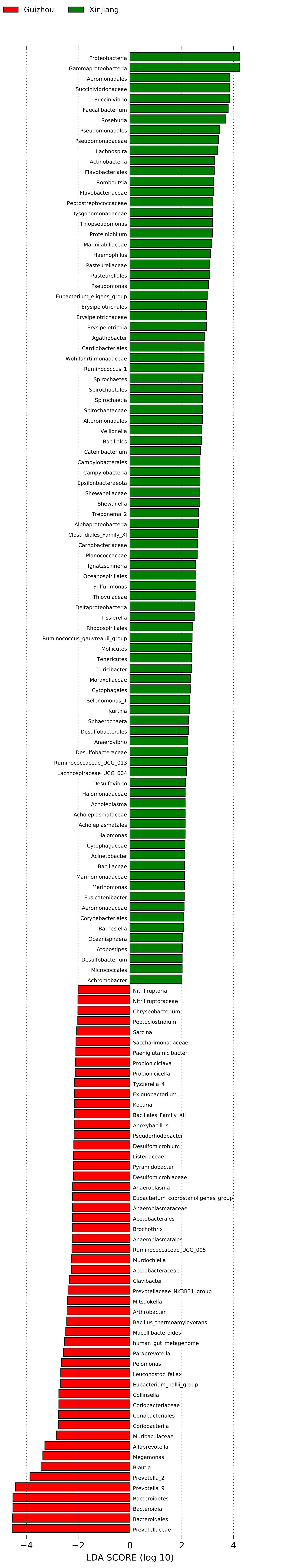
Guizhou
Xinjiang
Proteobacteria
Gammaproteobacteria
Aeromonadales
Succinivibrionaceae
Succinivibrio
Faecalibacterium
Roseburia
Pseudomonadales
Pseudomonadaceae
Lachnospira
Actinobacteria
Flavobacteriales
Romboutsia
Flavobacteriaceae
Peptostreptococcaceae
Dysgonomonadaceae
Thiopseudomonas
Proteiniphilum
Marinilabiliaceae
Haemophilus
Pasteurellaceae
Pasteurellales
Pseudomonas
Eubacterium_eligens_group
Erysipelotrichales
Erysipelotrichaceae
Erysipelotrichia
Agathobacter
Cardiobacteriales
Wohlfahrtiimonadaceae
Ruminococcus_1
Spirochaetes
Spirochaetales
Spirochaetia
Spirochaetaceae
Alteromonadales
Veillonella
Bacillales
Catenibacterium
Campylobacterales
Campylobacteria
Epsilonbacteraeota
Shewanellaceae
Shewanella
Treponema_2
Alphaproteobacteria
Clostridiales_Family_XI
Carnobacteriaceae
Planococcaceae
Ignatzschineria
Oceanospirillales
Sulfurimonas
Thiovulaceae
Deltaproteobacteria
Tissierella
Rhodospirillales
Ruminococcus_gauvreauii_group
Mollicutes
Tenericutes
Turicibacter
Moraxellaceae
Cytophagales
Selenomonas_1
Kurthia
Sphaerochaeta
Desulfobacterales
Anaerovibrio
Desulfobacteraceae
Ruminococcaceae_UCG_013
Lachnospiraceae_UCG_004
Desulfovibrio
Halomonadaceae
Acholeplasma
Acholeplasmataceae
Acholeplasmatales
Halomonas
Cytophagaceae
Acinetobacter
Bacillaceae
Marinomonadaceae
Marinomonas
Fusicatenibacter
Aeromonadaceae
Corynebacteriales
Barnesiella
Oceanisphaera
Atopostipes
Desulfobacterium
Micrococcales
Achromobacter
Nitriliruptoria
Nitriliruptoraceae
Chryseobacterium
Peptoclostridium
Sarcina
Saccharimonadaceae
Paeniglutamicibacter
Propioniciclava
Propionicicella
Tyzzerella_4
Exiguobacterium
Kocuria
Bacillales_Family_XII
Anoxybacillus
Pseudorhodobacter
Desulfomicrobium
Listeriaceae
Pyramidobacter
Desulfomicrobiaceae
Anaeroplasma
Eubacterium_coprostanoligenes_group
Anaeroplasmataceae
Acetobacterales
Brochothrix
Anaeroplasmatales
Ruminococcaceae_UCG_005
Murdochiella
Acetobacteraceae
Clavibacter
Prevotellaceae_NK3B31_group
Mitsuokella
Arthrobacter
Bacillus_thermoamylovorans
Macellibacteroides
human_gut_metagenome
Paraprevotella
Pelomonas
Leuconostoc_fallax
Eubacterium_hallii_group
Collinsella
Coriobacteriaceae
Coriobacteriales
Coriobacteriia
Muribaculaceae
Alloprevotella
Megamonas
Blautia
Prevotella_2
Prevotella_9
Bacteroidetes
Bacteroidia
Bacteroidales
Prevotellaceae
−4
−2
0
2
4
LDA SCORE (log 10)

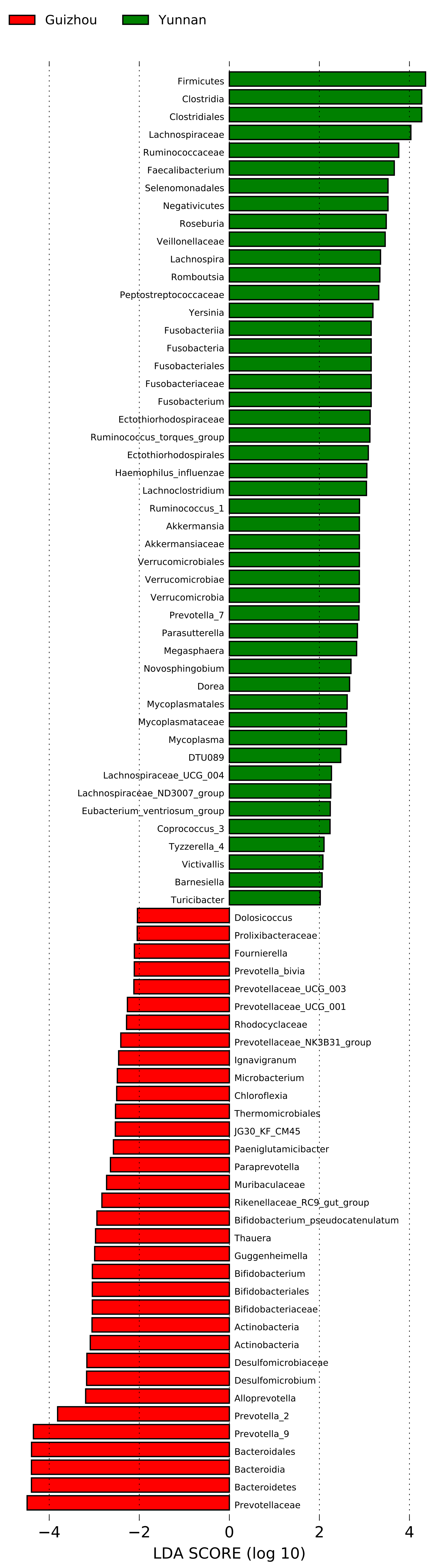

Guizhou
Yunnan
Firmicutes
Clostridia
Clostridiales
Lachnospiraceae
Ruminococcaceae
Faecalibacterium
Selenomonadales
Negativicutes
Roseburia
Veillonellaceae
Lachnospira
Romboutsia
Peptostreptococcaceae
Yersinia
Fusobacteriia
Fusobacteria
Fusobacteriales
Fusobacteriaceae
Fusobacterium
Ectothiorhodospiraceae
Ruminococcus_torques_group
Ectothiorhodospirales
Haemophilus_influenzae
Lachnoclostridium
Ruminococcus_1
Akkermansia
Akkermansiaceae
Verrucomicrobiales
Verrucomicrobiae
Verrucomicrobia
Prevotella_7
Parasutterella
Megasphaera
Novosphingobium
Dorea
Mycoplasmatales
Mycoplasmataceae
Mycoplasma
DTU089
Lachnospiraceae_UCG_004
Lachnospiraceae_ND3007_group
Eubacterium_ventriosum_group
Coprococcus_3
Tyzzerella_4
Victivallis
Barnesiella
Turicibacter
Dolosicoccus
Prolixibacteraceae
Fournierella
Prevotella_bivia
Prevotellaceae_UCG_003
Prevotellaceae_UCG_001
Rhodocyclaceae
Prevotellaceae_NK3B31_group
Ignavigranum
Microbacterium
Chloroflexia
Thermomicrobiales
JG30_KF_CM45
Paeniglutamicibacter
Paraprevotella
Muribaculaceae
Rikenellaceae_RC9_gut_group
Bifidobacterium_pseudocatenulatum
Thauera
Guggenheimella
Bifidobacterium
Bifidobacteriales
Bifidobacteriaceae
Actinobacteria
Actinobacteria
Desulfomicrobiaceae
Desulfomicrobium
Alloprevotella
Prevotella_2
Prevotella_9
Bacteroidales
Bacteroidia
Bacteroidetes
Prevotellaceae
−4
−2
0
2
4
LDA SCORE (log 10)

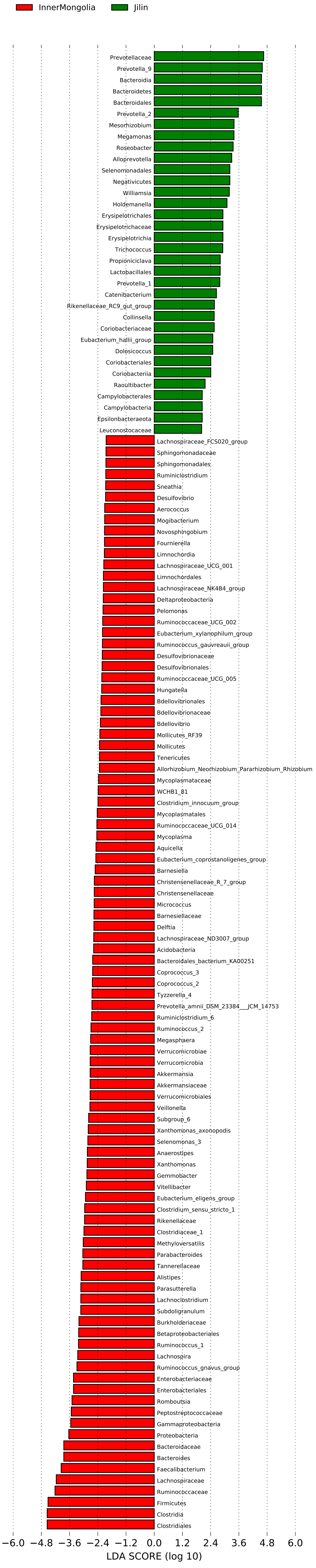
InnerMongolia
Jilin
Prevotellaceae
Prevotella_9
Bacteroidia
Bacteroidetes
Bacteroidales
Prevotella_2
Mesorhizobium
Megamonas
Roseobacter
Alloprevotella
Selenomonadales
Negativicutes
Williamsia
Holdemanella
Erysipelotrichales
Erysipelotrichaceae
Erysipelotrichia
Trichococcus
Propioniciclava
Lactobacillales
Prevotella_1
Catenibacterium
Rikenellaceae_RC9_gut_group
Collinsella
Coriobacteriaceae
Eubacterium_hallii_group
Dolosicoccus
Coriobacteriales
Coriobacteriia
Raoultibacter
Campylobacterales
Campylobacteria
Epsilonbacteraeota
Leuconostocaceae
Lachnospiraceae_FCS020_group
Sphingomonadaceae
Sphingomonadales
Ruminiclostridium
Sneathia
Desulfovibrio
Aerococcus
Mogibacterium
Novosphingobium
Fournierella
Limnochordia
Lachnospiraceae_UCG_001
Limnochordales
Lachnospiraceae_NK4B4_group
Deltaproteobacteria
Pelomonas
Ruminococcaceae_UCG_002
Eubacterium_xylanophilum_group
Ruminococcus_gauvreauii_group
Desulfovibrionaceae
Desulfovibrionales
Ruminococcaceae_UCG_005
Hungatella
Bdellovibrionales
Bdellovibrionaceae
Bdellovibrio
Mollicutes_RF39
Mollicutes
Tenericutes
Allorhizobium_Neorhizobium_Pararhizobium_Rhizobium
Mycoplasmataceae
WCHB1_81
Clostridium_innocuum_group
Mycoplasmatales
Ruminococcaceae_UCG_014
Mycoplasma
Aquicella
Eubacterium_coprostanoligenes_group
Barnesiella
Christensenellaceae_R_7_group
Christensenellaceae
Micrococcus
Barnesiellaceae
Delftia
Lachnospiraceae_ND3007_group
Acidobacteria
Bacteroidales_bacterium_KA00251
Coprococcus_3
Coprococcus_2
Tyzzerella_4
Prevotella_amnii_DSM_23384__JCM_14753
Ruminiclostridium_6
Ruminococcus_2
Megasphaera
Verrucomicrobiae
Verrucomicrobia
Akkermansia
Akkermansiaceae
Verrucomicrobiales
Veillonella
Subgroup_6
Xanthomonas_axonopodis
Selenomonas_3
Anaerostipes
Xanthomonas
Gemmobacter
Vitellibacter
Eubacterium_eligens_group
Clostridium_sensu_stricto_1
Rikenellaceae
Clostridiaceae_1
Methyloversatilis
Parabacteroides
Tannerellaceae
Alistipes
Parasutterella
Lachnoclostridium
Subdoligranulum
Burkholderiaceae
Betaproteobacteriales
Ruminococcus_1
Lachnospira
Ruminococcus_gnavus_group
Enterobacteriaceae
Enterobacteriales
Romboutsia
Peptostreptococcaceae
Gammaproteobacteria
Proteobacteria
Bacteroidaceae
Bacteroides
Faecalibacterium
Lachnospiraceae
Ruminococcaceae
Firmicutes
Clostridia
Clostridiales
−6.0
−4.8
−3.6
−2.4
−1.2
0.0
1.2
2.4
3.6
4.8
6.0
LDA SCORE (log 10)

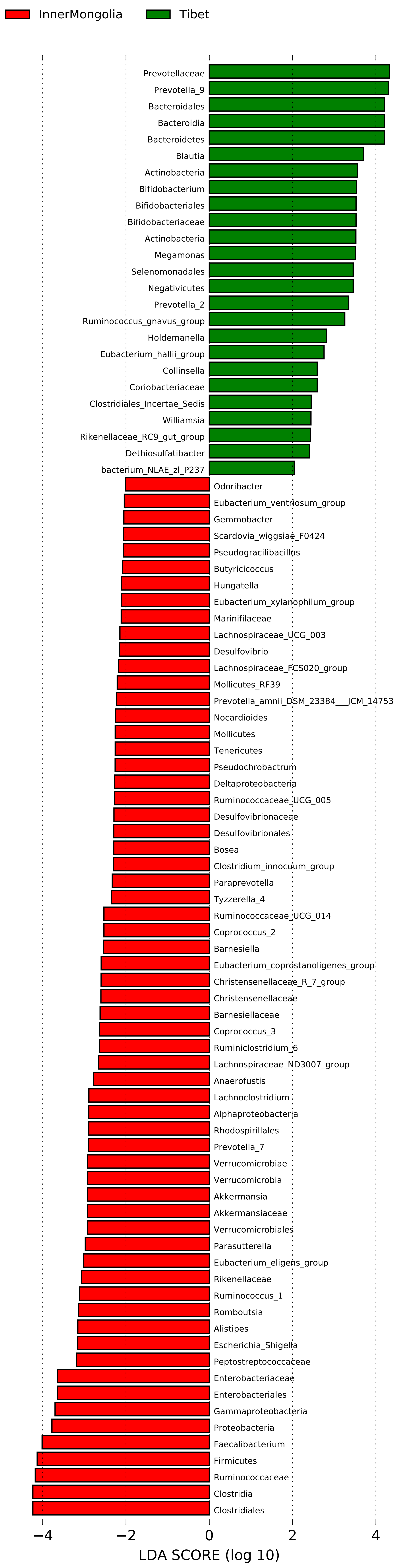

InnerMongolia
Tibet
Prevotellaceae
Prevotella_9
Bacteroidales
Bacteroidia
Bacteroidetes
Blautia
Actinobacteria
Bifidobacterium
Bifidobacteriales
Bifidobacteriaceae
Actinobacteria
Megamonas
Selenomonadales
Negativicutes
Prevotella_2
Ruminococcus_gnavus_group
Holdemanella
Eubacterium_hallii_group
Collinsella
Coriobacteriaceae
Clostridiales_Incertae_Sedis
Williamsia
Rikenellaceae_RC9_gut_group
Dethiosulfatibacter
bacterium_NLAE_zl_P237
Odoribacter
Eubacterium_ventriosum_group
Gemmobacter
Scardovia_wiggsiae_F0424
Pseudogracilibacillus
Butyricicoccus
Hungatella
Eubacterium_xylanophilum_group
Marinifilaceae
Lachnospiraceae_UCG_003
Desulfovibrio
Lachnospiraceae_FCS020_group
Mollicutes_RF39
Prevotella_amnii_DSM_23384__JCM_14753
Nocardioides
Mollicutes
Tenericutes
Pseudochrobactrum
Deltaproteobacteria
Ruminococcaceae_UCG_005
Desulfovibrionaceae
Desulfovibrionales
Bosea
Clostridium_innocuum_group
Paraprevotella
Tyzzerella_4
Ruminococcaceae_UCG_014
Coprococcus_2
Barnesiella
Eubacterium_coprostanoligenes_group
Christensenellaceae_R_7_group
Christensenellaceae
Barnesiellaceae
Coprococcus_3
Ruminiclostridium_6
Lachnospiraceae_ND3007_group
Anaerofustis
Lachnoclostridium
Alphaproteobacteria
Rhodospirillales
Prevotella_7
Verrucomicrobiae
Verrucomicrobia
Akkermansia
Akkermansiaceae
Verrucomicrobiales
Parasutterella
Eubacterium_eligens_group
Rikenellaceae
Ruminococcus_1
Romboutsia
Alistipes
Escherichia_Shigella
Peptostreptococcaceae
Enterobacteriaceae
Enterobacteriales
Gammaproteobacteria
Proteobacteria
Faecalibacterium
Firmicutes
Ruminococcaceae
Clostridia
Clostridiales
−4
−2
0
2
4
LDA SCORE (log 10)

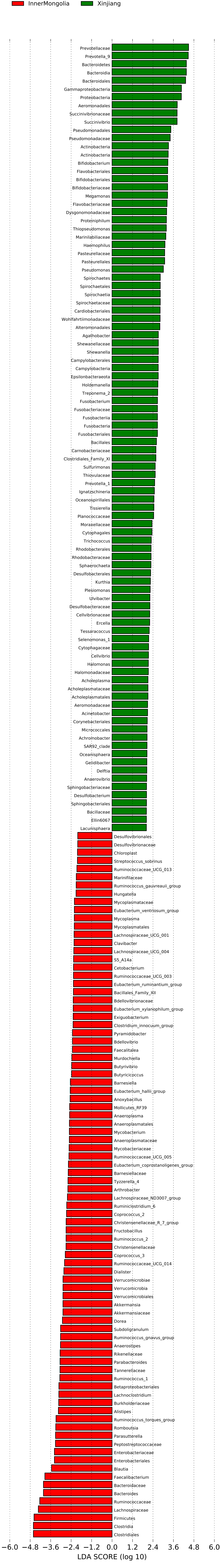
InnerMongolia
Xinjiang
Prevotellaceae
Prevotella_9
Bacteroidetes
Bacteroidia
Bacteroidales
Gammaproteobacteria
Proteobacteria
Aeromonadales
Succinivibrionaceae
Succinivibrio
Pseudomonadales
Pseudomonadaceae
Actinobacteria
Actinobacteria
Bifidobacterium
Flavobacteriales
Bifidobacteriales
Bifidobacteriaceae
Megamonas
Flavobacteriaceae
Dysgonomonadaceae
Proteiniphilum
Thiopseudomonas
Marinilabiliaceae
Haemophilus
Pasteurellaceae
Pasteurellales
Pseudomonas
Spirochaetes
Spirochaetales
Spirochaetia
Spirochaetaceae
Cardiobacteriales
Wohlfahrtiimonadaceae
Alteromonadales
Agathobacter
Shewanellaceae
Shewanella
Campylobacterales
Campylobacteria
Epsilonbacteraeota
Holdemanella
Treponema_2
Fusobacterium
Fusobacteriaceae
Fusobacteriia
Fusobacteria
Fusobacteriales
Bacillales
Carnobacteriaceae
Clostridiales_Family_XI
Sulfurimonas
Thiovulaceae
Prevotella_1
Ignatzschineria
Oceanospirillales
Tissierella
Planococcaceae
Moraxellaceae
Cytophagales
Trichococcus
Rhodobacterales
Rhodobacteraceae
Sphaerochaeta
Desulfobacterales
Kurthia
Plesiomonas
Ulvibacter
Desulfobacteraceae
Cellvibrionaceae
Ercella
Tessaracoccus
Selenomonas_1
Cytophagaceae
Cellvibrio
Halomonas
Halomonadaceae
Acholeplasma
Acholeplasmataceae
Acholeplasmatales
Aeromonadaceae
Acinetobacter
Corynebacteriales
Micrococcales
Achromobacter
SAR92_clade
Oceanisphaera
Gelidibacter
Delftia
Anaerovibrio
Sphingobacteriaceae
Desulfobacterium
Sphingobacteriales
Bacillaceae
Ellin6067
Lacunisphaera
Desulfovibrionales
Desulfovibrionaceae
Chloroplast
Streptococcus_sobrinus
Ruminococcaceae_UCG_013
Marinifilaceae
Ruminococcus_gauvreauii_group
Hungatella
Mycoplasmataceae
Eubacterium_ventriosum_group
Mycoplasma
Mycoplasmatales
Lachnospiraceae_UCG_001
Clavibacter
Lachnospiraceae_UCG_004
S5_A14a
Cetobacterium
Ruminococcaceae_UCG_003
Eubacterium_ruminantium_group
Bacillales_Family_XII
Bdellovibrionaceae
Eubacterium_xylanophilum_group
Exiguobacterium
Clostridium_innocuum_group
Pyramidobacter
Bdellovibrio
Faecalitalea
Murdochiella
Butyrivibrio
Butyricicoccus
Barnesiella
Eubacterium_hallii_group
Anoxybacillus
Mollicutes_RF39
Anaeroplasma
Anaeroplasmatales
Mycobacterium
Anaeroplasmataceae
Mycobacteriaceae
Ruminococcaceae_UCG_005
Eubacterium_coprostanoligenes_group
Barnesiellaceae
Tyzzerella_4
Arthrobacter
Lachnospiraceae_ND3007_group
Ruminiclostridium_6
Coprococcus_2
Christensenellaceae_R_7_group
Fructobacillus
Ruminococcus_2
Christensenellaceae
Coprococcus_3
Ruminococcaceae_UCG_014
Dialister
Verrucomicrobiae
Verrucomicrobia
Verrucomicrobiales
Akkermansia
Akkermansiaceae
Dorea
Subdoligranulum
Ruminococcus_gnavus_group
Anaerostipes
Rikenellaceae
Parabacteroides
Tannerellaceae
Ruminococcus_1
Betaproteobacteriales
Lachnoclostridium
Burkholderiaceae
Alistipes
Ruminococcus_torques_group
Romboutsia
Parasutterella
Peptostreptococcaceae
Enterobacteriaceae
Enterobacteriales
Blautia
Faecalibacterium
Bacteroidaceae
Bacteroides
Ruminococcaceae
Lachnospiraceae
Firmicutes
Clostridia
Clostridiales
−6.0
−4.8
−3.6
−2.4
−1.2
0.0
1.2
2.4
3.6
4.8
6.0
LDA SCORE (log 10)

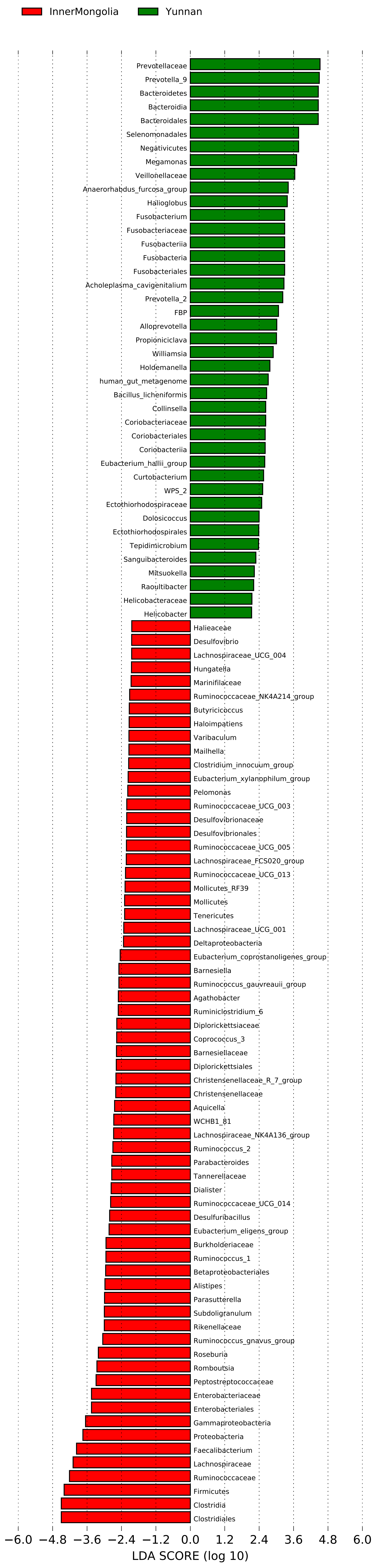
InnerMongolia
Yunnan
Prevotellaceae
Prevotella_9
Bacteroidetes
Bacteroidia
Bacteroidales
Selenomonadales
Negativicutes
Megamonas
Veillonellaceae
Anaerorhabdus_furcosa_group
Halioglobus
Fusobacterium
Fusobacteriaceae
Fusobacteriia
Fusobacteria
Fusobacteriales
Acholeplasma_cavigenitalium
Prevotella_2
FBP
Alloprevotella
Propioniciclava
Williamsia
Holdemanella
human_gut_metagenome
Bacillus_licheniformis
Collinsella
Coriobacteriaceae
Coriobacteriales
Coriobacteriia
Eubacterium_hallii_group
Curtobacterium
WPS_2
Ectothiorhodospiraceae
Dolosicoccus
Ectothiorhodospirales
Tepidimicrobium
Sanguibacteroides
Mitsuokella
Raoultibacter
Helicobacteraceae
Helicobacter
Halieaceae
Desulfovibrio
Lachnospiraceae_UCG_004
Hungatella
Marinifilaceae
Ruminococcaceae_NK4A214_group
Butyricicoccus
Haloimpatiens
Varibaculum
Mailhella
Clostridium_innocuum_group
Eubacterium_xylanophilum_group
Pelomonas
Ruminococcaceae_UCG_003
Desulfovibrionaceae
Desulfovibrionales
Ruminococcaceae_UCG_005
Lachnospiraceae_FCS020_group
Ruminococcaceae_UCG_013
Mollicutes_RF39
Mollicutes
Tenericutes
Lachnospiraceae_UCG_001
Deltaproteobacteria
Eubacterium_coprostanoligenes_group
Barnesiella
Ruminococcus_gauvreauii_group
Agathobacter
Ruminiclostridium_6
Diplorickettsiaceae
Coprococcus_3
Barnesiellaceae
Diplorickettsiales
Christensenellaceae_R_7_group
Christensenellaceae
Aquicella
WCHB1_81
Lachnospiraceae_NK4A136_group
Ruminococcus_2
Parabacteroides
Tannerellaceae
Dialister
Ruminococcaceae_UCG_014
Desulfuribacillus
Eubacterium_eligens_group
Burkholderiaceae
Ruminococcus_1
Betaproteobacteriales
Alistipes
Parasutterella
Subdoligranulum
Rikenellaceae
Ruminococcus_gnavus_group
Roseburia
Romboutsia
Peptostreptococcaceae
Enterobacteriaceae
Enterobacteriales
Gammaproteobacteria
Proteobacteria
Faecalibacterium
Lachnospiraceae
Ruminococcaceae
Firmicutes
Clostridia
Clostridiales
−6.0
−4.8
−3.6
−2.4
−1.2
0.0
1.2
2.4
3.6
4.8
6.0
LDA SCORE (log 10)

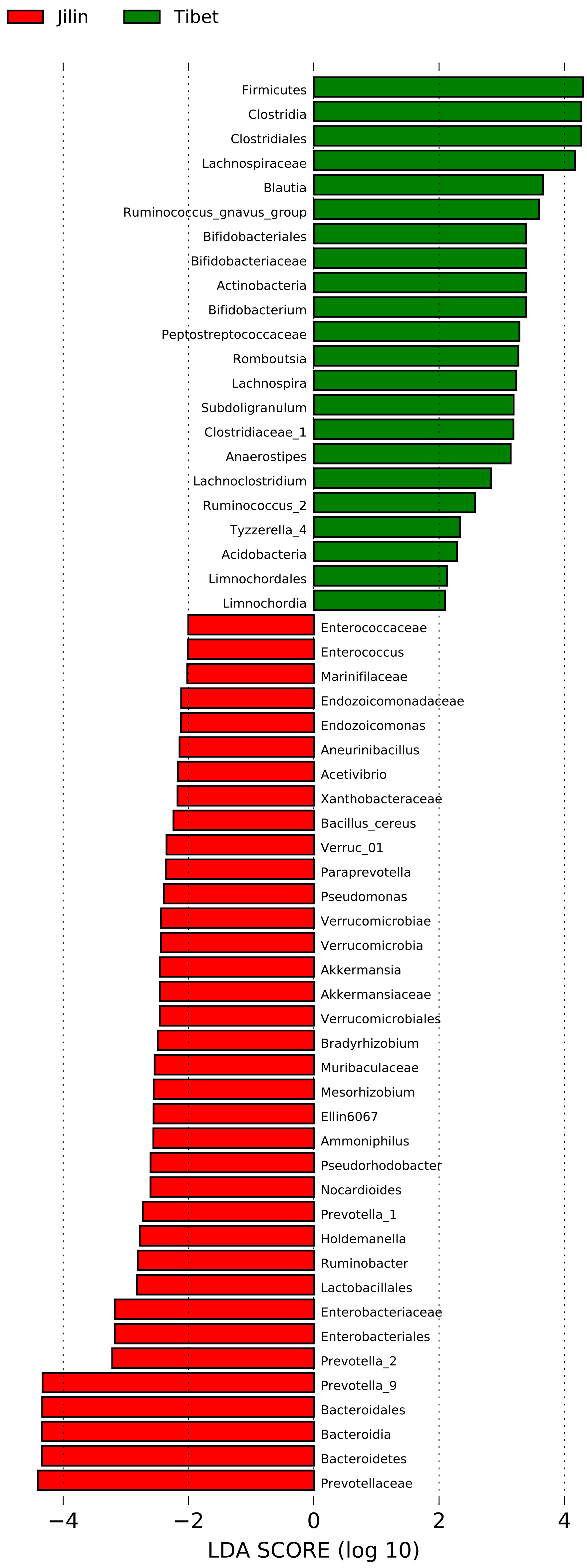
Jilin
Tibet
Firmicutes
Clostridia
Clostridiales
Lachnospiraceae
Blautia
Ruminococcus_gnavus_group
Bifidobacteriales
Bifidobacteriaceae
Actinobacteria
Bifidobacterium
Peptostreptococcaceae
Romboutsia
Lachnospira
Subdoligranulum
Clostridiaceae_1
Anaerostipes
Lachnoclostridium
Ruminococcus_2
Tyzzerella_4
Acidobacteria
Limnochordales
Limnochordia
Enterococcaceae
Enterococcus
Marinifilaceae
Endozoicomonadaceae
Endozoicomonas
Aneurinibacillus
Acetivibrio
Xanthobacteraceae
Bacillus_cereus
Verruc_01
Paraprevotella
Pseudomonas
Verrucomicrobiae
Verrucomicrobia
Akkermansia
Akkermansiaceae
Verrucomicrobiales
Bradyrhizobium
Muribaculaceae
Mesorhizobium
Ellin6067
Ammoniphilus
Pseudorhodobacter
Nocardioides
Prevotella_1
Holdemanella
Ruminobacter
Lactobacillales
Enterobacteriaceae
Enterobacteriales
Prevotella_2
Prevotella_9
Bacteroidales
Bacteroidia
Bacteroidetes
Prevotellaceae
−4
−2
0
2
4
LDA SCORE (log 10)

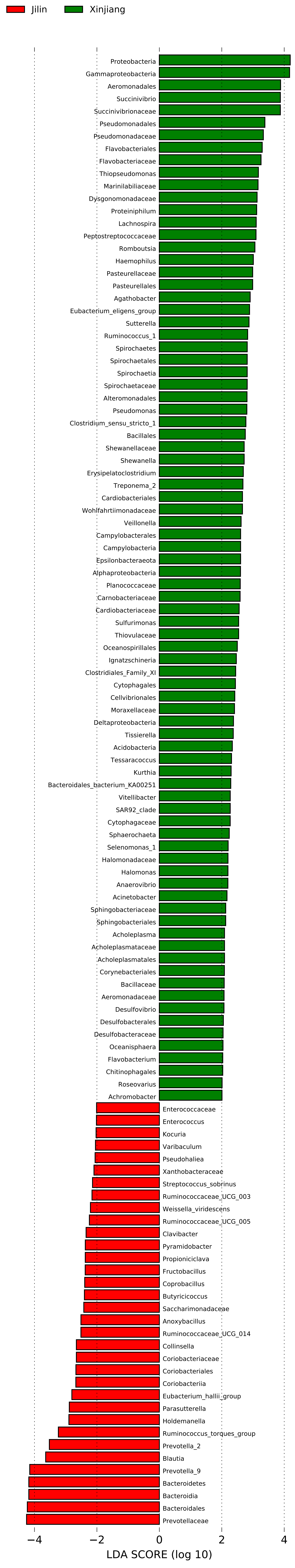

Jilin
Xinjiang
Proteobacteria
Gammaproteobacteria
Aeromonadales
Succinivibrio
Succinivibrionaceae
Pseudomonadales
Pseudomonadaceae
Flavobacteriales
Flavobacteriaceae
Thiopseudomonas
Marinilabiliaceae
Dysgonomonadaceae
Proteiniphilum
Lachnospira
Peptostreptococcaceae
Romboutsia
Haemophilus
Pasteurellaceae
Pasteurellales
Agathobacter
Eubacterium_eligens_group
Sutterella
Ruminococcus_1
Spirochaetes
Spirochaetales
Spirochaetia
Spirochaetaceae
Alteromonadales
Pseudomonas
Clostridium_sensu_stricto_1
Bacillales
Shewanellaceae
Shewanella
Erysipelatoclostridium
Treponema_2
Cardiobacteriales
Wohlfahrtiimonadaceae
Veillonella
Campylobacterales
Campylobacteria
Epsilonbacteraeota
Alphaproteobacteria
Planococcaceae
Carnobacteriaceae
Cardiobacteriaceae
Sulfurimonas
Thiovulaceae
Oceanospirillales
Ignatzschineria
Clostridiales_Family_XI
Cytophagales
Cellvibrionales
Moraxellaceae
Deltaproteobacteria
Tissierella
Acidobacteria
Tessaracoccus
Kurthia
Bacteroidales_bacterium_KA00251
Vitellibacter
SAR92_clade
Cytophagaceae
Sphaerochaeta
Selenomonas_1
Halomonadaceae
Halomonas
Anaerovibrio
Acinetobacter
Sphingobacteriaceae
Sphingobacteriales
Acholeplasma
Acholeplasmataceae
Acholeplasmatales
Corynebacteriales
Bacillaceae
Aeromonadaceae
Desulfovibrio
Desulfobacterales
Desulfobacteraceae
Oceanisphaera
Flavobacterium
Chitinophagales
Roseovarius
Achromobacter
Enterococcaceae
Enterococcus
Kocuria
Varibaculum
Pseudohaliea
Xanthobacteraceae
Streptococcus_sobrinus
Ruminococcaceae_UCG_003
Weissella_viridescens
Ruminococcaceae_UCG_005
Clavibacter
Pyramidobacter
Propioniciclava
Fructobacillus
Coprobacillus
Butyricicoccus
Saccharimonadaceae
Anoxybacillus
Ruminococcaceae_UCG_014
Collinsella
Coriobacteriaceae
Coriobacteriales
Coriobacteriia
Eubacterium_hallii_group
Parasutterella
Holdemanella
Ruminococcus_torques_group
Prevotella_2
Blautia
Prevotella_9
Bacteroidetes
Bacteroidia
Bacteroidales
Prevotellaceae
−4
−2
0
2
4
LDA SCORE (log 10)

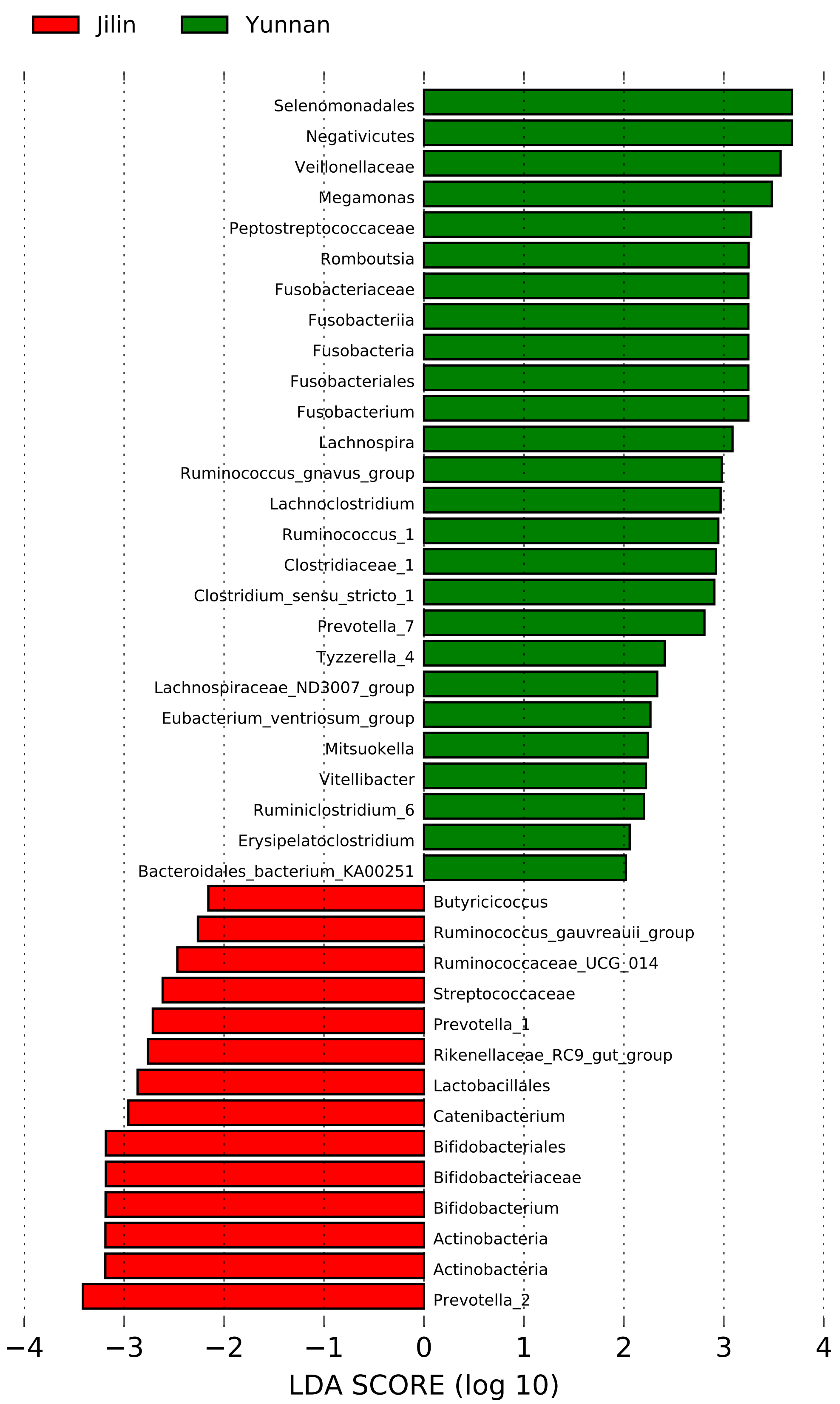

Jilin
Yunnan
Selenomonadales
Negativicutes
Veillonellaceae
Megamonas
Peptostreptococcaceae
Romboutsia
Fusobacteriaceae
Fusobacteriia
Fusobacteria
Fusobacteriales
Fusobacterium
Lachnospira
Ruminococcus_gnavus_group
Lachnoclostridium
Ruminococcus_1
Clostridiaceae_1
Clostridium_sensu_stricto_1
Prevotella_7
Tyzzerella_4
Lachnospiraceae_ND3007_group
Eubacterium_ventriosum_group
Mitsuokella
Vitellibacter
Ruminiclostridium_6
Erysipelatoclostridium
Bacteroidales_bacterium_KA00251
Butyricicoccus
Ruminococcus_gauvreauii_group
Ruminococcaceae_UCG_014
Streptococcaceae
Prevotella_1
Rikenellaceae_RC9_gut_group
Lactobacillales
Catenibacterium
Bifidobacteriales
Bifidobacteriaceae
Bifidobacterium
Actinobacteria
Actinobacteria
Prevotella_2
−4
−3
−2
−1
0
1
2
3
4
LDA SCORE (log 10)

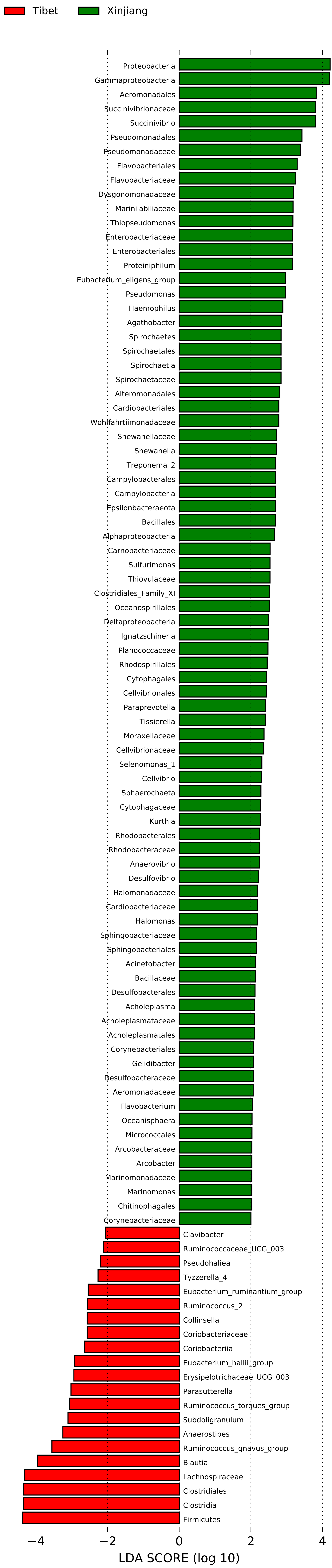

Tibet
Xinjiang
Proteobacteria
Gammaproteobacteria
Aeromonadales
Succinivibrionaceae
Succinivibrio
Pseudomonadales
Pseudomonadaceae
Flavobacteriales
Flavobacteriaceae
Dysgonomonadaceae
Marinilabiliaceae
Thiopseudomonas
Enterobacteriaceae
Enterobacteriales
Proteiniphilum
Eubacterium_eligens_group
Pseudomonas
Haemophilus
Agathobacter
Spirochaetes
Spirochaetales
Spirochaetia
Spirochaetaceae
Alteromonadales
Cardiobacteriales
Wohlfahrtiimonadaceae
Shewanellaceae
Shewanella
Treponema_2
Campylobacterales
Campylobacteria
Epsilonbacteraeota
Bacillales
Alphaproteobacteria
Carnobacteriaceae
Sulfurimonas
Thiovulaceae
Clostridiales_Family_XI
Oceanospirillales
Deltaproteobacteria
Ignatzschineria
Planococcaceae
Rhodospirillales
Cytophagales
Cellvibrionales
Paraprevotella
Tissierella
Moraxellaceae
Cellvibrionaceae
Selenomonas_1
Cellvibrio
Sphaerochaeta
Cytophagaceae
Kurthia
Rhodobacterales
Rhodobacteraceae
Anaerovibrio
Desulfovibrio
Halomonadaceae
Cardiobacteriaceae
Halomonas
Sphingobacteriaceae
Sphingobacteriales
Acinetobacter
Bacillaceae
Desulfobacterales
Acholeplasma
Acholeplasmataceae
Acholeplasmatales
Corynebacteriales
Gelidibacter
Desulfobacteraceae
Aeromonadaceae
Flavobacterium
Oceanisphaera
Micrococcales
Arcobacteraceae
Arcobacter
Marinomonadaceae
Marinomonas
Chitinophagales
Corynebacteriaceae
Clavibacter
Ruminococcaceae_UCG_003
Pseudohaliea
Tyzzerella_4
Eubacterium_ruminantium_group
Ruminococcus_2
Collinsella
Coriobacteriaceae
Coriobacteriia
Eubacterium_hallii_group
Erysipelotrichaceae_UCG_003
Parasutterella
Ruminococcus_torques_group
Subdoligranulum
Anaerostipes
Ruminococcus_gnavus_group
Blautia
Lachnospiraceae
Clostridiales
Clostridia
Firmicutes
−4
−2
0
2
4
LDA SCORE (log 10)

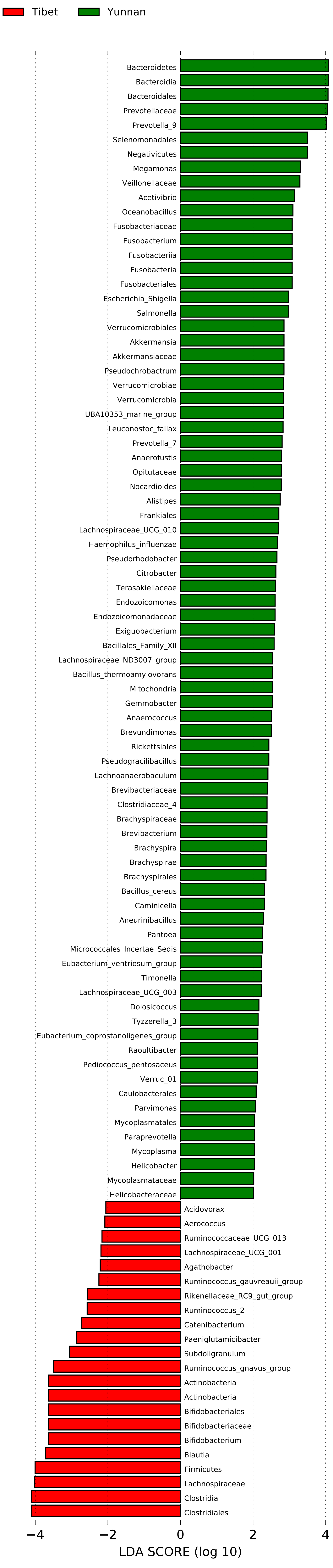

Tibet
Yunnan
Bacteroidetes
Bacteroidia
Bacteroidales
Prevotellaceae
Prevotella_9
Selenomonadales
Negativicutes
Megamonas
Veillonellaceae
Acetivibrio
Oceanobacillus
Fusobacteriaceae
Fusobacterium
Fusobacteriia
Fusobacteria
Fusobacteriales
Escherichia_Shigella
Salmonella
Verrucomicrobiales
Akkermansia
Akkermansiaceae
Pseudochrobactrum
Verrucomicrobiae
Verrucomicrobia
UBA10353_marine_group
Leuconostoc_fallax
Prevotella_7
Anaerofustis
Opitutaceae
Nocardioides
Alistipes
Frankiales
Lachnospiraceae_UCG_010
Haemophilus_influenzae
Pseudorhodobacter
Citrobacter
Terasakiellaceae
Endozoicomonas
Endozoicomonadaceae
Exiguobacterium
Bacillales_Family_XII
Lachnospiraceae_ND3007_group
Bacillus_thermoamylovorans
Mitochondria
Gemmobacter
Anaerococcus
Brevundimonas
Rickettsiales
Pseudogracilibacillus
Lachnoanaerobaculum
Brevibacteriaceae
Clostridiaceae_4
Brachyspiraceae
Brevibacterium
Brachyspira
Brachyspirae
Brachyspirales
Bacillus_cereus
Caminicella
Aneurinibacillus
Pantoea
Micrococcales_Incertae_Sedis
Eubacterium_ventriosum_group
Timonella
Lachnospiraceae_UCG_003
Dolosicoccus
Tyzzerella_3
Eubacterium_coprostanoligenes_group
Raoultibacter
Pediococcus_pentosaceus
Verruc_01
Caulobacterales
Parvimonas
Mycoplasmatales
Paraprevotella
Mycoplasma
Helicobacter
Mycoplasmataceae
Helicobacteraceae
Acidovorax
Aerococcus
Ruminococcaceae_UCG_013
Lachnospiraceae_UCG_001
Agathobacter
Ruminococcus_gauvreauii_group
Rikenellaceae_RC9_gut_group
Ruminococcus_2
Catenibacterium
Paeniglutamicibacter
Subdoligranulum
Ruminococcus_gnavus_group
Actinobacteria
Actinobacteria
Bifidobacteriales
Bifidobacteriaceae
Bifidobacterium
Blautia
Firmicutes
Lachnospiraceae
Clostridia
Clostridiales
−4
−2
0
2
4
LDA SCORE (log 10)

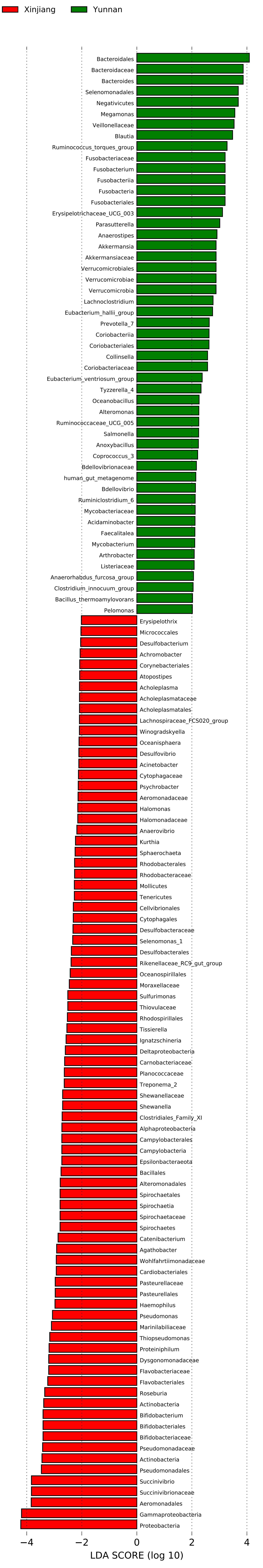

Xinjiang
Yunnan
Bacteroidales
Bacteroidaceae
Bacteroides
Selenomonadales
Negativicutes
Megamonas
Veillonellaceae
Blautia
Ruminococcus_torques_group
Fusobacteriaceae
Fusobacterium
Fusobacteriia
Fusobacteria
Fusobacteriales
Erysipelotrichaceae_UCG_003
Parasutterella
Anaerostipes
Akkermansia
Akkermansiaceae
Verrucomicrobiales
Verrucomicrobiae
Verrucomicrobia
Lachnoclostridium
Eubacterium_hallii_group
Prevotella_7
Coriobacteriia
Coriobacteriales
Collinsella
Coriobacteriaceae
Eubacterium_ventriosum_group
Tyzzerella_4
Oceanobacillus
Alteromonas
Ruminococcaceae_UCG_005
Salmonella
Anoxybacillus
Coprococcus_3
Bdellovibrionaceae
human_gut_metagenome
Bdellovibrio
Ruminiclostridium_6
Mycobacteriaceae
Acidaminobacter
Faecalitalea
Mycobacterium
Arthrobacter
Listeriaceae
Anaerorhabdus_furcosa_group
Clostridium_innocuum_group
Bacillus_thermoamylovorans
Pelomonas
Erysipelothrix
Micrococcales
Desulfobacterium
Achromobacter
Corynebacteriales
Atopostipes
Acholeplasma
Acholeplasmataceae
Acholeplasmatales
Lachnospiraceae_FCS020_group
Winogradskyella
Oceanisphaera
Desulfovibrio
Acinetobacter
Cytophagaceae
Psychrobacter
Aeromonadaceae
Halomonas
Halomonadaceae
Anaerovibrio
Kurthia
Sphaerochaeta
Rhodobacterales
Rhodobacteraceae
Mollicutes
Tenericutes
Cellvibrionales
Cytophagales
Desulfobacteraceae
Selenomonas_1
Desulfobacterales
Rikenellaceae_RC9_gut_group
Oceanospirillales
Moraxellaceae
Sulfurimonas
Thiovulaceae
Rhodospirillales
Tissierella
Ignatzschineria
Deltaproteobacteria
Carnobacteriaceae
Planococcaceae
Treponema_2
Shewanellaceae
Shewanella
Clostridiales_Family_XI
Alphaproteobacteria
Campylobacterales
Campylobacteria
Epsilonbacteraeota
Bacillales
Alteromonadales
Spirochaetales
Spirochaetia
Spirochaetaceae
Spirochaetes
Catenibacterium
Agathobacter
Wohlfahrtiimonadaceae
Cardiobacteriales
Pasteurellaceae
Pasteurellales
Haemophilus
Pseudomonas
Marinilabiliaceae
Thiopseudomonas
Proteiniphilum
Dysgonomonadaceae
Flavobacteriaceae
Flavobacteriales
Roseburia
Actinobacteria
Bifidobacterium
Bifidobacteriales
Bifidobacteriaceae
Pseudomonadaceae
Actinobacteria
Pseudomonadales
Succinivibrio
Succinivibrionaceae
Aeromonadales
Gammaproteobacteria
Proteobacteria
−4
−2
0
2
4
LDA SCORE (log 10)

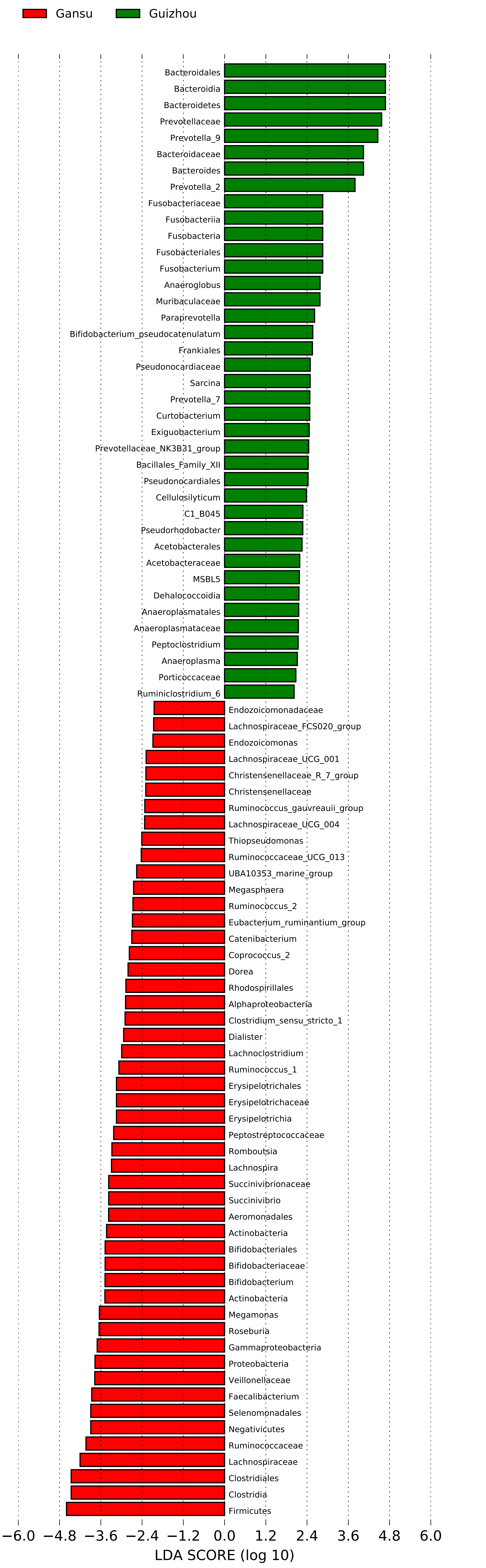
Gansu
Guizhou
Bacteroidales
Bacteroidia
Bacteroidetes
Prevotellaceae
Prevotella_9
Bacteroidaceae
Bacteroides
Prevotella_2
Fusobacteriaceae
Fusobacteriia
Fusobacteria
Fusobacteriales
Fusobacterium
Anaeroglobus
Muribaculaceae
Paraprevotella
Bifidobacterium_pseudocatenulatum
Frankiales
Pseudonocardiaceae
Sarcina
Prevotella_7
Curtobacterium
Exiguobacterium
Prevotellaceae_NK3B31_group
Bacillales_Family_XII
Pseudonocardiales
Cellulosilyticum
C1_B045
Pseudorhodobacter
Acetobacterales
Acetobacteraceae
MSBL5
Dehalococcoidia
Anaeroplasmatales
Anaeroplasmataceae
Peptoclostridium
Anaeroplasma
Porticoccaceae
Ruminiclostridium_6
Endozoicomonadaceae
Lachnospiraceae_FCS020_group
Endozoicomonas
Lachnospiraceae_UCG_001
Christensenellaceae_R_7_group
Christensenellaceae
Ruminococcus_gauvreauii_group
Lachnospiraceae_UCG_004
Thiopseudomonas
Ruminococcaceae_UCG_013
UBA10353_marine_group
Megasphaera
Ruminococcus_2
Eubacterium_ruminantium_group
Catenibacterium
Coprococcus_2
Dorea
Rhodospirillales
Alphaproteobacteria
Clostridium_sensu_stricto_1
Dialister
Lachnoclostridium
Ruminococcus_1
Erysipelotrichales
Erysipelotrichaceae
Erysipelotrichia
Peptostreptococcaceae
Romboutsia
Lachnospira
Succinivibrionaceae
Succinivibrio
Aeromonadales
Actinobacteria
Bifidobacteriales
Bifidobacteriaceae
Bifidobacterium
Actinobacteria
Megamonas
Roseburia
Gammaproteobacteria
Proteobacteria
Veillonellaceae
Faecalibacterium
Selenomonadales
Negativicutes
Ruminococcaceae
Lachnospiraceae
Clostridiales
Clostridia
Firmicutes
−6.0 −4.8 −3.6 −2.4 −1.2 0.0 1.2 2.4 3.6 4.8 6.0
LDA SCORE (log 10)

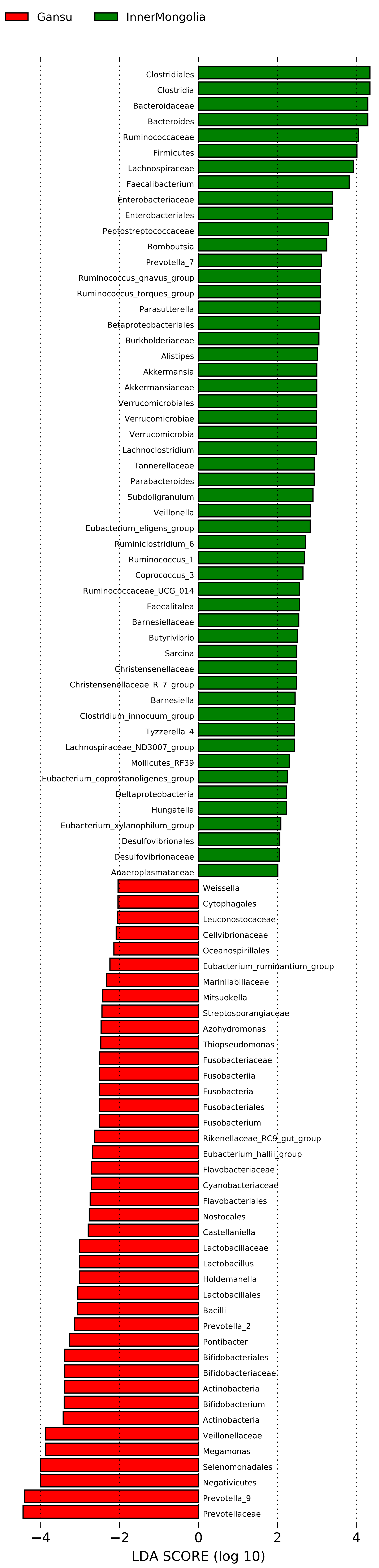
Gansu
InnerMongolia
Clostridiales
Clostridia
Bacteroidaceae
Bacteroides
Ruminococcaceae
Firmicutes
Lachnospiraceae
Faecalibacterium
Enterobacteriaceae
Enterobacteriales
Peptostreptococcaceae
Romboutsia
Prevotella_7
Ruminococcus_gnavus_group
Ruminococcus_torques_group
Parasutterella
Betaproteobacteriales
Burkholderiaceae
Alistipes
Akkermansia
Akkermansiaceae
Verrucomicrobiales
Verrucomicrobiae
Verrucomicrobia
Lachnoclostridium
Tannerellaceae
Parabacteroides
Subdoligranulum
Veillonella
Eubacterium_eligens_group
Ruminiclostridium_6
Ruminococcus_1
Coprococcus_3
Ruminococcaceae_UCG_014
Faecalitalea
Barnesiellaceae
Butyrivibrio
Sarcina
Christensenellaceae
Christensenellaceae_R_7_group
Barnesiella
Clostridium_innocuum_group
Tyzzerella_4
Lachnospiraceae_ND3007_group
Mollicutes_RF39
Eubacterium_coprostanoligenes_group
Deltaproteobacteria
Hungatella
Eubacterium_xylanophilum_group
Desulfovibrionales
Desulfovibrionaceae
Anaeroplasmataceae
Weissella
Cytophagales
Leuconostocaceae
Cellvibrionaceae
Oceanospirillales
Eubacterium_ruminantium_group
Marinilabiliaceae
Mitsuokella
Streptosporangiaceae
Azohydromonas
Thiopseudomonas
Fusobacteriaceae
Fusobacteriia
Fusobacteria
Fusobacteriales
Fusobacterium
Rikenellaceae_RC9_gut_group
Eubacterium_hallii_group
Flavobacteriaceae
Cyanobacteriaceae
Flavobacteriales
Nostocales
Castellaniella
Lactobacillaceae
Lactobacillus
Holdemanella
Lactobacillales
Bacilli
Prevotella_2
Pontibacter
Bifidobacteriales
Bifidobacteriaceae
Actinobacteria
Bifidobacterium
Actinobacteria
Veillonellaceae
Megamonas
Selenomonadales
Negativicutes
Prevotella_9
Prevotellaceae
−4
−2
0
2
4
LDA SCORE (log 10)

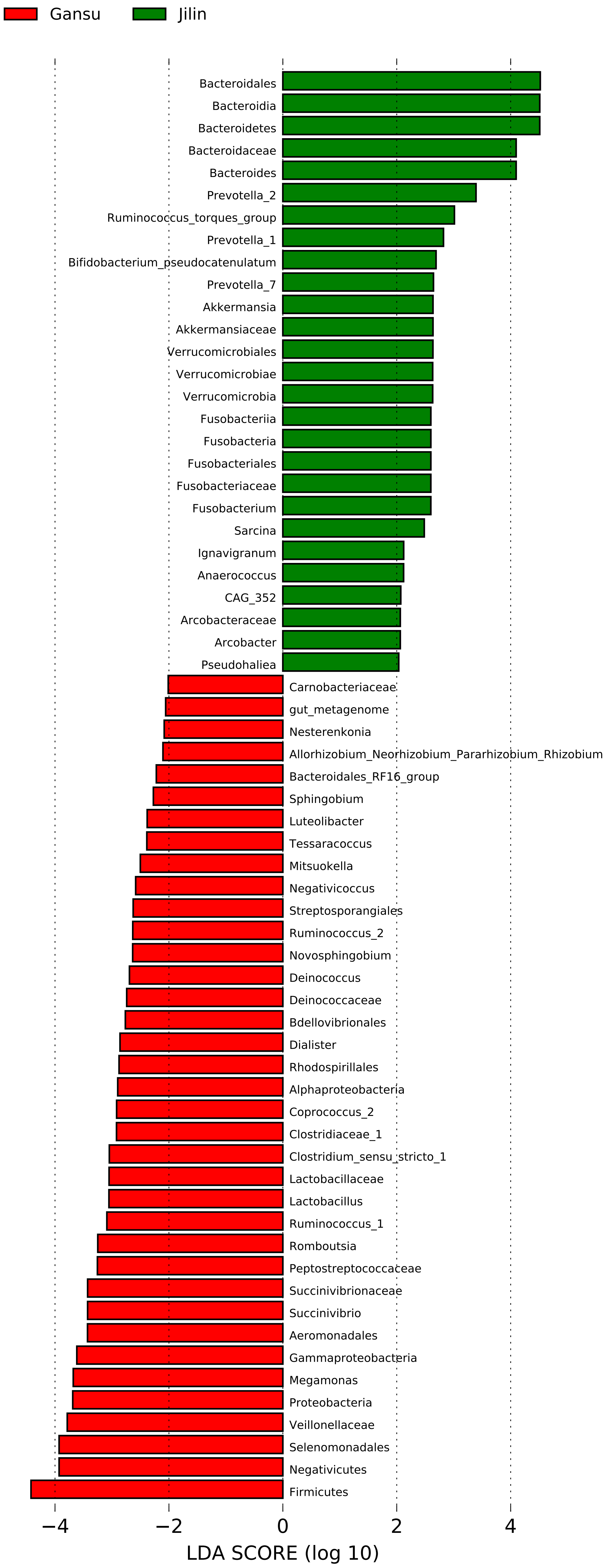
Gansu
Jilin
Bacteroidales
Bacteroidia
Bacteroidetes
Bacteroidaceae
Bacteroides
Prevotella_2
Ruminococcus_torques_group
Prevotella_1
Bifidobacterium_pseudocatenulatum
Prevotella_7
Akkermansia
Akkermansiaceae
Verrucomicrobiales
Verrucomicrobiae
Verrucomicrobia
Fusobacteriia
Fusobacteria
Fusobacteriales
Fusobacteriaceae
Fusobacterium
Sarcina
Ignavigranum
Anaerococcus
CAG_352
Arcobacteraceae
Arcobacter
Pseudohaliea
Carnobacteriaceae
gut_metagenome
Nesterenkonia
Allorhizobium_Neorhizobium_Pararhizobium_Rhizobium
Bacteroidales_RF16_group
Sphingobium
Luteolibacter
Tessaracoccus
Mitsuokella
Negativicoccus
Streptosporangiales
Ruminococcus_2
Novosphingobium
Deinococcus
Deinococcaceae
Bdellovibrionales
Dialister
Rhodospirillales
Alphaproteobacteria
Coprococcus_2
Clostridiaceae_1
Clostridium_sensu_stricto_1
Lactobacillaceae
Lactobacillus
Ruminococcus_1
Romboutsia
Peptostreptococcaceae
Succinivibrionaceae
Succinivibrio
Aeromonadales
Gammaproteobacteria
Megamonas
Proteobacteria
Veillonellaceae
Selenomonadales
Negativicutes
Firmicutes
−4
−2
0
2
4
LDA SCORE (log 10)

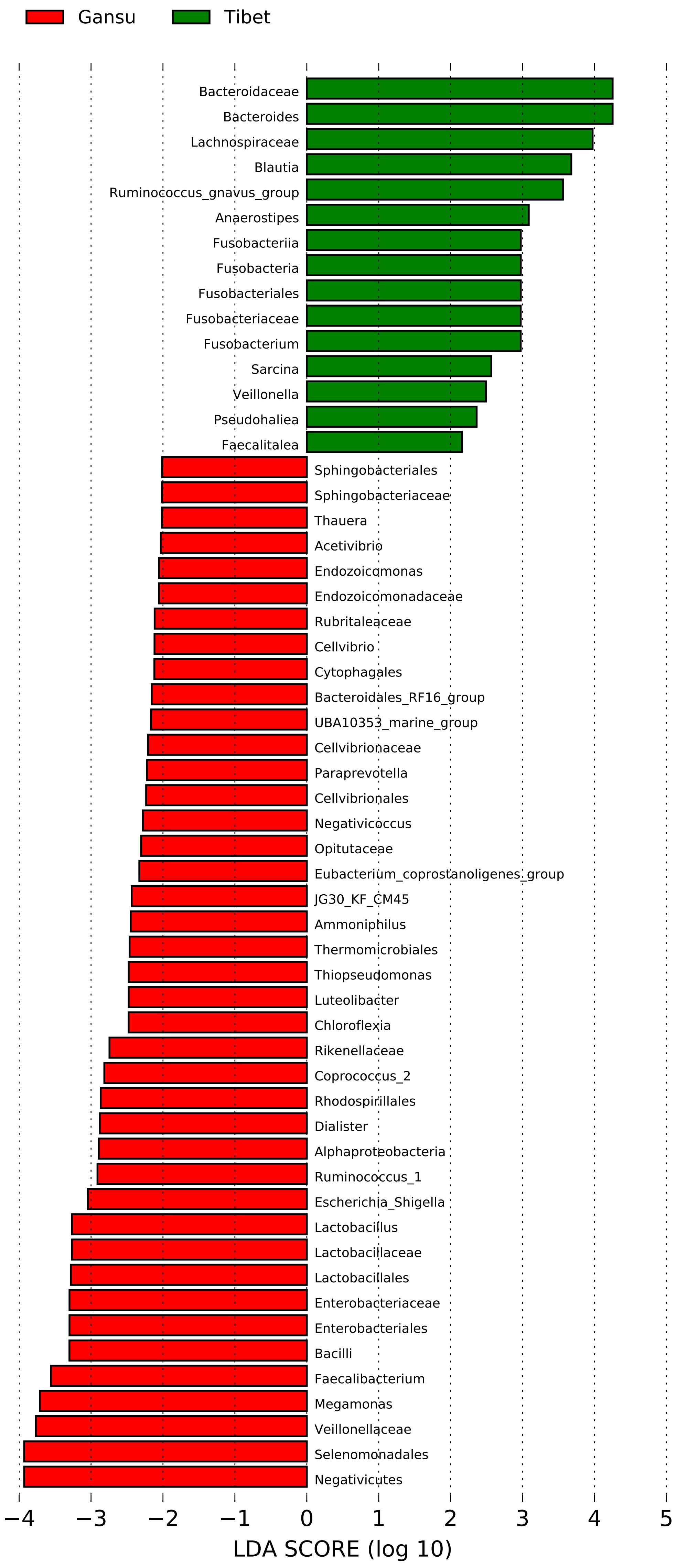
Gansu
Tibet
Bacteroidaceae
Bacteroides
Lachnospiraceae
Blautia
Ruminococcus_gnavus_group
Anaerostipes
Fusobacteriia
Fusobacteria
Fusobacteriales
Fusobacteriaceae
Fusobacterium
Sarcina
Veillonella
Pseudohaliea
Faecalitalea
Sphingobacteriales
Sphingobacteriaceae
Thauera
Acetivibrio
Endozoicomonas
Endozoicomonadaceae
Rubritaleaceae
Cellvibrio
Cytophagales
Bacteroidales_RF16_group
UBA10353_marine_group
Cellvibrionaceae
Paraprevotella
Cellvibrionales
Negativicoccus
Opitutaceae
Eubacterium_coprostanoligenes_group
JG30_KF_CM45
Ammoniphilus
Thermomicrobiales
Thiopseudomonas
Luteolibacter
Chloroflexia
Rikenellaceae
Coprococcus_2
Rhodospirillales
Dialister
Alphaproteobacteria
Ruminococcus_1
Escherichia_Shigella
Lactobacillus
Lactobacillaceae
Lactobacillales
Enterobacteriaceae
Enterobacteriales
Bacilli
Faecalibacterium
Megamonas
Veillonellaceae
Selenomonadales
Negativicutes
−4
−3
−2
−1
0
1
2
3
4
5
LDA SCORE (log 10)

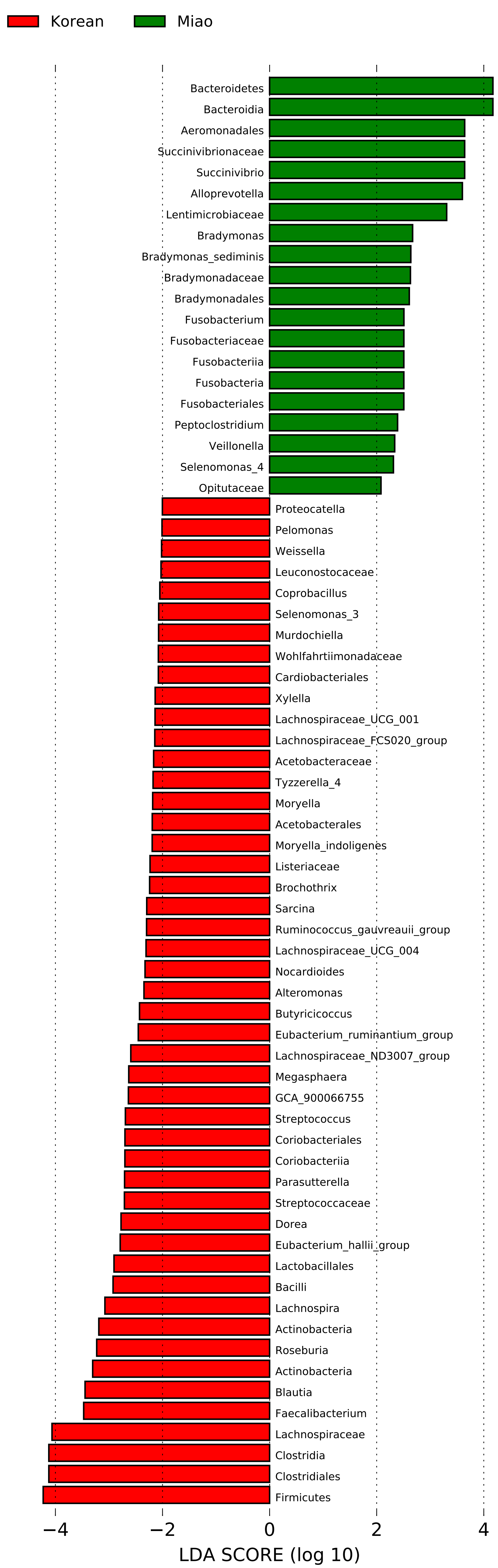
Korean
Miao
Bacteroidetes
Bacteroidia
Aeromonadales
Succinivibrionaceae
Succinivibrio
Alloprevotella
Lentimicrobiaceae
Bradymonas
Bradymonas_sediminis
Bradymonadaceae
Bradymonadales
Fusobacterium
Fusobacteriaceae
Fusobacteriia
Fusobacteria
Fusobacteriales
Peptoclostridium
Veillonella
Selenomonas_4
Opitutaceae
Proteocatella
Pelomonas
Weissella
Leuconostocaceae
Coprobacillus
Selenomonas_3
Murdochiella
Wohlfahrtiimonadaceae
Cardiobacteriales
Xylella
Lachnospiraceae_UCG_001
Lachnospiraceae_FCS020_group
Acetobacteraceae
Tyzzerella_4
Moryella
Acetobacterales
Moryella_indoligenes
Listeriaceae
Brochothrix
Sarcina
Ruminococcus_gauvreauii_group
Lachnospiraceae_UCG_004
Nocardioides
Alteromonas
Butyricicoccus
Eubacterium_ruminantium_group
Lachnospiraceae_ND3007_group
Megasphaera
GCA_900066755
Streptococcus
Coriobacteriales
Coriobacteriia
Parasutterella
Streptococcaceae
Dorea
Eubacterium_hallii_group
Lactobacillales
Bacilli
Lachnospira
Actinobacteria
Roseburia
Actinobacteria
Blautia
Faecalibacterium
Lachnospiraceae
Clostridia
Clostridiales
Firmicutes
−4
−2
0
2
4
LDA SCORE (log 10)

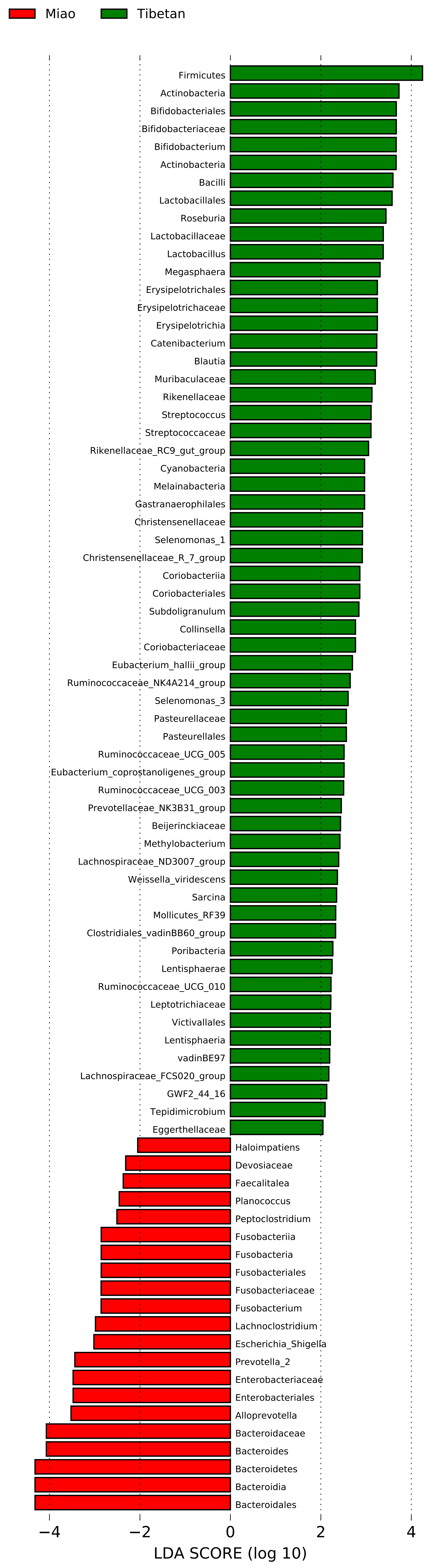

Miao
Tibetan
Firmicutes
Actinobacteria
Bifidobacteriales
Bifidobacteriaceae
Bifidobacterium
Actinobacteria
Bacilli
Lactobacillales
Roseburia
Lactobacillaceae
Lactobacillus
Megasphaera
Erysipelotrichales
Erysipelotrichaceae
Erysipelotrichia
Catenibacterium
Blautia
Muribaculaceae
Rikenellaceae
Streptococcus
Streptococcaceae
Rikenellaceae_RC9_gut_group
Cyanobacteria
Melainabacteria
Gastranaerophilales
Christensenellaceae
Selenomonas_1
Christensenellaceae_R_7_group
Coriobacteriia
Coriobacteriales
Subdoligranulum
Collinsella
Coriobacteriaceae
Eubacterium_hallii_group
Ruminococcaceae_NK4A214_group
Selenomonas_3
Pasteurellaceae
Pasteurellales
Ruminococcaceae_UCG_005
Eubacterium_coprostanoligenes_group
Ruminococcaceae_UCG_003
Prevotellaceae_NK3B31_group
Beijerinckiaceae
Methylobacterium
Lachnospiraceae_ND3007_group
Weissella_viridescens
Sarcina
Mollicutes_RF39
Clostridiales_vadinBB60_group
Poribacteria
Lentisphaerae
Ruminococcaceae_UCG_010
Leptotrichiaceae
Victivallales
Lentisphaeria
vadinBE97
Lachnospiraceae_FCS020_group
GWF2_44_16
Tepidimicrobium
Eggerthellaceae
Haloimpatiens
Devosiaceae
Faecalitalea
Planococcus
Peptoclostridium
Fusobacteriia
Fusobacteria
Fusobacteriales
Fusobacteriaceae
Fusobacterium
Lachnoclostridium
Escherichia_Shigella
Prevotella_2
Enterobacteriaceae
Enterobacteriales
Alloprevotella
Bacteroidaceae
Bacteroides
Bacteroidetes
Bacteroidia
Bacteroidales
−4
−2
0
2
4
LDA SCORE (log 10)

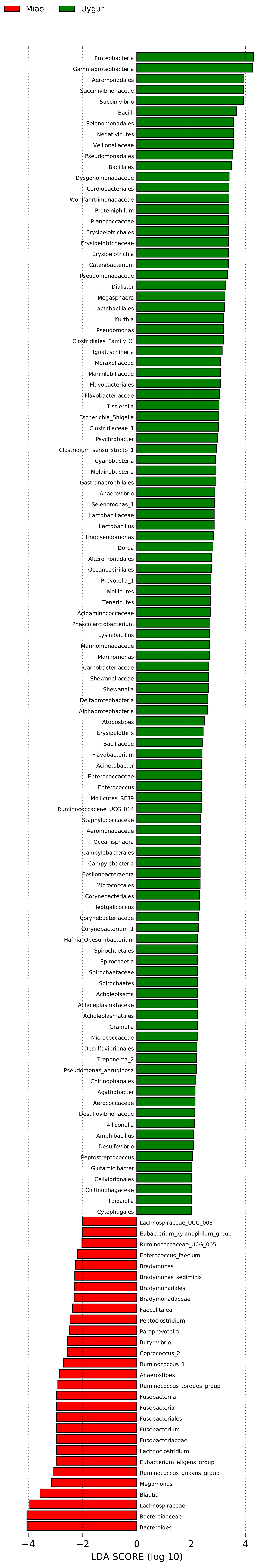
Miao
Uygur
Proteobacteria
Gammaproteobacteria
Aeromonadales
Succinivibrionaceae
Succinivibrio
Bacilli
Selenomonadales
Negativicutes
Veillonellaceae
Pseudomonadales
Bacillales
Dysgonomonadaceae
Cardiobacteriales
Wohlfahrtiimonadaceae
Proteiniphilum
Planococcaceae
Erysipelotrichales
Erysipelotrichaceae
Erysipelotrichia
Catenibacterium
Pseudomonadaceae
Dialister
Megasphaera
Lactobacillales
Kurthia
Pseudomonas
Clostridiales_Family_XI
Ignatzschineria
Moraxellaceae
Marinilabiliaceae
Flavobacteriales
Flavobacteriaceae
Tissierella
Escherichia_Shigella
Clostridiaceae_1
Psychrobacter
Clostridium_sensu_stricto_1
Cyanobacteria
Melainabacteria
Gastranaerophilales
Anaerovibrio
Selenomonas_1
Lactobacillaceae
Lactobacillus
Thiopseudomonas
Dorea
Alteromonadales
Oceanospirillales
Prevotella_1
Mollicutes
Tenericutes
Acidaminococcaceae
Phascolarctobacterium
Lysinibacillus
Marinomonadaceae
Marinomonas
Carnobacteriaceae
Shewanellaceae
Shewanella
Deltaproteobacteria
Alphaproteobacteria
Atopostipes
Erysipelothrix
Bacillaceae
Flavobacterium
Acinetobacter
Enterococcaceae
Enterococcus
Mollicutes_RF39
Ruminococcaceae_UCG_014
Staphylococcaceae
Aeromonadaceae
Oceanisphaera
Campylobacterales
Campylobacteria
Epsilonbacteraeota
Micrococcales
Corynebacteriales
Jeotgalicoccus
Corynebacteriaceae
Corynebacterium_1
Hafnia_Obesumbacterium
Spirochaetales
Spirochaetia
Spirochaetaceae
Spirochaetes
Acholeplasma
Acholeplasmataceae
Acholeplasmatales
Gramella
Micrococcaceae
Desulfovibrionales
Treponema_2
Pseudomonas_aeruginosa
Chitinophagales
Agathobacter
Aerococcaceae
Desulfovibrionaceae
Allisonella
Amphibacillus
Desulfovibrio
Peptostreptococcus
Glutamicibacter
Cellvibrionales
Chitinophagaceae
Taibaiella
Cytophagales
Lachnospiraceae_UCG_003
Eubacterium_xylanophilum_group
Ruminococcaceae_UCG_005
Enterococcus_faecium
Bradymonas
Bradymonas_sediminis
Bradymonadales
Bradymonadaceae
Faecalitalea
Peptoclostridium
Paraprevotella
Butyrivibrio
Coprococcus_2
Ruminococcus_1
Anaerostipes
Ruminococcus_torques_group
Fusobacteriia
Fusobacteria
Fusobacteriales
Fusobacterium
Fusobacteriaceae
Lachnoclostridium
Eubacterium_eligens_group
Ruminococcus_gnavus_group
Megamonas
Blautia
Lachnospiraceae
Bacteroidaceae
Bacteroides
−4
−2
0
2
4
LDA SCORE (log 10)

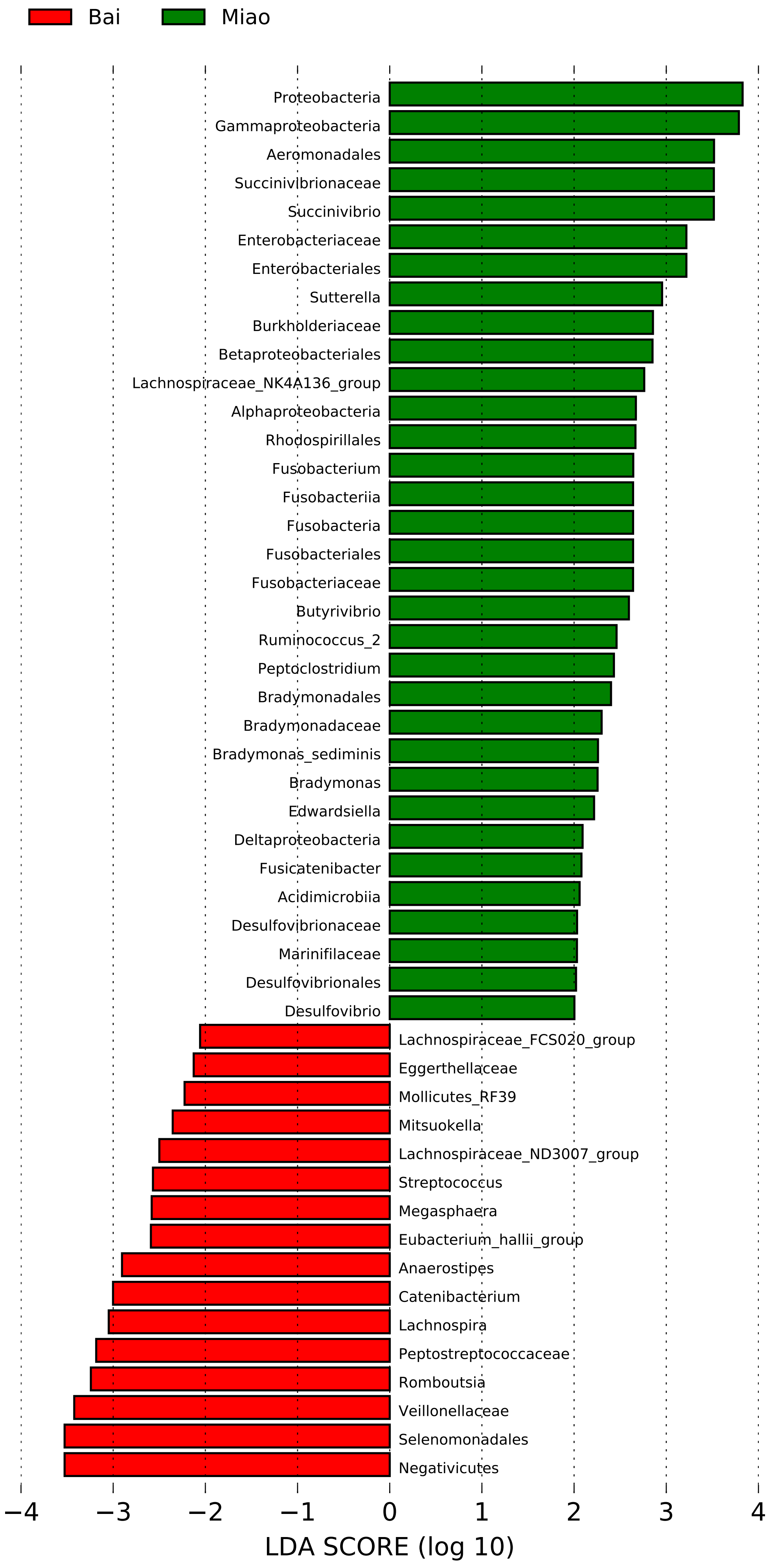

Bai
Miao
Proteobacteria
Gammaproteobacteria
Aeromonadales
Succinivibrionaceae
Succinivibrio
Enterobacteriaceae
Enterobacteriales
Sutterella
Burkholderiaceae
Betaproteobacteriales
Lachnospiraceae_NK4A136_group
Alphaproteobacteria
Rhodospirillales
Fusobacterium
Fusobacteriia
Fusobacteria
Fusobacteriales
Fusobacteriaceae
Butyrivibrio
Ruminococcus_2
Peptoclostridium
Bradymonadales
Bradymonadaceae
Bradymonas_sediminis
Bradymonas
Edwardsiella
Deltaproteobacteria
Fusicatenibacter
Acidimicrobiia
Desulfovibrionaceae
Marinifilaceae
Desulfovibrionales
Desulfovibrio
Lachnospiraceae_FCS020_group
Eggerthellaceae
Mollicutes_RF39
Mitsuokella
Lachnospiraceae_ND3007_group
Streptococcus
Megasphaera
Eubacterium_hallii_group
Anaerostipes
Catenibacterium
Lachnospira
Peptostreptococcaceae
Romboutsia
Veillonellaceae
Selenomonadales
Negativicutes
−4
−3
−2
−1
0
1
2
3
4
LDA SCORE (log 10)

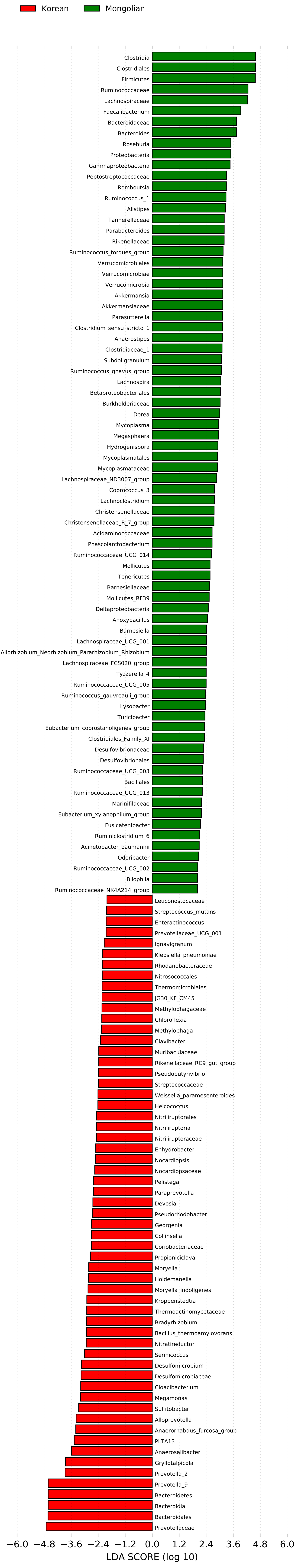
Korean
Mongolian
Clostridia
Clostridiales
Firmicutes
Ruminococcaceae
Lachnospiraceae
Faecalibacterium
Bacteroidaceae
Bacteroides
Roseburia
Proteobacteria
Gammaproteobacteria
Peptostreptococcaceae
Romboutsia
Ruminococcus_1
Alistipes
Tannerellaceae
Parabacteroides
Rikenellaceae
Ruminococcus_torques_group
Verrucomicrobiales
Verrucomicrobiae
Verrucomicrobia
Akkermansia
Akkermansiaceae
Parasutterella
Clostridium_sensu_stricto_1
Anaerostipes
Clostridiaceae_1
Subdoligranulum
Ruminococcus_gnavus_group
Lachnospira
Betaproteobacteriales
Burkholderiaceae
Dorea
Mycoplasma
Megasphaera
Hydrogenispora
Mycoplasmatales
Mycoplasmataceae
Lachnospiraceae_ND3007_group
Coprococcus_3
Lachnoclostridium
Christensenellaceae
Christensenellaceae_R_7_group
Acidaminococcaceae
Phascolarctobacterium
Ruminococcaceae_UCG_014
Mollicutes
Tenericutes
Barnesiellaceae
Mollicutes_RF39
Deltaproteobacteria
Anoxybacillus
Barnesiella
Lachnospiraceae_UCG_001
Allorhizobium_Neorhizobium_Pararhizobium_Rhizobium
Lachnospiraceae_FCS020_group
Tyzzerella_4
Ruminococcaceae_UCG_005
Ruminococcus_gauvreauii_group
Lysobacter
Turicibacter
Eubacterium_coprostanoligenes_group
Clostridiales_Family_XI
Desulfovibrionaceae
Desulfovibrionales
Ruminococcaceae_UCG_003
Bacillales
Ruminococcaceae_UCG_013
Marinifilaceae
Eubacterium_xylanophilum_group
Fusicatenibacter
Ruminiclostridium_6
Acinetobacter_baumannii
Odoribacter
Ruminococcaceae_UCG_002
Bilophila
Ruminococcaceae_NK4A214_group
Leuconostocaceae
Streptococcus_mutans
Enteractinococcus
Prevotellaceae_UCG_001
Ignavigranum
Klebsiella_pneumoniae
Rhodanobacteraceae
Nitrosococcales
Thermomicrobiales
JG30_KF_CM45
Methylophagaceae
Chloroflexia
Methylophaga
Clavibacter
Muribaculaceae
Rikenellaceae_RC9_gut_group
Pseudobutyrivibrio
Streptococcaceae
Weissella_paramesenteroides
Helcococcus
Nitriliruptorales
Nitriliruptoria
Nitriliruptoraceae
Enhydrobacter
Nocardiopsis
Nocardiopsaceae
Pelistega
Paraprevotella
Devosia
Pseudorhodobacter
Georgenia
Collinsella
Coriobacteriaceae
Propioniciclava
Moryella
Holdemanella
Moryella_indoligenes
Kroppenstedtia
Thermoactinomycetaceae
Bradyrhizobium
Bacillus_thermoamylovorans
Nitratireductor
Serinicoccus
Desulfomicrobium
Desulfomicrobiaceae
Cloacibacterium
Megamonas
Sulfitobacter
Alloprevotella
Anaerorhabdus_furcosa_group
PLTA13
Anaerosalibacter
Gryllotalpicola
Prevotella_2
Prevotella_9
Bacteroidetes
Bacteroidia
Bacteroidales
Prevotellaceae
−6.0
−4.8
−3.6
−2.4
−1.2
0.0
1.2
2.4
3.6
4.8
6.0
LDA SCORE (log 10)

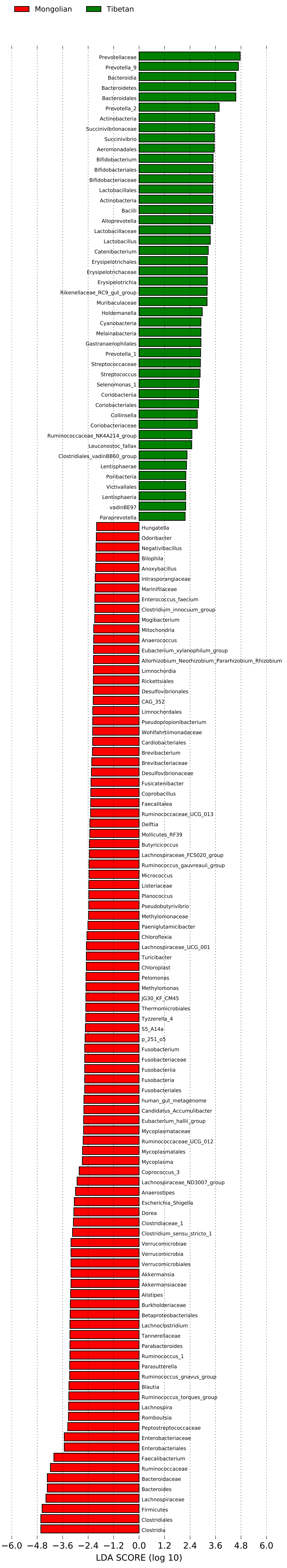
Mongolian
Tibetan
Prevotellaceae
Prevotella_9
Bacteroidia
Bacteroidetes
Bacteroidales
Prevotella_2
Actinobacteria
Succinivibrionaceae
Succinivibrio
Aeromonadales
Bifidobacterium
Bifidobacteriales
Bifidobacteriaceae
Lactobacillales
Actinobacteria
Bacilli
Alloprevotella
Lactobacillaceae
Lactobacillus
Catenibacterium
Erysipelotrichales
Erysipelotrichaceae
Erysipelotrichia
Rikenellaceae_RC9_gut_group
Muribaculaceae
Holdemanella
Cyanobacteria
Melainabacteria
Gastranaerophilales
Prevotella_1
Streptococcaceae
Streptococcus
Selenomonas_1
Coriobacteriia
Coriobacteriales
Collinsella
Coriobacteriaceae
Ruminococcaceae_NK4A214_group
Leuconostoc_fallax
Clostridiales_vadinBB60_group
Lentisphaerae
Poribacteria
Victivallales
Lentisphaeria
vadinBE97
Paraprevotella
Hungatella
Odoribacter
Negativibacillus
Bilophila
Anoxybacillus
Intrasporangiaceae
Marinifilaceae
Enterococcus_faecium
Clostridium_innocuum_group
Mogibacterium
Mitochondria
Anaerococcus
Eubacterium_xylanophilum_group
Allorhizobium_Neorhizobium_Pararhizobium_Rhizobium
Limnochordia
Rickettsiales
Desulfovibrionales
CAG_352
Limnochordales
Pseudopropionibacterium
Wohlfahrtiimonadaceae
Cardiobacteriales
Brevibacterium
Brevibacteriaceae
Desulfovibrionaceae
Fusicatenibacter
Coprobacillus
Faecalitalea
Ruminococcaceae_UCG_013
Delftia
Mollicutes_RF39
Butyricicoccus
Lachnospiraceae_FCS020_group
Ruminococcus_gauvreauii_group
Micrococcus
Listeriaceae
Planococcus
Pseudobutyrivibrio
Methylomonaceae
Paeniglutamicibacter
Chloroflexia
Lachnospiraceae_UCG_001
Turicibacter
Chloroplast
Pelomonas
Methylomonas
JG30_KF_CM45
Thermomicrobiales
Tyzzerella_4
S5_A14a
p_251_o5
Fusobacterium
Fusobacteriaceae
Fusobacteriia
Fusobacteria
Fusobacteriales
human_gut_metagenome
Candidatus_Accumulibacter
Eubacterium_hallii_group
Mycoplasmataceae
Ruminococcaceae_UCG_012
Mycoplasmatales
Mycoplasma
Coprococcus_3
Lachnospiraceae_ND3007_group
Anaerostipes
Escherichia_Shigella
Dorea
Clostridiaceae_1
Clostridium_sensu_stricto_1
Verrucomicrobiae
Verrucomicrobia
Verrucomicrobiales
Akkermansia
Akkermansiaceae
Alistipes
Burkholderiaceae
Betaproteobacteriales
Lachnoclostridium
Tannerellaceae
Parabacteroides
Ruminococcus_1
Parasutterella
Ruminococcus_gnavus_group
Blautia
Ruminococcus_torques_group
Lachnospira
Romboutsia
Peptostreptococcaceae
Enterobacteriaceae
Enterobacteriales
Faecalibacterium
Ruminococcaceae
Bacteroidaceae
Bacteroides
Lachnospiraceae
Firmicutes
Clostridiales
Clostridia
−6.0
−4.8
−3.6
−2.4
−1.2
0.0
1.2
2.4
3.6
4.8
6.0
LDA SCORE (log 10)

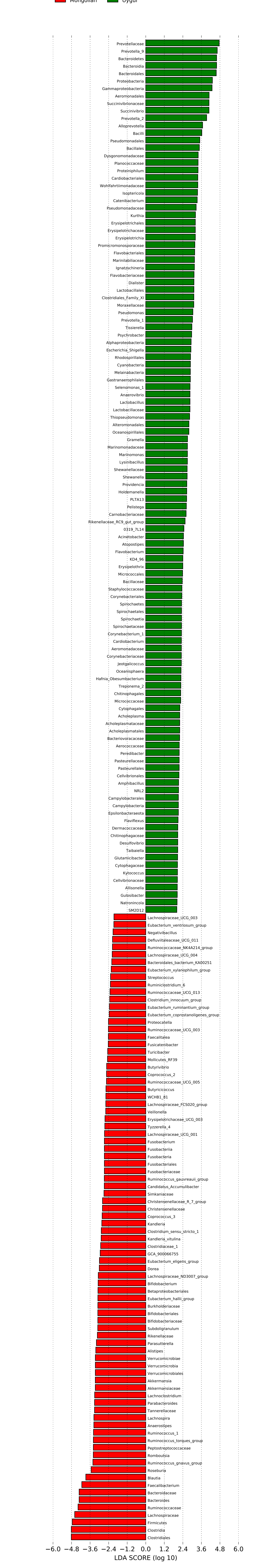
Mongolian
Uygur
Prevotellaceae
Prevotella_9
Bacteroidetes
Bacteroidia
Bacteroidales
Proteobacteria
Gammaproteobacteria
Aeromonadales
Succinivibrionaceae
Succinivibrio
Prevotella_2
Alloprevotella
Bacilli
Pseudomonadales
Bacillales
Dysgonomonadaceae
Planococcaceae
Proteiniphilum
Cardiobacteriales
Wohlfahrtiimonadaceae
Isoptericola
Catenibacterium
Pseudomonadaceae
Kurthia
Erysipelotrichales
Erysipelotrichaceae
Erysipelotrichia
Promicromonosporaceae
Flavobacteriales
Marinilabiliaceae
Ignatzschineria
Flavobacteriaceae
Dialister
Lactobacillales
Clostridiales_Family_XI
Moraxellaceae
Pseudomonas
Prevotella_1
Tissierella
Psychrobacter
Alphaproteobacteria
Escherichia_Shigella
Rhodospirillales
Cyanobacteria
Melainabacteria
Gastranaerophilales
Selenomonas_1
Anaerovibrio
Lactobacillus
Lactobacillaceae
Thiopseudomonas
Alteromonadales
Oceanospirillales
Gramella
Marinomonadaceae
Marinomonas
Lysinibacillus
Shewanellaceae
Shewanella
Providencia
Holdemanella
PLTA13
Pelistega
Carnobacteriaceae
Rikenellaceae_RC9_gut_group
0319_7L14
Acinetobacter
Atopostipes
Flavobacterium
KD4_96
Erysipelothrix
Micrococcales
Bacillaceae
Staphylococcaceae
Corynebacteriales
Spirochaetes
Spirochaetales
Spirochaetia
Spirochaetaceae
Corynebacterium_1
Cardiobacterium
Aeromonadaceae
Corynebacteriaceae
Jeotgalicoccus
Oceanisphaera
Hafnia_Obesumbacterium
Treponema_2
Chitinophagales
Micrococcaceae
Cytophagales
Acholeplasma
Acholeplasmataceae
Acholeplasmatales
Bacteriovoracaceae
Aerococcaceae
Peredibacter
Pasteurellaceae
Pasteurellales
Cellvibrionales
Amphibacillus
NRL2
Campylobacterales
Campylobacteria
Epsilonbacteraeota
Flaviflexus
Dermacoccaceae
Chitinophagaceae
Desulfovibrio
Taibaiella
Glutamicibacter
Cytophagaceae
Kytococcus
Cellvibrionaceae
Allisonella
Gulosibacter
Natronincola
SM2D12
Lachnospiraceae_UCG_003
Eubacterium_ventriosum_group
Negativibacillus
Defluviitaleaceae_UCG_011
Ruminococcaceae_NK4A214_group
Lachnospiraceae_UCG_004
Bacteroidales_bacterium_KA00251
Eubacterium_xylanophilum_group
Streptococcus
Ruminiclostridium_6
Ruminococcaceae_UCG_013
Clostridium_innocuum_group
Eubacterium_ruminantium_group
Eubacterium_coprostanoligenes_group
Proteocatella
Ruminococcaceae_UCG_003
Faecalitalea
Fusicatenibacter
Turicibacter
Mollicutes_RF39
Butyrivibrio
Coprococcus_2
Ruminococcaceae_UCG_005
Butyricicoccus
WCHB1_41
Lachnospiraceae_FCS020_group
Veillonella
Erysipelotrichaceae_UCG_003
Tyzzerella_4
Lachnospiraceae_UCG_001
Fusobacterium
Fusobacteriia
Fusobacteria
Fusobacteriales
Fusobacteriaceae
Ruminococcus_gauvreauii_group
Candidatus_Accumulibacter
Simkaniaceae
Christensenellaceae_R_7_group
Christensenellaceae
Coprococcus_3
Kandleria
Clostridium_sensu_stricto_1
Kandleria_vitulina
Clostridiaceae_1
GCA_900066755
Eubacterium_eligens_group
Dorea
Lachnospiraceae_ND3007_group
Bifidobacterium
Betaproteobacteriales
Eubacterium_hallii_group
Burkholderiaceae
Bifidobacteriales
Bifidobacteriaceae
Subdoligranulum
Rikenellaceae
Parasutterella
Alistipes
Verrucomicrobiae
Verrucomicrobia
Verrucomicrobiales
Akkermansia
Akkermansiaceae
Lachnoclostridium
Parabacteroides
Tannerellaceae
Lachnospira
Anaerostipes
Ruminococcus_1
Ruminococcus_torques_group
Peptostreptococcaceae
Romboutsia
Ruminococcus_gnavus_group
Roseburia
Blautia
Faecalibacterium
Bacteroidaceae
Bacteroides
Ruminococcaceae
Lachnospiraceae
Firmicutes
Clostridia
Clostridiales
−6.0
−4.8
−3.6
−2.4
−1.2
0.0
1.2
2.4
3.6
4.8
6.0
LDA SCORE (log 10)

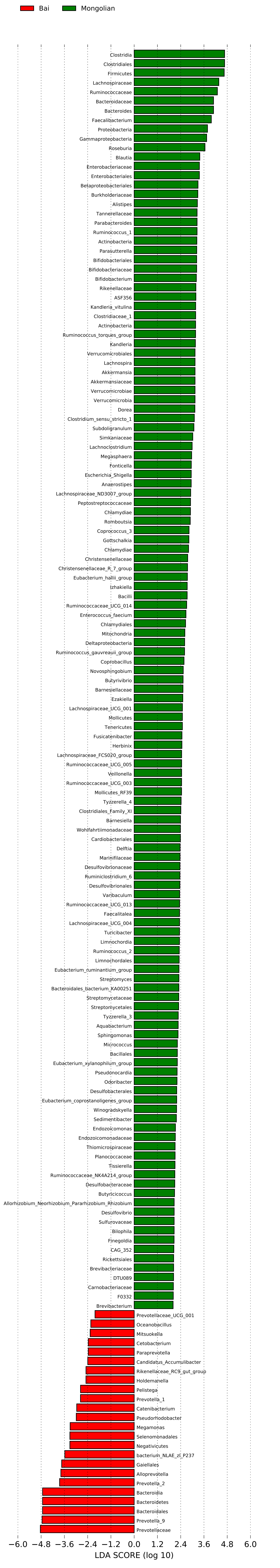
Bai
Mongolian
Clostridia
Clostridiales
Firmicutes
Lachnospiraceae
Ruminococcaceae
Bacteroidaceae
Bacteroides
Faecalibacterium
Proteobacteria
Gammaproteobacteria
Roseburia
Blautia
Enterobacteriaceae
Enterobacteriales
Betaproteobacteriales
Burkholderiaceae
Alistipes
Tannerellaceae
Parabacteroides
Ruminococcus_1
Actinobacteria
Parasutterella
Bifidobacteriales
Bifidobacteriaceae
Bifidobacterium
Rikenellaceae
ASF356
Kandleria_vitulina
Clostridiaceae_1
Actinobacteria
Ruminococcus_torques_group
Kandleria
Verrucomicrobiales
Lachnospira
Akkermansia
Akkermansiaceae
Verrucomicrobiae
Verrucomicrobia
Dorea
Clostridium_sensu_stricto_1
Subdoligranulum
Simkaniaceae
Lachnoclostridium
Megasphaera
Fonticella
Escherichia_Shigella
Anaerostipes
Lachnospiraceae_ND3007_group
Peptostreptococcaceae
Chlamydiae
Romboutsia
Coprococcus_3
Gottschalkia
Chlamydiae
Christensenellaceae
Christensenellaceae_R_7_group
Eubacterium_hallii_group
Izhakiella
Bacilli
Ruminococcaceae_UCG_014
Enterococcus_faecium
Chlamydiales
Mitochondria
Deltaproteobacteria
Ruminococcus_gauvreauii_group
Coprobacillus
Novosphingobium
Butyrivibrio
Barnesiellaceae
Ezakiella
Lachnospiraceae_UCG_001
Mollicutes
Tenericutes
Fusicatenibacter
Herbinix
Lachnospiraceae_FCS020_group
Ruminococcaceae_UCG_005
Veillonella
Ruminococcaceae_UCG_003
Mollicutes_RF39
Tyzzerella_4
Clostridiales_Family_XI
Barnesiella
Wohlfahrtiimonadaceae
Cardiobacteriales
Delftia
Marinifilaceae
Desulfovibrionaceae
Ruminiclostridium_6
Desulfovibrionales
Vanbaculum
Ruminococcaceae_UCG_013
Faecalitalea
Lachnospiraceae_UCG_004
Turicibacter
Limnochordia
Ruminococcus_2
Limnochordales
Eubacterium_ruminantium_group
Streptomyces
Bacteroidales_bacterium_KA00251
Streptomycetaceae
Streptomycetales
Tyzzerella_3
Aquabacterium
Sphingomonas
Micrococcus
Bacillales
Eubacterium_xylanophilum_group
Pseudonocardia
Odoribacter
Desulfobacterales
Eubacterium_coprostanoligenes_group
Winogradskyella
Sedimentibacter
Endozoicomonas
Endozoicomonadaceae
Thiomicrospiraceae
Planococcaceae
Tissierella
Ruminococcaceae_NK4A214_group
Desulfobacteraceae
Butyricicoccus
Allorhizobium_Neorhizobium_Pararhizobium_Rhizobium
Desulfovibrio
Sulfurovaceae
Bilophila
Finegoldia
CAG_352
Rickettsiales
Brevibacteriaceae
DTU089
Carnobacteriaceae
F0332
Brevibacterium
Prevotellaceae_UCG_001
Oceanobacillus
Mitsuokella
Cetobacterium
Paraprevotella
Candidatus_Accumulibacter
Rikenellaceae_RC9_gut_group
Holdemanella
Pelistega
Prevotella_1
Catenibacterium
Pseudorhodobacter
Megamonas
Selenomonadales
Negativicutes
bacterium_NLAE_zl_P237
Gaiellales
Alloprevotella
Prevotella_2
Bacteroidia
Bacteroidetes
Bacteroidales
Prevotella_9
Prevotellaceae
−6.0
−4.8
−3.6
−2.4
−1.2
0.0
1.2
2.4
3.6
4.8
6.0
LDA SCORE (log 10)

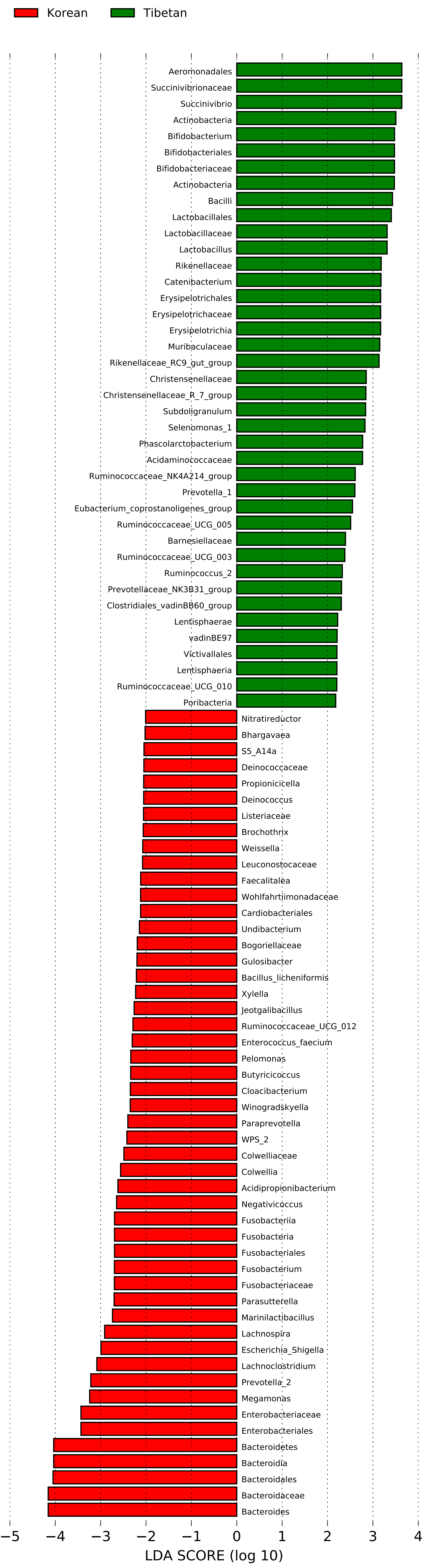
Korean
Tibetan
Aeromonadales
Succinivibrionaceae
Succinivibrio
Actinobacteria
Bifidobacterium
Bifidobacteriales
Bifidobacteriaceae
Actinobacteria
Bacilli
Lactobacillales
Lactobacillaceae
Lactobacillus
Rikenellaceae
Catenibacterium
Erysipelotrichales
Erysipelotrichaceae
Erysipelotrichia
Muribaculaceae
Rikenellaceae_RC9_gut_group
Christensenellaceae
Christensenellaceae_R_7_group
Subdoligranulum
Selenomonas_1
Phascolarctobacterium
Acidaminococcaceae
Ruminococcaceae_NK4A214_group
Prevotella_1
Eubacterium_coprostanoligenes_group
Ruminococcaceae_UCG_005
Barnesiellaceae
Ruminococcaceae_UCG_003
Ruminococcus_2
Prevotellaceae_NK3B31_group
Clostridiales_vadinBB60_group
Lentisphaerae
vadinBE97
Victivallales
Lentisphaeria
Ruminococcaceae_UCG_010
Poribacteria
Nitratireductor
Bhargavaea
S5_A14a
Deinococcaceae
Propionicicella
Deinococcus
Listeriaceae
Brochothrix
Weissella
Leuconostocaceae
Faecalitalea
Wohlfahrtiimonadaceae
Cardiobacteriales
Undibacterium
Bogoriellaceae
Gulosibacter
Bacillus_licheniformis
Xylella
Jeotgalibacillus
Ruminococcaceae_UCG_012
Enterococcus_faecium
Pelomonas
Butyricicoccus
Cloacibacterium
Winogradskyella
Paraprevotella
WPS_2
Colwelliaceae
Colwellia
Acidipropionibacterium
Negativicoccus
Fusobacteriia
Fusobacteria
Fusobacteriales
Fusobacterium
Fusobacteriaceae
Parasutterella
Marinilactibacillus
Lachnospira
Escherichia_Shigella
Lachnoclostridium
Prevotella_2
Megamonas
Enterobacteriaceae
Enterobacteriales
Bacteroidetes
Bacteroidia
Bacteroidales
Bacteroidaceae
Bacteroides
−5
−4
−3
−2
−1
0
1
2
3
4
LDA SCORE (log 10)

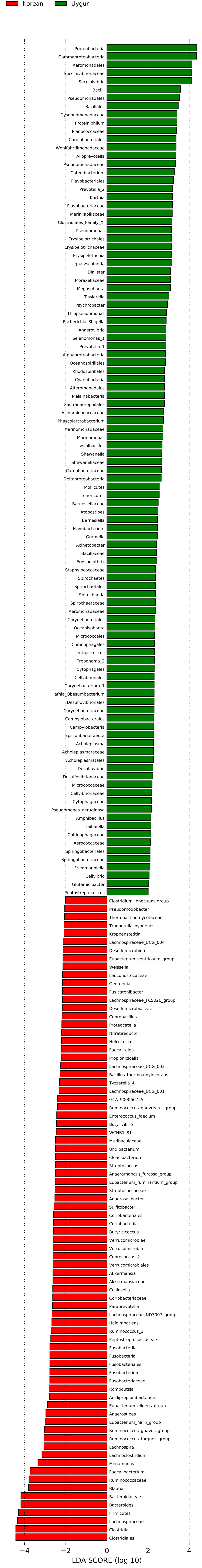

Korean
Uygur
Proteobacteria
Gammaproteobacteria
Aeromonadales
Succinivibrionaceae
Succinivibrio
Bacilli
Pseudomonadales
Bacillales
Dysgonomonadaceae
Proteiniphilum
Planococcaceae
Cardiobacteriales
Wohlfahrtiimonadaceae
Alloprevotella
Pseudomonadaceae
Catenibacterium
Flavobacteriales
Prevotella_2
Kurthia
Flavobacteriaceae
Marinilabiliaceae
Clostridiales_Family_XI
Pseudomonas
Erysipelotrichales
Erysipelotrichaceae
Erysipelotrichia
Ignatzschineria
Dialister
Moraxellaceae
Megasphaera
Tissierella
Psychrobacter
Thiopseudomonas
Escherichia_Shigella
Anaerovibrio
Selenomonas_1
Prevotella_1
Alphaproteobacteria
Oceanospirillales
Rhodospirillales
Cyanobacteria
Alteromonadales
Melainabacteria
Gastranaerophilales
Acidaminococcaceae
Phascolarctobacterium
Marinomonadaceae
Marinomonas
Lysinibacillus
Shewanella
Shewanellaceae
Carnobacteriaceae
Deltaproteobacteria
Mollicutes
Tenericutes
Barnesiellaceae
Atopostipes
Barnesiella
Flavobacterium
Gramella
Acinetobacter
Bacillaceae
Erysipelothrix
Staphylococcaceae
Spirochaetes
Spirochaetales
Spirochaetia
Spirochaetaceae
Aeromonadaceae
Corynebacteriales
Oceanisphaera
Micrococcales
Chitinophagales
Jeotgalicoccus
Treponema_2
Cytophagales
Cellvibrionales
Corynebacterium_1
Hafnia_Obesumbacterium
Desulfovibrionales
Corynebacteriaceae
Campylobacterales
Campylobacteria
Epsilonbacteraeota
Acholeplasma
Acholeplasmataceae
Acholeplasmatales
Desulfovibrio
Desulfovibrionaceae
Micrococcaceae
Cellvibrionaceae
Cytophagaceae
Pseudomonas_aeruginosa
Amphibacillus
Taibaiella
Chitinophagaceae
Aerococcaceae
Sphingobacteriales
Sphingobacteriaceae
Friedmanniella
Cellvibrio
Glutamicibacter
Peptostreptococcus
Clostridium_innocuum_group
Pseudorhodobacter
Thermoactinomycetaceae
Trueperella_pyogenes
Kroppenstedtia
Lachnospiraceae_UCG_004
Desulfomicrobium
Eubacterium_ventriosum_group
Weissella
Leuconostocaceae
Georgenia
Fusicatenibacter
Lachnospiraceae_FCS020_group
Desulfomicrobiaceae
Coprobacillus
Proteocatella
Nitratireductor
Helcococcus
Faecalitalea
Propionicicella
Lachnospiraceae_UCG_003
Bacillus_thermoamylovorans
Tyzzerella_4
Lachnospiraceae_UCG_001
GCA_900066755
Ruminococcus_gauvreauii_group
Enterococcus_faecium
Butyrivibrio
WCHB1_81
Muribaculaceae
Undibacterium
Cloacibacterium
Streptococcus
Anaerorhabdus_furcosa_group
Eubacterium_ruminantium_group
Streptococcaceae
Anaerosalibacter
Sulfitobacter
Coriobacteriales
Coriobacteriia
Butyricicoccus
Verrucomicrobiae
Verrucomicrobia
Coprococcus_2
Verrucomicrobiales
Akkermansia
Akkermansiaceae
Collinsella
Coriobacteriaceae
Paraprevotella
Lachnospiraceae_ND3007_group
Haloimpatiens
Ruminococcus_1
Peptostreptococcaceae
Fusobacteriia
Fusobacteria
Fusobacteriales
Fusobacterium
Fusobacteriaceae
Romboutsia
Acidipropionibacterium
Eubacterium_eligens_group
Anaerostipes
Eubacterium_hallii_group
Ruminococcus_gnavus_group
Ruminococcus_torques_group
Lachnospira
Lachnoclostridium
Megamonas
Faecalibacterium
Ruminococcaceae
Blautia
Bacteroidaceae
Bacteroides
Firmicutes
Lachnospiraceae
Clostridia
Clostridiales
−4
−2
0
2
4
LDA SCORE (log 10)

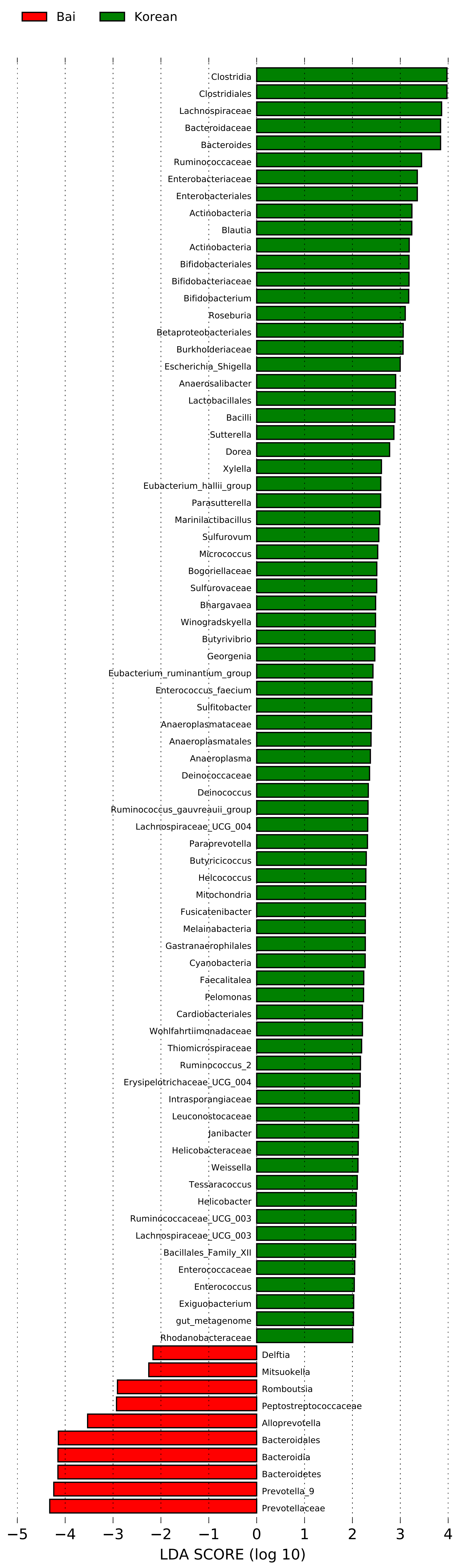
Bai
Korean
Clostridia
Clostridiales
Lachnospiraceae
Bacteroidaceae
Bacteroides
Ruminococcaceae
Enterobacteriaceae
Enterobacteriales
Actinobacteria
Blautia
Actinobacteria
Bifidobacteriales
Bifidobacteriaceae
Bifidobacterium
Roseburia
Betaproteobacteriales
Burkholderiaceae
Escherichia_Shigella
Anaerosalibacter
Lactobacillales
Bacilli
Sutterella
Dorea
Xylella
Eubacterium_hallii_group
Parasutterella
Marinilactibacillus
Sulfurovum
Micrococcus
Bogoriellaceae
Sulfurovaceae
Bhargavaea
Winogradskyella
Butyrivibrio
Georgenia
Eubacterium_ruminantium_group
Enterococcus_faecium
Sulfitobacter
Anaeroplasmataceae
Anaeroplasmatales
Anaeroplasma
Deinococcaceae
Deinococcus
Ruminococcus_gauvreauii_group
Lachnospiraceae_UCG_004
Paraprevotella
Butyricicoccus
Helcococcus
Mitochondria
Fusicatenibacter
Melainabacteria
Gastranaerophilales
Cyanobacteria
Faecalitalea
Pelomonas
Cardiobacteriales
Wohlfahrtiimonadaceae
Thiomicrospiraceae
Ruminococcus_2
Erysipelotrichaceae_UCG_004
Intrasporangiaceae
Leuconostocaceae
Janibacter
Helicobacteraceae
Weissella
Tessaracoccus
Helicobacter
Ruminococcaceae_UCG_003
Lachnospiraceae_UCG_003
Bacillales_Family_XII
Enterococcaceae
Enterococcus
Exiguobacterium
gut_metagenome
Rhodanobacteraceae
Delftia
Mitsuokella
Romboutsia
Peptostreptococcaceae
Alloprevotella
Bacteroidales
Bacteroidia
Bacteroidetes
Prevotella_9
Prevotellaceae
−5
−4
−3
−2
−1
0
1
2
3
4
LDA SCORE (log 10)

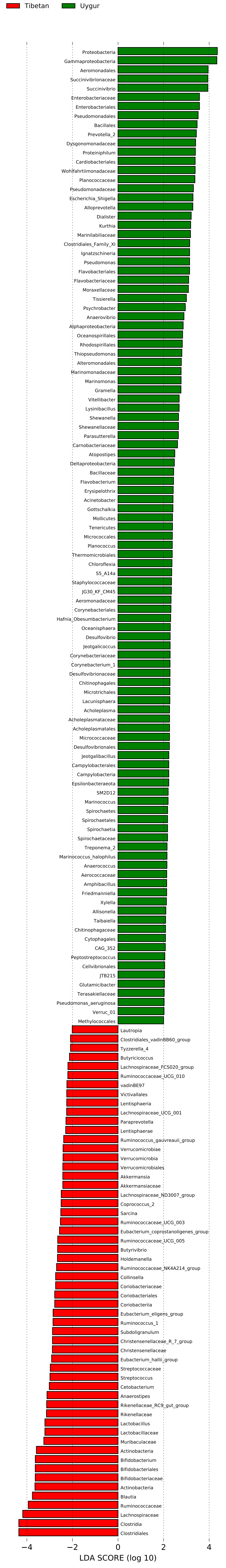

Tibetan
Uygur
Proteobacteria
Gammaproteobacteria
Aeromonadales
Succinivibrionaceae
Succinivibrio
Enterobacteriaceae
Enterobacteriales
Pseudomonadales
Bacillales
Prevotella_2
Dysgonomonadaceae
Proteiniphilum
Cardiobacteriales
Wohlfahrtiimonadaceae
Planococcaceae
Pseudomonadaceae
Escherichia_Shigella
Alloprevotella
Dialister
Kurthia
Marinilabiliaceae
Clostridiales_Family_XI
Ignatzschineria
Pseudomonas
Flavobacteriales
Flavobacteriaceae
Moraxellaceae
Tissierella
Psychrobacter
Anaerovibrio
Alphaproteobacteria
Oceanospirillales
Rhodospirillales
Thiopseudomonas
Alteromonadales
Marinomonadaceae
Marinomonas
Gramella
Vitellibacter
Lysinibacillus
Shewanella
Shewanellaceae
Parasutterella
Carnobacteriaceae
Atopostipes
Deltaproteobacteria
Bacillaceae
Flavobacterium
Erysipelothrix
Acinetobacter
Gottschalkia
Mollicutes
Tenericutes
Micrococcales
Planococcus
Thermomicrobiales
Chloroflexia
S5_A14a
Staphylococcaceae
JG30_KF_CM45
Aeromonadaceae
Corynebacteriales
Hafnia_Obesumbacterium
Oceanisphaera
Desulfovibrio
Jeotgalicoccus
Corynebacteriaceae
Corynebacterium_1
Desulfovibrionaceae
Chitinophagales
Microtrichales
Lacunisphaera
Acholeplasma
Acholeplasmataceae
Acholeplasmatales
Micrococcaceae
Desulfovibrionales
Jeotgalibacillus
Campylobacterales
Campylobacteria
Epsilonbacteraeota
SM2D12
Marinococcus
Spirochaetes
Spirochaetales
Spirochaetia
Spirochaetaceae
Treponema_2
Marinococcus_halophilus
Anaerococcus
Aerococcaceae
Amphibacillus
Friedmanniella
Xylella
Allisonella
Taibaiella
Chitinophagaceae
Cytophagales
CAG_352
Peptostreptococcus
Cellvibrionales
JTB215
Glutamicibacter
Terasakiellaceae
Pseudomonas_aeruginosa
Verruc_01
Methylococcales
Lautropia
Clostridiales_vadinBB60_group
Tyzzerella_4
Butyricicoccus
Lachnospiraceae_FCS020_group
Ruminococcaceae_UCG_010
vadinBE97
Victivallales
Lentisphaeria
Lachnospiraceae_UCG_001
Paraprevotella
Lentisphaerae
Ruminococcus_gauvreauii_group
Verrucomicrobiae
Verrucomicrobia
Verrucomicrobiales
Akkermansia
Akkermansiaceae
Lachnospiraceae_ND3007_group
Coprococcus_2
Sarcina
Ruminococcaceae_UCG_003
Eubacterium_coprostanoligenes_group
Ruminococcaceae_UCG_005
Butyrivibrio
Holdemanella
Ruminococcaceae_NK4A214_group
Collinsella
Coriobacteriaceae
Coriobacteriales
Coriobacteriia
Eubacterium_eligens_group
Ruminococcus_1
Subdoligranulum
Christensenellaceae_R_7_group
Christensenellaceae
Eubacterium_hallii_group
Streptococcaceae
Streptococcus
Cetobacterium
Anaerostipes
Rikenellaceae_RC9_gut_group
Rikenellaceae
Lactobacillus
Lactobacillaceae
Muribaculaceae
Actinobacteria
Bifidobacterium
Bifidobacteriales
Bifidobacteriaceae
Actinobacteria
Blautia
Ruminococcaceae
Lachnospiraceae
Clostridia
Clostridiales
−4
−2
0
2
4
LDA SCORE (log 10)

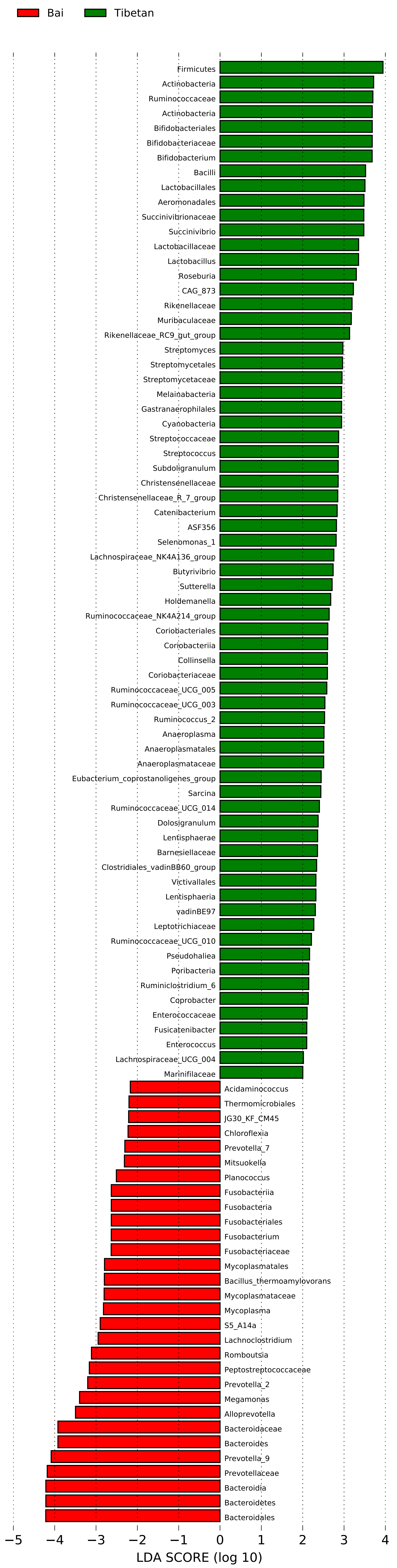

Bai
Tibetan
Firmicutes
Actinobacteria
Ruminococcaceae
Actinobacteria
Bifidobacteriales
Bifidobacteriaceae
Bifidobacterium
Bacilli
Lactobacillales
Aeromonadales
Succinivibrionaceae
Succinivibrio
Lactobacillaceae
Lactobacillus
Roseburia
CAG_873
Rikenellaceae
Muribaculaceae
Rikenellaceae_RC9_gut_group
Streptomyces
Streptomycetales
Streptomycetaceae
Melainabacteria
Gastranaerophilales
Cyanobacteria
Streptococcaceae
Streptococcus
Subdoligranulum
Christensenellaceae
Christensenellaceae_R_7_group
Catenibacterium
ASF356
Selenomonas_1
Lachnospiraceae_NK4A136_group
Butyrivibrio
Sutterella
Holdemanella
Ruminococcaceae_NK4A214_group
Coriobacteriales
Coriobacteriia
Collinsella
Coriobacteriaceae
Ruminococcaceae_UCG_005
Ruminococcaceae_UCG_003
Ruminococcus_2
Anaeroplasma
Anaeroplasmatales
Anaeroplasmataceae
Eubacterium_coprostanoligenes_group
Sarcina
Ruminococcaceae_UCG_014
Dolosigranulum
Lentisphaerae
Barnesiellaceae
Clostridiales_vadinBB60_group
Victivallales
Lentisphaeria
vadinBE97
Leptotrichiaceae
Ruminococcaceae_UCG_010
Pseudohaliea
Poribacteria
Ruminiclostridium_6
Coprobacter
Enterococcaceae
Fusicatenibacter
Enterococcus
Lachnospiraceae_UCG_004
Marinifilaceae
Acidaminococcus
Thermomicrobiales
JG30_KF_CM45
Chloroflexia
Prevotella_7
Mitsuokella
Planococcus
Fusobacteriia
Fusobacteria
Fusobacteriales
Fusobacterium
Fusobacteriaceae
Mycoplasmatales
Bacillus_thermoamylovorans
Mycoplasmataceae
Mycoplasma
S5_A14a
Lachnoclostridium
Romboutsia
Peptostreptococcaceae
Prevotella_2
Megamonas
Alloprevotella
Bacteroidaceae
Bacteroides
Prevotella_9
Prevotellaceae
Bacteroidia
Bacteroidetes
Bacteroidales
−5
−4
−3
−2
−1
0
1
2
3
4
LDA SCORE (log 10)

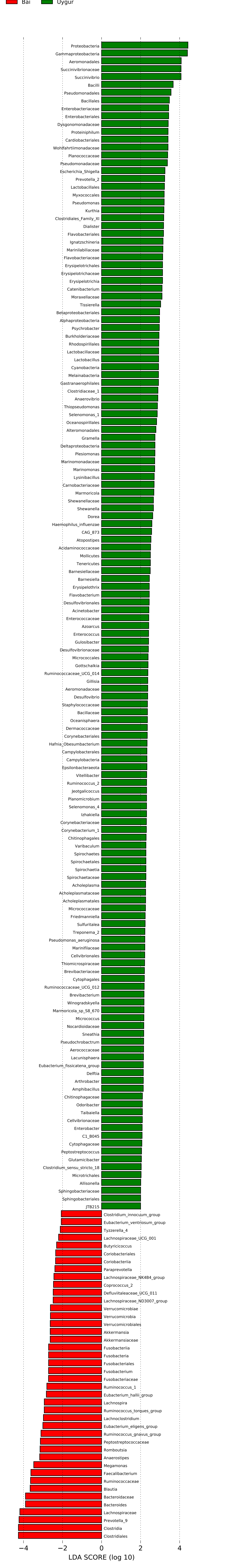

Bai
Uygur
Proteobacteria
Gammaproteobacteria
Aeromonadales
Succinivibrionaceae
Succinivibrio
Bacilli
Pseudomonadales
Bacillales
Enterobacteriaceae
Enterobacteriales
Dysgonomonadaceae
Proteiniphilum
Cardiobacteriales
Wohlfahrtiimonadaceae
Planococcaceae
Pseudomonadaceae
Escherichia_Shigella
Prevotella_2
Lactobacillales
Myxococcales
Pseudomonas
Kurthia
Clostridiales_Family_XI
Dialister
Flavobacteriales
Ignatzschineria
Marinilabiliaceae
Flavobacteriaceae
Erysipelotrichales
Erysipelotrichaceae
Erysipelotrichia
Catenibacterium
Moraxellaceae
Tissierella
Betaproteobacteriales
Alphaproteobacteria
Psychrobacter
Burkholderiaceae
Rhodospirillales
Lactobacillaceae
Lactobacillus
Cyanobacteria
Melainabacteria
Gastranaerophilales
Clostridiaceae_1
Anaerovibrio
Thiopseudomonas
Selenomonas_1
Oceanospirillales
Alteromonadales
Gramella
Deltaproteobacteria
Plesiomonas
Marinomonadaceae
Marinomonas
Lysinibacillus
Carnobacteriaceae
Marmoricola
Shewanellaceae
Shewanella
Dorea
Haemophilus_influenzae
CAG_873
Atopostipes
Acidaminococcaceae
Mollicutes
Tenericutes
Barnesiellaceae
Barnesiella
Erysipelothrix
Flavobacterium
Desulfovibrionales
Acinetobacter
Enterococcaceae
Azoarcus
Enterococcus
Gulosibacter
Desulfovibrionaceae
Micrococcales
Gottschalkia
Ruminococcaceae_UCG_014
Gillisia
Aeromonadaceae
Desulfovibrio
Staphylococcaceae
Bacillaceae
Oceanisphaera
Dermacoccaceae
Corynebacteriales
Hafnia_Obesumbacterium
Campylobacterales
Campylobacteria
Epsilonbacteraeota
Vitellibacter
Ruminococcus_2
Jeotgalicoccus
Planomicrobium
Selenomonas_4
Izhakiella
Corynebacteriaceae
Corynebacterium_1
Chitinophagales
Varibaculum
Spirochaetes
Spirochaetales
Spirochaetia
Spirochaetaceae
Acholeplasma
Acholeplasmataceae
Acholeplasmatales
Micrococcaceae
Friedmanniella
Sulfuritalea
Treponema_2
Pseudomonas_aeruginosa
Marinifilaceae
Cellvibrionales
Thiomicrospiraceae
Brevibacteriaceae
Cytophagales
Ruminococcaceae_UCG_012
Brevibacterium
Winogradskyella
Marmoricola_sp_S8_670
Micrococcus
Nocardioidaceae
Sneathia
Pseudochrobactrum
Aerococcaceae
Lacunisphaera
Eubacterium_fissicatena_group
Delftia
Arthrobacter
Amphibacillus
Chitinophagaceae
Odoribacter
Taibaiella
Cellvibrionaceae
Enterobacter
C1_B045
Cytophagaceae
Peptostreptococcus
Glutamicibacter
Clostridium_sensu_stricto_18
Microtrichales
Allisonella
Sphingobacteriaceae
Sphingobacteriales
JTB215
Clostridium_innocuum_group
Eubacterium_ventriosum_group
Tyzzerella_4
Lachnospiraceae_UCG_001
Butyricicoccus
Coriobacteriales
Coriobacteriia
Paraprevotella
Lachnospiraceae_NK4B4_group
Coprococcus_2
Defluviitaleaceae_UCG_011
Lachnospiraceae_ND3007_group
Verrucomicrobiae
Verrucomicrobia
Verrucomicrobiales
Akkermansia
Akkermansiaceae
Fusobacteriia
Fusobacteria
Fusobacteriales
Fusobacterium
Fusobacteriaceae
Ruminococcus_1
Eubacterium_hallii_group
Lachnospira
Ruminococcus_torques_group
Lachnoclostridium
Eubacterium_eligens_group
Ruminococcus_gnavus_group
Peptostreptococcaceae
Romboutsia
Anaerostipes
Megamonas
Faecalibacterium
Ruminococcaceae
Blautia
Bacteroidaceae
Bacteroides
Lachnospiraceae
Prevotella_9
Clostridia
Clostridiales
−4
−2
0
2
4
LDA SCORE (log 10)

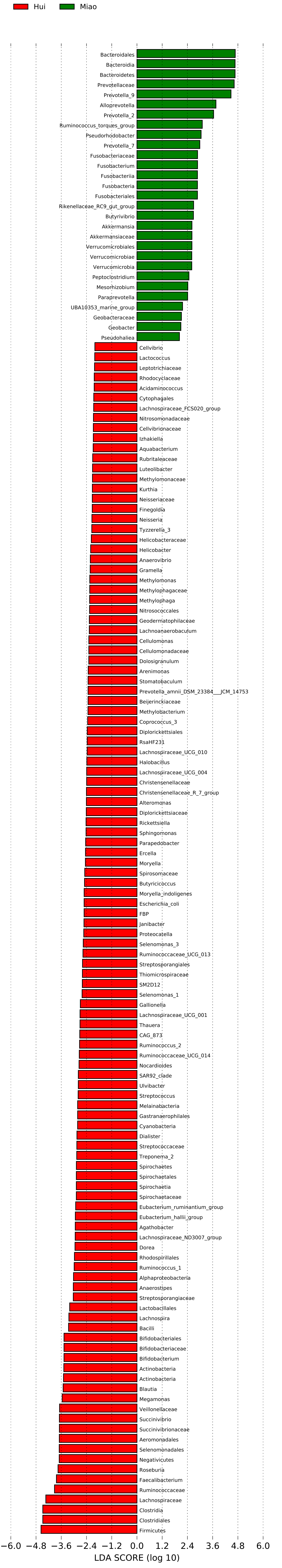

Hui
Miao
Bacteroidales
Bacteroidia
Bacteroidetes
Prevotellaceae
Prevotella_9
Alloprevotella
Prevotella_2
Ruminococcus_torques_group
Pseudorhodobacter
Prevotella_7
Fusobacteriaceae
Fusobacterium
Fusobacteriia
Fusobacteria
Fusobacteriales
Rikenellaceae_RC9_gut_group
Butyrivibrio
Akkermansia
Akkermansiaceae
Verrucomicrobiales
Verrucomicrobiae
Verrucomicrobia
Peptoclostridium
Mesorhizobium
Paraprevotella
UBA10353_marine_group
Geobacteraceae
Geobacter
Pseudohaliea
Cellvibrio
Lactococcus
Leptotrichiaceae
Rhodocyclaceae
Acidaminococcus
Cytophagales
Lachnospiraceae_FCS020_group
Nitrosomonadaceae
Cellvibrionaceae
Izhakiella
Aquabacterium
Rubritaleaceae
Luteolibacter
Methylomonaceae
Kurthia
Neisseriaceae
Finegoldia
Neisseria
Tyzzerella_3
Helicobacteraceae
Helicobacter
Anaerovibrio
Gramella
Methylomonas
Methylophagaceae
Methylophaga
Nitrosococcales
Geodermatophilaceae
Lachnoanaerobaculum
Cellulomonas
Cellulomonadaceae
Dolosigranulum
Arenimonas
Stomatobaculum
Prevotella_amnii_DSM_23384__JCM_14753
Beijerinckiaceae
Methylobacterium
Coprococcus_3
Diplorickettsiales
RsaHF231
Lachnospiraceae_UCG_010
Halobacillus
Lachnospiraceae_UCG_004
Christensenellaceae
Christensenellaceae_R_7_group
Alteromonas
Diplorickettsiaceae
Rickettsiella
Sphingomonas
Parapedobacter
Ercella
Moryella
Spirosomaceae
Butyricicoccus
Moryella_indoligenes
Escherichia_coli
FBP
Janibacter
Proteocatella
Selenomonas_3
Ruminococcaceae_UCG_013
Streptosporangiales
Thiomicrospiraceae
SM2D12
Selenomonas_1
Gallionella
Lachnospiraceae_UCG_001
Thauera
CAG_873
Ruminococcus_2
Ruminococcaceae_UCG_014
Nocardioides
SAR92_clade
Ulvibacter
Streptococcus
Melainabacteria
Gastranaerophilales
Cyanobacteria
Dialister
Streptococcaceae
Treponema_2
Spirochaetes
Spirochaetales
Spirochaetia
Spirochaetaceae
Eubacterium_ruminantium_group
Eubacterium_hallii_group
Agathobacter
Lachnospiraceae_ND3007_group
Dorea
Rhodospirillales
Ruminococcus_1
Alphaproteobacteria
Anaerostipes
Streptosporangiaceae
Lactobacillales
Lachnospira
Bacilli
Bifidobacteriales
Bifidobacteriaceae
Bifidobacterium
Actinobacteria
Actinobacteria
Blautia
Megamonas
Veillonellaceae
Succinivibrio
Succinivibrionaceae
Aeromonadales
Selenomonadales
Negativicutes
Roseburia
Faecalibacterium
Ruminococcaceae
Lachnospiraceae
Clostridia
Clostridiales
Firmicutes
−6.0
−4.8
−3.6
−2.4
−1.2
0.0
1.2
2.4
3.6
4.8
6.0
LDA SCORE (log 10)

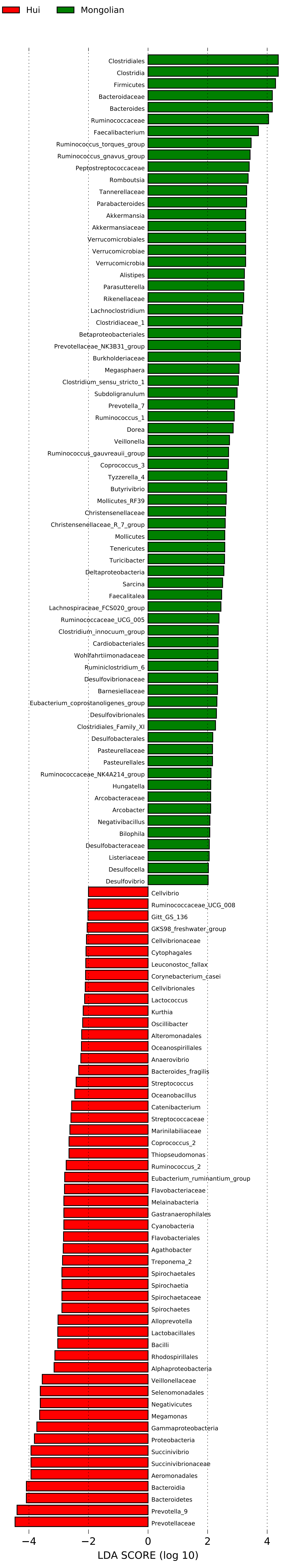

Hui
Mongolian
Clostridiales
Clostridia
Firmicutes
Bacteroidaceae
Bacteroides
Ruminococcaceae
Faecalibacterium
Ruminococcus_torques_group
Ruminococcus_gnavus_group
Peptostreptococcaceae
Romboutsia
Tannerellaceae
Parabacteroides
Akkermansia
Akkermansiaceae
Verrucomicrobiales
Verrucomicrobiae
Verrucomicrobia
Alistipes
Parasutterella
Rikenellaceae
Lachnoclostridium
Clostridiaceae_1
Betaproteobacteriales
Prevotellaceae_NK3B31_group
Burkholderiaceae
Megasphaera
Clostridium_sensu_stricto_1
Subdoligranulum
Prevotella_7
Ruminococcus_1
Dorea
Veillonella
Ruminococcus_gauvreauii_group
Coprococcus_3
Tyzzerella_4
Butyrivibrio
Mollicutes_RF39
Christensenellaceae
Christensenellaceae_R_7_group
Mollicutes
Tenericutes
Turicibacter
Deltaproteobacteria
Sarcina
Faecalitalea
Lachnospiraceae_FCS020_group
Ruminococcaceae_UCG_005
Clostridium_innocuum_group
Cardiobacteriales
Wohlfahrtiimonadaceae
Ruminiclostridium_6
Desulfovibrionaceae
Barnesiellaceae
Eubacterium_coprostanoligenes_group
Desulfovibrionales
Clostridiales_Family_XI
Desulfobacterales
Pasteurellaceae
Pasteurellales
Ruminococcaceae_NK4A214_group
Hungatella
Arcobacteraceae
Arcobacter
Negativibacillus
Bilophila
Desulfobacteraceae
Listeriaceae
Desulfocella
Desulfovibrio
Cellvibrio
Ruminococcaceae_UCG_008
Gitt_GS_136
GKS98_freshwater_group
Cellvibrionaceae
Cytophagales
Leuconostoc_fallax
Corynebacterium_casei
Cellvibrionales
Lactococcus
Kurthia
Oscillibacter
Alteromonadales
Oceanospirillales
Anaerovibrio
Bacteroides_fragilis
Streptococcus
Oceanobacillus
Catenibacterium
Streptococcaceae
Marinilabiliaceae
Coprococcus_2
Thiopseudomonas
Ruminococcus_2
Eubacterium_ruminantium_group
Flavobacteriaceae
Melainabacteria
Gastranaerophilales
Cyanobacteria
Flavobacteriales
Agathobacter
Treponema_2
Spirochaetales
Spirochaetia
Spirochaetaceae
Spirochaetes
Alloprevotella
Lactobacillales
Bacilli
Rhodospirillales
Alphaproteobacteria
Veillonellaceae
Selenomonadales
Negativicutes
Megamonas
Gammaproteobacteria
Proteobacteria
Succinivibrio
Succinivibrionaceae
Aeromonadales
Bacteroidia
Bacteroidetes
Prevotella_9
Prevotellaceae
−4
−2
0
2
4
LDA SCORE (log 10)

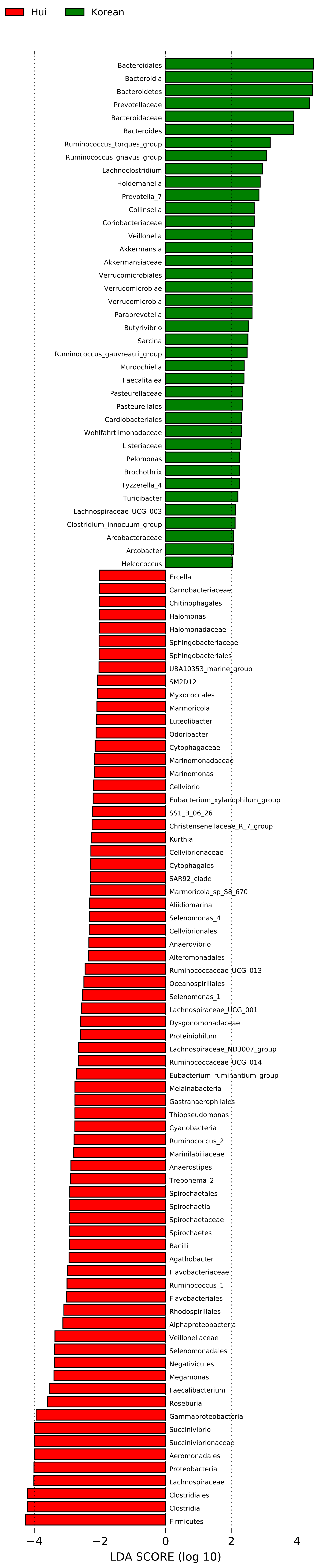
Hui
Korean
Bacteroidales
Bacteroidia
Bacteroidetes
Prevotellaceae
Bacteroidaceae
Bacteroides
Ruminococcus_torques_group
Ruminococcus_gnavus_group
Lachnoclostridium
Holdemanella
Prevotella_7
Collinsella
Coriobacteriaceae
Veillonella
Akkermansia
Akkermansiaceae
Verrucomicrobiales
Verrucomicrobiae
Verrucomicrobia
Paraprevotella
Butyrivibrio
Sarcina
Ruminococcus_gauvreauii_group
Murdochiella
Faecalitalea
Pasteurellaceae
Pasteurellales
Cardiobacteriales
Wohlfahrtiimonadaceae
Listeriaceae
Pelomonas
Brochothrix
Tyzzerella_4
Turicibacter
Lachnospiraceae_UCG_003
Clostridium_innocuum_group
Arcobacteraceae
Arcobacter
Helcococcus
Ercella
Carnobacteriaceae
Chitinophagales
Halomonas
Halomonadaceae
Sphingobacteriaceae
Sphingobacteriales
UBA10353_marine_group
SM2D12
Myxococcales
Marmoricola
Luteolibacter
Odoribacter
Cytophagaceae
Marinomonadaceae
Marinomonas
Cellvibrio
Eubacterium_xylanophilum_group
SS1_B_06_26
Christensenellaceae_R_7_group
Kurthia
Cellvibrionaceae
Cytophagales
SAR92_clade
Marmoricola_sp_S8_670
Aliidiomarina
Selenomonas_4
Cellvibrionales
Anaerovibrio
Alteromonadales
Ruminococcaceae_UCG_013
Oceanospirillales
Selenomonas_1
Lachnospiraceae_UCG_001
Dysgonomonadaceae
Proteiniphilum
Lachnospiraceae_ND3007_group
Ruminococcaceae_UCG_014
Eubacterium_ruminantium_group
Melainabacteria
Gastranaerophilales
Thiopseudomonas
Cyanobacteria
Ruminococcus_2
Marinilabiliaceae
Anaerostipes
Treponema_2
Spirochaetales
Spirochaetia
Spirochaetaceae
Spirochaetes
Bacilli
Agathobacter
Flavobacteriaceae
Ruminococcus_1
Flavobacteriales
Rhodospirillales
Alphaproteobacteria
Veillonellaceae
Selenomonadales
Negativicutes
Megamonas
Faecalibacterium
Roseburia
Gammaproteobacteria
Succinivibrio
Succinivibrionaceae
Aeromonadales
Proteobacteria
Lachnospiraceae
Clostridiales
Clostridia
Firmicutes
−4
−2
0
2
4
LDA SCORE (log 10)

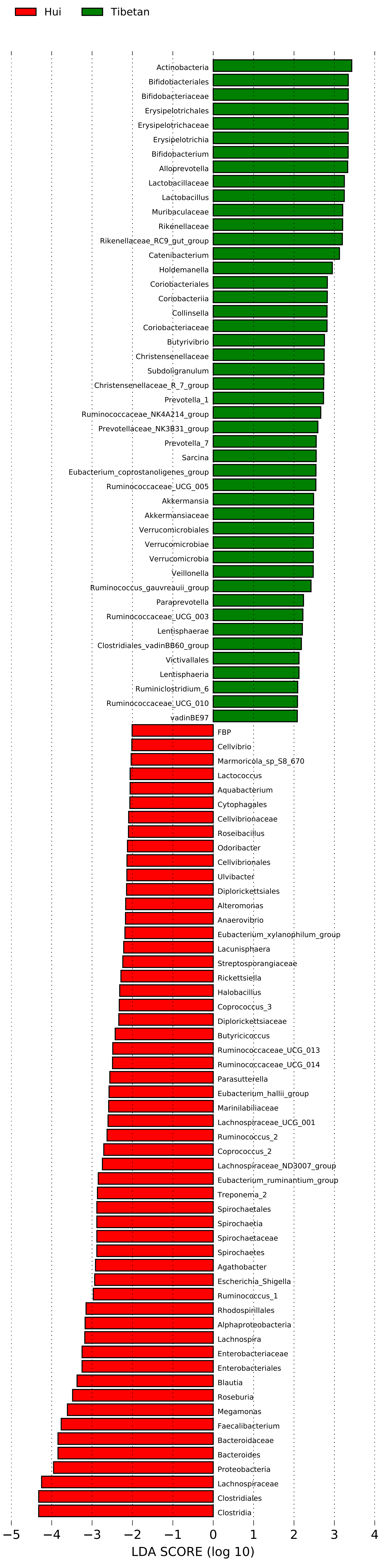

Hui
Tibetan
Actinobacteria
Bifidobacteriales
Bifidobacteriaceae
Erysipelotrichales
Erysipelotrichaceae
Erysipelotrichia
Bifidobacterium
Alloprevotella
Lactobacillaceae
Lactobacillus
Muribaculaceae
Rikenellaceae
Rikenellaceae_RC9_gut_group
Catenibacterium
Holdemanella
Coriobacteriales
Coriobacteriia
Collinsella
Coriobacteriaceae
Butyrivibrio
Christensenellaceae
Subdoligranulum
Christensenellaceae_R_7_group
Prevotella_1
Ruminococcaceae_NK4A214_group
Prevotellaceae_NK3B31_group
Prevotella_7
Sarcina
Eubacterium_coprostanoligenes_group
Ruminococcaceae_UCG_005
Akkermansia
Akkermansiaceae
Verrucomicrobiales
Verrucomicrobiae
Verrucomicrobia
Veillonella
Ruminococcus_gauvreauii_group
Paraprevotella
Ruminococcaceae_UCG_003
Lentisphaerae
Clostridiales_vadinBB60_group
Victivallales
Lentisphaeria
Ruminiclostridium_6
Ruminococcaceae_UCG_010
vadinBE97
FBP
Cellvibrio
Marmoricola_sp_S8_670
Lactococcus
Aquabacterium
Cytophagales
Cellvibrionaceae
Roseibacillus
Odoribacter
Cellvibrionales
Ulvibacter
Diplorickettsiales
Alteromonas
Anaerovibrio
Eubacterium_xylanophilum_group
Lacunisphaera
Streptosporangiaceae
Rickettsiella
Halobacillus
Coprococcus_3
Diplorickettsiaceae
Butyricicoccus
Ruminococcaceae_UCG_013
Ruminococcaceae_UCG_014
Parasutterella
Eubacterium_hallii_group
Marinilabiliaceae
Lachnospiraceae_UCG_001
Ruminococcus_2
Coprococcus_2
Lachnospiraceae_ND3007_group
Eubacterium_ruminantium_group
Treponema_2
Spirochaetales
Spirochaetia
Spirochaetaceae
Spirochaetes
Agathobacter
Escherichia_Shigella
Ruminococcus_1
Rhodospirillales
Alphaproteobacteria
Lachnospira
Enterobacteriaceae
Enterobacteriales
Blautia
Roseburia
Megamonas
Faecalibacterium
Bacteroidaceae
Bacteroides
Proteobacteria
Lachnospiraceae
Clostridiales
Clostridia
−5
−4
−3
−2
−1
0
1
2
3
4
LDA SCORE (log 10)

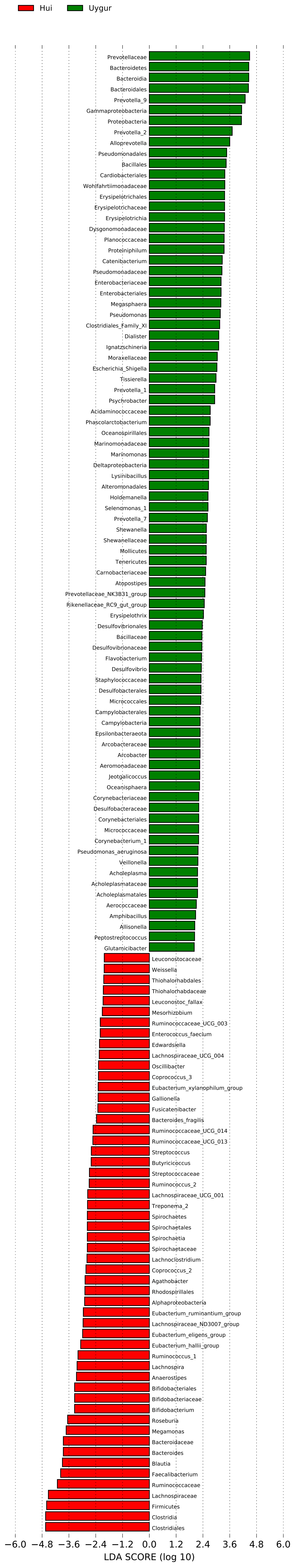

Hui
Uygur
Prevotellaceae
Bacteroidetes
Bacteroidia
Bacteroidales
Prevotella_9
Gammaproteobacteria
Proteobacteria
Prevotella_2
Alloprevotella
Pseudomonadales
Bacillales
Cardiobacteriales
Wohlfahrtiimonadaceae
Erysipelotrichales
Erysipelotrichaceae
Erysipelotrichia
Dysgonomonadaceae
Planococcaceae
Proteiniphilum
Catenibacterium
Pseudomonadaceae
Enterobacteriaceae
Enterobacteriales
Megasphaera
Pseudomonas
Clostridiales_Family_XI
Dialister
Ignatzschineria
Moraxellaceae
Escherichia_Shigella
Tissierella
Prevotella_1
Psychrobacter
Acidaminococcaceae
Phascolarctobacterium
Oceanospirillales
Marinomonadaceae
Marinomonas
Deltaproteobacteria
Lysinibacillus
Alteromonadales
Holdemanella
Selenomonas_1
Prevotella_7
Shewanella
Shewanellaceae
Mollicutes
Tenericutes
Carnobacteriaceae
Atopostipes
Prevotellaceae_NK3B31_group
Rikenellaceae_RC9_gut_group
Erysipelothrix
Desulfovibrionales
Bacillaceae
Desulfovibrionaceae
Flavobacterium
Desulfovibrio
Staphylococcaceae
Desulfobacterales
Micrococcales
Campylobacterales
Campylobacteria
Epsilonbacteraeota
Arcobacteraceae
Arcobacter
Aeromonadaceae
Jeotgalicoccus
Oceanisphaera
Corynebacteriaceae
Desulfobacteraceae
Corynebacteriales
Micrococcaceae
Corynebacterium_1
Pseudomonas_aeruginosa
Veillonella
Acholeplasma
Acholeplasmataceae
Acholeplasmatales
Aerococcaceae
Amphibacillus
Allisonella
Peptostreptococcus
Glutamicibacter
Leuconostocaceae
Weissella
Thiohalorhabdales
Thiohalorhabdaceae
Leuconostoc_fallax
Mesorhizobium
Ruminococcaceae_UCG_003
Enterococcus_faecium
Edwardsiella
Lachnospiraceae_UCG_004
Oscillibacter
Coprococcus_3
Eubacterium_xylanophilum_group
Gallionella
Fusicatenibacter
Bacteroides_fragilis
Ruminococcaceae_UCG_014
Ruminococcaceae_UCG_013
Streptococcus
Butyricicoccus
Streptococcaceae
Ruminococcus_2
Lachnospiraceae_UCG_001
Treponema_2
Spirochaetes
Spirochaetales
Spirochaetia
Spirochaetaceae
Lachnoclostridium
Coprococcus_2
Agathobacter
Rhodospirillales
Alphaproteobacteria
Eubacterium_ruminantium_group
Lachnospiraceae_ND3007_group
Eubacterium_eligens_group
Eubacterium_hallii_group
Ruminococcus_1
Lachnospira
Anaerostipes
Bifidobacteriales
Bifidobacteriaceae
Bifidobacterium
Roseburia
Megamonas
Bacteroidaceae
Bacteroides
Blautia
Faecalibacterium
Ruminococcaceae
Lachnospiraceae
Firmicutes
Clostridia
Clostridiales
−6.0
−4.8
−3.6
−2.4
−1.2
0.0
1.2
2.4
3.6
4.8
6.0
LDA SCORE (log 10)

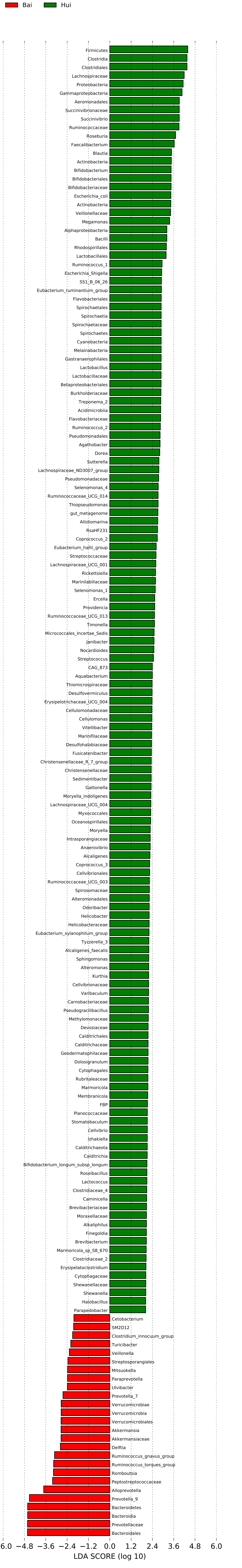
Bai
Hui
Firmicutes
Clostridia
Clostridiales
Lachnospiraceae
Proteobacteria
Gammaproteobacteria
Aeromonadales
Succinivibrionaceae
Succinivibrio
Ruminococcaceae
Roseburia
Faecalibacterium
Blautia
Actinobacteria
Bifidobacterium
Bifidobacteriales
Bifidobacteriaceae
Escherichia_coli
Actinobacteria
Veillonellaceae
Megamonas
Alphaproteobacteria
Bacilli
Rhodospirillales
Lactobacillales
Ruminococcus_1
Escherichia_Shigella
SS1_B_06_26
Eubacterium_ruminantium_group
Flavobacteriales
Spirochaetales
Spirochaetia
Spirochaetaceae
Spirochaetes
Cyanobacteria
Melainabacteria
Gastranaerophilales
Lactobacillus
Lactobacillaceae
Betaproteobacteriales
Burkholderiaceae
Treponema_2
Acidimicrobiia
Flavobacteriaceae
Ruminococcus_2
Pseudomonadales
Agathobacter
Dorea
Sutterella
Lachnospiraceae_ND3007_group
Pseudomonadaceae
Selenomonas_4
Ruminococcaceae_UCG_014
Thiopseudomonas
gut_metagenome
Aliidiomarina
RsaHF231
Coprococcus_2
Eubacterium_hallii_group
Streptococcaceae
Lachnospiraceae_UCG_001
Rickettsiella
Marinilabiliaceae
Selenomonas_1
Ercella
Providencia
Ruminococcaceae_UCG_013
Timonella
Micrococcales_Incertae_Sedis
Janibacter
Nocardioides
Streptococcus
CAG_873
Aquabacterium
Thiomicrospiraceae
Desulfovermiculus
Erysipelotrichaceae_UCG_004
Cellulomonadaceae
Cellulomonas
Vitellibacter
Marinifilaceae
Desulfohalobiaceae
Fusicatenibacter
Christensenellaceae_R_7_group
Christensenellaceae
Sedimentibacter
Gallionella
Moryella_indoligenes
Lachnospiraceae_UCG_004
Myxococcales
Oceanospirillales
Moryella
Intrasporangiaceae
Anaerovibrio
Alcaligenes
Coprococcus_3
Cellvibrionales
Ruminococcaceae_UCG_003
Spirosomaceae
Alteromonadales
Odoribacter
Helicobacter
Helicobacteraceae
Eubacterium_xylanophilum_group
Tyzzerella_3
Alcaligenes_faecalis
Sphingomonas
Alteromonas
Kurthia
Cellvibrionaceae
Variibaculum
Carnobacteriaceae
Pseudogracilibacillus
Methylomonaceae
Devosiaceae
Calditrichales
Calditrichaceae
Geodermatophilaceae
Dolosigranulum
Cytophagales
Rubritaleaceae
Marmoricola
Membranicola
FBP
Planococcaceae
Stomatobaculum
Cellvibrio
Izhakiella
Calditrichaeota
Calditrichia
Bifidobacterium_longum_subsp_longum
Roseibacillus
Lactococcus
Clostridiaceae_4
Caminicella
Brevibacteriaceae
Moraxellaceae
Alkaliphilus
Finegoldia
Brevibacterium
Marmoricola_sp_S8_670
Clostridiaceae_2
Erysipelatoclostridium
Cytophagaceae
Shewanellaceae
Shewanella
Halobacillus
Parapedobacter
Cetobacterium
SM2D12
Clostridium_innocuum_group
Turicibacter
Veillonella
Streptosporangiales
Mitsuokella
Paraprevotella
Ulvibacter
Prevotella_7
Verrucomicrobiae
Verrucomicrobia
Verrucomicrobiales
Akkermansia
Akkermansiaceae
Delftia
Ruminococcus_gnavus_group
Ruminococcus_torques_group
Romboutsia
Peptostreptococcaceae
Alloprevotella
Prevotella_9
Bacteroidetes
Bacteroidia
Prevotellaceae
Bacteroidales
−6.0
−4.8
−3.6
−2.4
−1.2
0.0
1.2
2.4
3.6
4.8
6.0
LDA SCORE (log 10)

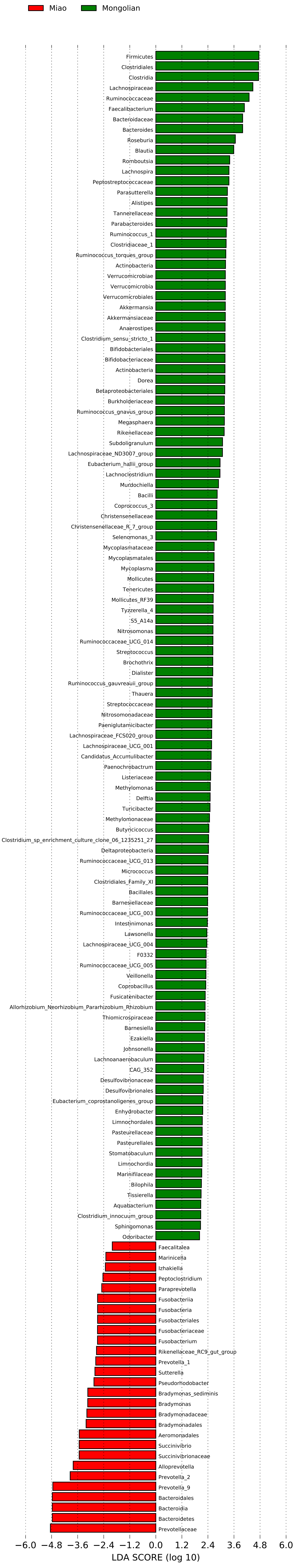
Miao
Mongolian
Firmicutes
Clostridiales
Clostridia
Lachnospiraceae
Ruminococcaceae
Faecalibacterium
Bacteroidaceae
Bacteroides
Roseburia
Blautia
Romboutsia
Lachnospira
Peptostreptococcaceae
Parasutterella
Alistipes
Tannerellaceae
Parabacteroides
Ruminococcus_1
Clostridiaceae_1
Ruminococcus_torques_group
Actinobacteria
Verrucomicrobiae
Verrucomicrobia
Verrucomicrobiales
Akkermansia
Akkermansiaceae
Anaerostipes
Clostridium_sensu_stricto_1
Bifidobacteriales
Bifidobacteriaceae
Actinobacteria
Dorea
Betaproteobacteriales
Burkholderiaceae
Ruminococcus_gnavus_group
Megasphaera
Rikenellaceae
Subdoligranulum
Lachnospiraceae_ND3007_group
Eubacterium_hallii_group
Lachnoclostridium
Murdochiella
Bacilli
Coprococcus_3
Christensenellaceae
Christensenellaceae_R_7_group
Selenomonas_3
Mycoplasmataceae
Mycoplasmatales
Mycoplasma
Mollicutes
Tenericutes
Mollicutes_RF39
Tyzzerella_4
S5_A14a
Nitrosomonas
Ruminococcaceae_UCG_014
Streptococcus
Brochothrix
Dialister
Ruminococcus_gauvreauii_group
Thauera
Streptococcaceae
Nitrosomonadaceae
Paeniglutamicibacter
Lachnospiraceae_FCS020_group
Lachnospiraceae_UCG_001
Candidatus_Accumulibacter
Paenochrobactrum
Listeriaceae
Methylomonas
Delftia
Turicibacter
Methylomonaceae
Butyricicoccus
Clostridium_sp_enrichment_culture_clone_06_1235251_27
Deltaproteobacteria
Ruminococcaceae_UCG_013
Micrococcus
Clostridiales_Family_XI
Bacillales
Barnesiellaceae
Ruminococcaceae_UCG_003
Intestinimonas
Lawsonella
Lachnospiraceae_UCG_004
F0332
Ruminococcaceae_UCG_005
Veillonella
Coprobacillus
Fusicatenibacter
Allorhizobium_Neorhizobium_Pararhizobium_Rhizobium
Thiomicrospiraceae
Barnesiella
Ezakiella
Johnsonella
Lachnoanaerobaculum
CAG_352
Desulfovibrionaceae
Desulfovibrionales
Eubacterium_coprostanoligenes_group
Enhydrobacter
Limnochordales
Pasteurellaceae
Pasteurellales
Stomatobaculum
Limnochordia
Marinifilaceae
Bilophila
Tissierella
Aquabacterium
Clostridium_innocuum_group
Sphingomonas
Odoribacter
Faecalitalea
Marinicella
Izhakiella
Peptoclostridium
Paraprevotella
Fusobacteriia
Fusobacteria
Fusobacteriales
Fusobacteriaceae
Fusobacterium
Rikenellaceae_RC9_gut_group
Prevotella_1
Sutterella
Pseudorhodobacter
Bradymonas_sediminis
Bradymonas
Bradymonadaceae
Bradymonadales
Aeromonadales
Succinivibrio
Succinivibrionaceae
Alloprevotella
Prevotella_2
Prevotella_9
Bacteroidales
Bacteroidia
Bacteroidetes
Prevotellaceae
−6.0
−4.8
−3.6
−2.4
−1.2
0.0
1.2
2.4
3.6
4.8
6.0
LDA SCORE (log 10)

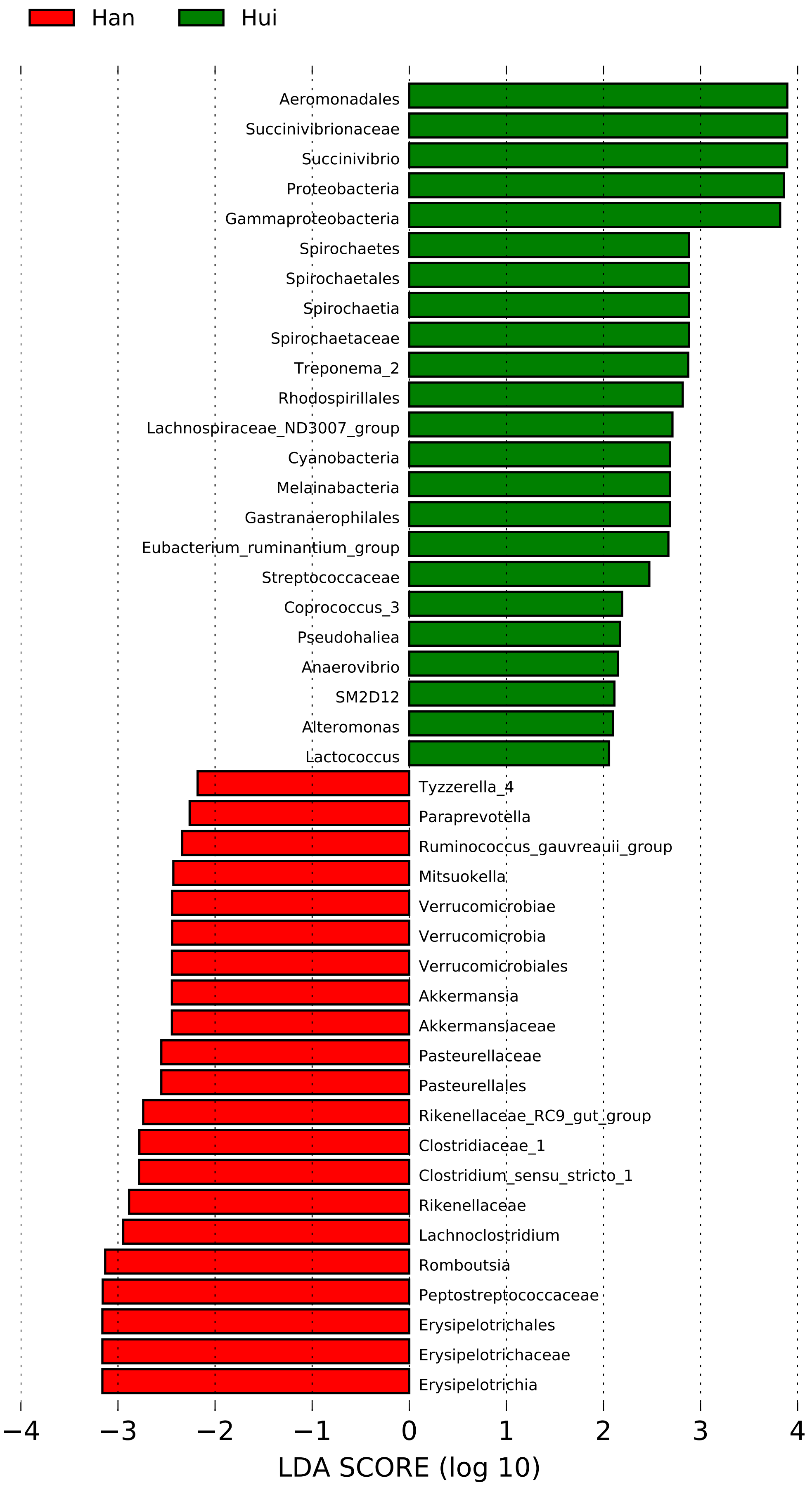
Han
Hui
Aeromonadales
Succinivibrionaceae
Succinivibrio
Proteobacteria
Gammaproteobacteria
Spirochaetes
Spirochaetales
Spirochaetia
Spirochaetaceae
Treponema_2
Rhodospirillales
Lachnospiraceae_ND3007_group
Cyanobacteria
Melainabacteria
Gastranaerophilales
Eubacterium_ruminantium_group
Streptococcaceae
Coprococcus_3
Pseudohaliea
Anaerovibrio
SM2D12
Alteromonas
Lactococcus
Tyzzerella_4
Paraprevotella
Ruminococcus_gauvreauii_group
Mitsuokella
Verrucomicrobiae
Verrucomicrobia
Verrucomicrobiales
Akkermansia
Akkermansiaceae
Pasteurellaceae
Pasteurellales
Rikenellaceae_RC9_gut_group
Clostridiaceae_1
Clostridium_sensu_stricto_1
Rikenellaceae
Lachnoclostridium
Romboutsia
Peptostreptococcaceae
Erysipelotrichales
Erysipelotrichaceae
Erysipelotrichia
−4
−3
−2
−1
0
1
2
3
4
LDA SCORE (log 10)

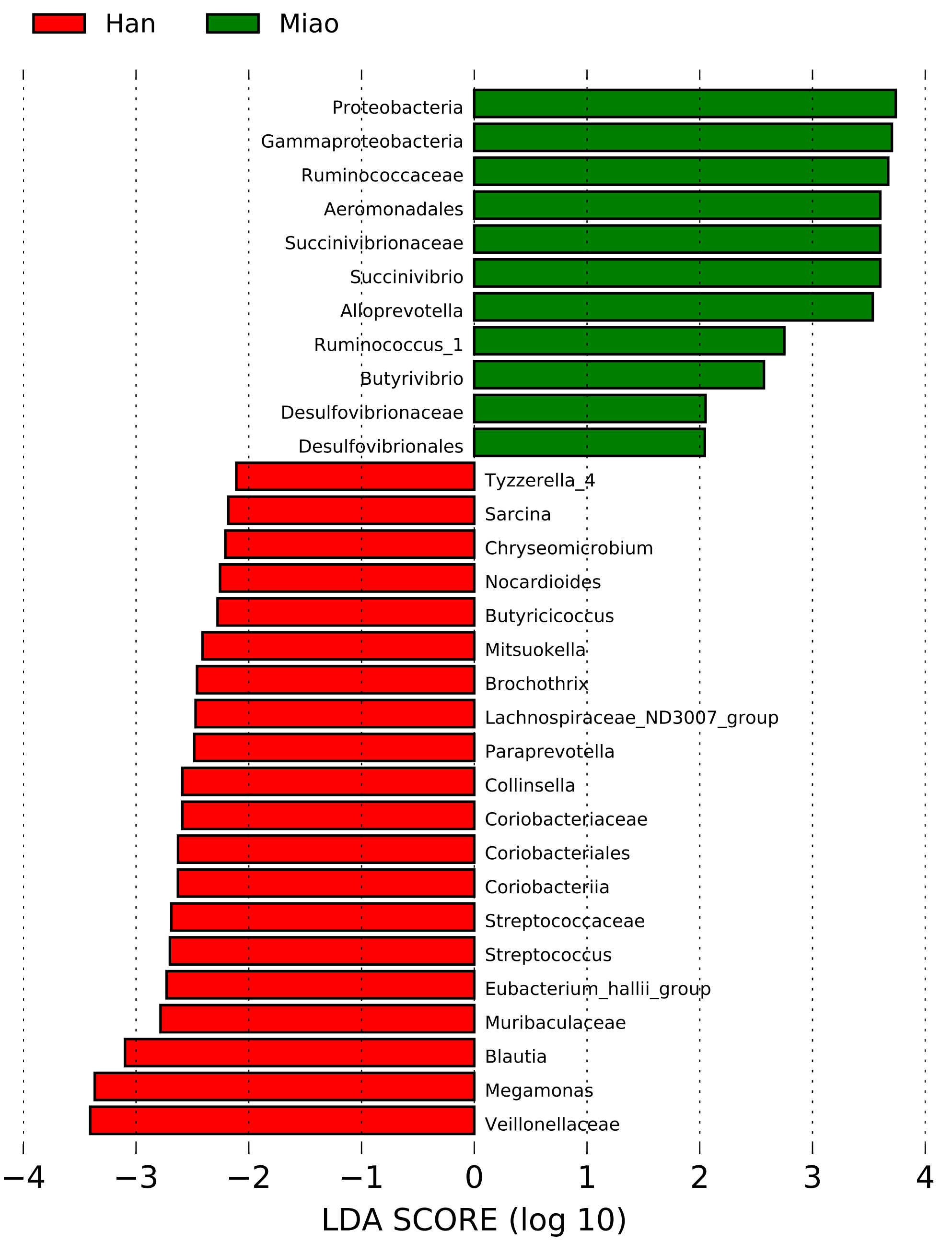

Han
Miao
Proteobacteria
Gammaproteobacteria
Ruminococcaceae
Aeromonadales
Succinivibrionaceae
Succinivibrio
Alloprevotella
Ruminococcus_1
Butyrivibrio
Desulfovibrionaceae
Desulfovibrionales
Tyzzerella_4
Sarcina
Chryseomicrobium
Nocardioides
Butyricicoccus
Mitsuokella
Brochothrix
Lachnospiraceae_ND3007_group
Paraprevotella
Collinsella
Coriobacteriaceae
Coriobacteriales
Coriobacteriia
Streptococcaceae
Streptococcus
Eubacterium_hallii_group
Muribaculaceae
Blautia
Megamonas
Veillonellaceae
−4
−3
−2
−1
0
1
2
3
4
LDA SCORE (log 10)

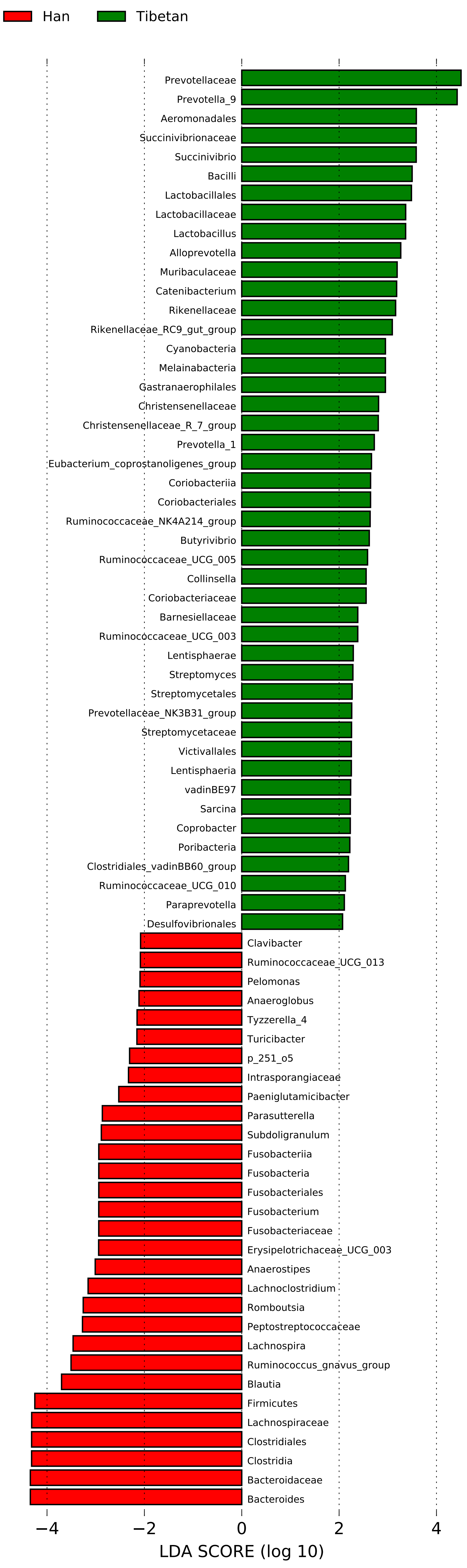

Han
Tibetan
Prevotellaceae
Prevotella_9
Aeromonadales
Succinivibrionaceae
Succinivibrio
Bacilli
Lactobacillales
Lactobacillaceae
Lactobacillus
Alloprevotella
Muribaculaceae
Catenibacterium
Rikenellaceae
Rikenellaceae_RC9_gut_group
Cyanobacteria
Melainabacteria
Gastranaerophilales
Christensenellaceae
Christensenellaceae_R_7_group
Prevotella_1
Eubacterium_coprostanoligenes_group
Coriobacteriia
Coriobacteriales
Ruminococcaceae_NK4A214_group
Butyrivibrio
Ruminococcaceae_UCG_005
Collinsella
Coriobacteriaceae
Barnesiellaceae
Ruminococcaceae_UCG_003
Lentisphaerae
Streptomyces
Streptomycetales
Prevotellaceae_NK3B31_group
Streptomycetaceae
Victivallales
Lentisphaeria
vadinBE97
Sarcina
Coprobacter
Poribacteria
Clostridiales_vadinBB60_group
Ruminococcaceae_UCG_010
Paraprevotella
Desulfovibrionales
Clavibacter
Ruminococcaceae_UCG_013
Pelomonas
Anaeroglobus
Tyzzerella_4
Turicibacter
p_251_o5
Intrasporangiaceae
Paeniglutamicibacter
Parasutterella
Subdoligranulum
Fusobacteriia
Fusobacteria
Fusobacteriales
Fusobacterium
Fusobacteriaceae
Erysipelotrichaceae_UCG_003
Anaerostipes
Lachnoclostridium
Romboutsia
Peptostreptococcaceae
Lachnospira
Ruminococcus_gnavus_group
Blautia
Firmicutes
Lachnospiraceae
Clostridiales
Clostridia
Bacteroidaceae
Bacteroides
−4
−2
0
2
4
LDA SCORE (log 10)

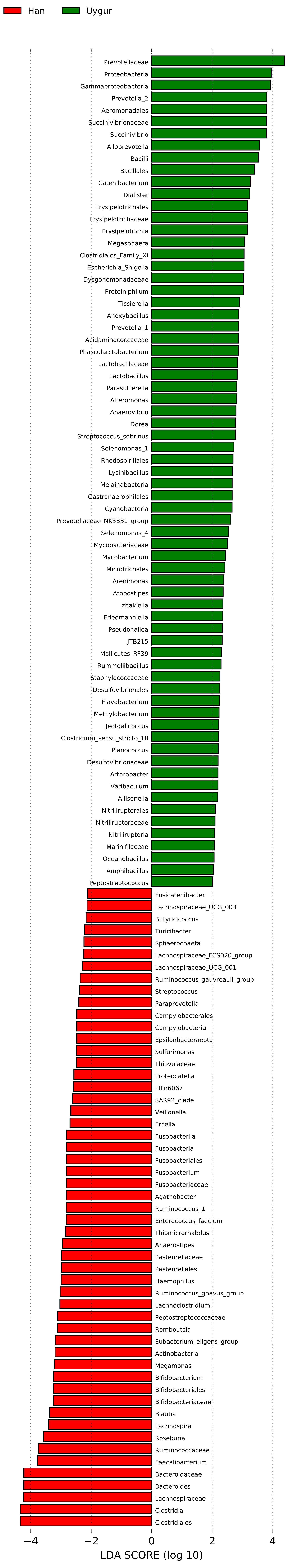
Han
Uygur
Prevotellaceae
Proteobacteria
Gammaproteobacteria
Prevotella_2
Aeromonadales
Succinivibrionaceae
Succinivibrio
Alloprevotella
Bacilli
Bacillales
Catenibacterium
Dialister
Erysipelotrichales
Erysipelotrichaceae
Erysipelotrichia
Megasphaera
Clostridiales_Family_XI
Escherichia_Shigella
Dysgonomonadaceae
Proteiniphilum
Tissierella
Anoxybacillus
Prevotella_1
Acidaminococcaceae
Phascolarctobacterium
Lactobacillaceae
Lactobacillus
Parasutterella
Alteromonas
Anaerovibrio
Dorea
Streptococcus_sobrinus
Selenomonas_1
Rhodospirillales
Lysinibacillus
Melainabacteria
Gastranaerophilales
Cyanobacteria
Prevotellaceae_NK3B31_group
Selenomonas_4
Mycobacteriaceae
Mycobacterium
Microtrichales
Arenimonas
Atopostipes
Izhakiella
Friedmanniella
Pseudohaliea
JTB215
Mollicutes_RF39
Rummeliibacillus
Staphylococcaceae
Desulfovibrionales
Flavobacterium
Methylobacterium
Jeotgalicoccus
Clostridium_sensu_stricto_18
Planococcus
Desulfovibrionaceae
Arthrobacter
Varibaculum
Allisonella
Nitriliruptorales
Nitriliruptoraceae
Nitriliruptoria
Marinifilaceae
Oceanobacillus
Amphibacillus
Peptostreptococcus
Fusicatenibacter
Lachnospiraceae_UCG_003
Butyricicoccus
Turicibacter
Sphaerochaeta
Lachnospiraceae_FCS020_group
Lachnospiraceae_UCG_001
Ruminococcus_gauvreauii_group
Streptococcus
Paraprevotella
Campylobacterales
Campylobacteria
Epsilonbacteraeota
Sulfurimonas
Thiovulaceae
Proteocatella
Ellin6067
SAR92_clade
Veillonella
Ercella
Fusobacteriia
Fusobacteria
Fusobacteriales
Fusobacterium
Fusobacteriaceae
Agathobacter
Ruminococcus_1
Enterococcus_faecium
Thiomicrorhabdus
Anaerostipes
Pasteurellaceae
Pasteurellales
Haemophilus
Ruminococcus_gnavus_group
Lachnoclostridium
Peptostreptococcaceae
Romboutsia
Eubacterium_eligens_group
Actinobacteria
Megamonas
Bifidobacterium
Bifidobacteriales
Bifidobacteriaceae
Blautia
Lachnospira
Roseburia
Ruminococcaceae
Faecalibacterium
Bacteroidaceae
Bacteroides
Lachnospiraceae
Clostridia
Clostridiales
−4
−2
0
2
4
LDA SCORE (log 10)

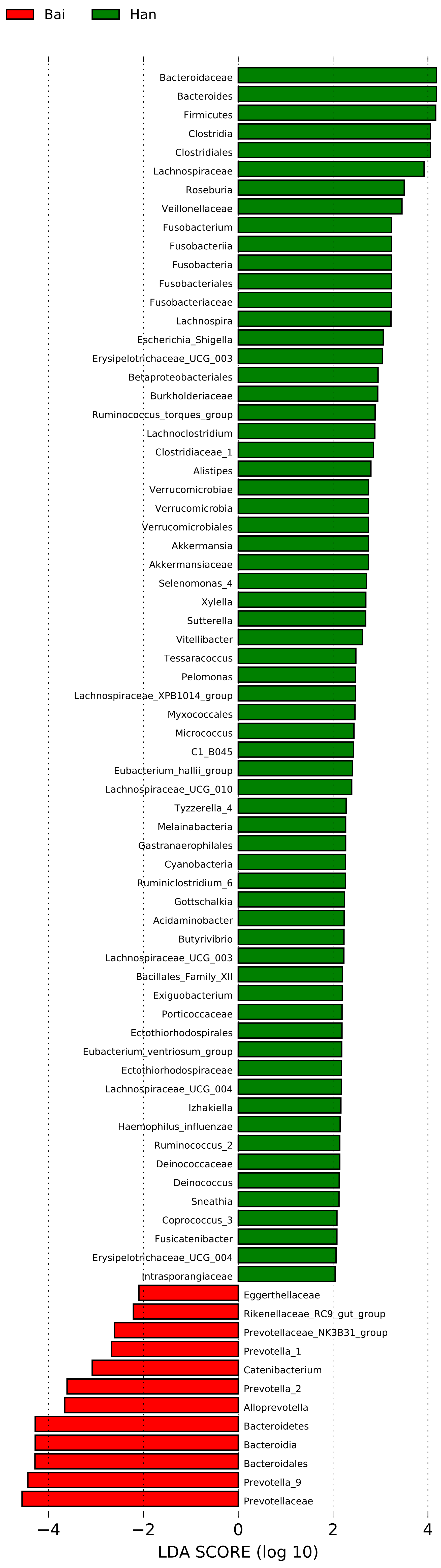
Bai
Han
Bacteroidaceae
Bacteroides
Firmicutes
Clostridia
Clostridiales
Lachnospiraceae
Roseburia
Veillonellaceae
Fusobacterium
Fusobacteriia
Fusobacteria
Fusobacteriales
Fusobacteriaceae
Lachnospira
Escherichia_Shigella
Erysipelotrichaceae_UCG_003
Betaproteobacteriales
Burkholderiaceae
Ruminococcus_torques_group
Lachnoclostridium
Clostridiaceae_1
Alistipes
Verrucomicrobiae
Verrucomicrobia
Verrucomicrobiales
Akkermansia
Akkermansiaceae
Selenomonas_4
Xylella
Sutterella
Vitellibacter
Tessaracoccus
Pelomonas
Lachnospiraceae_XPB1014_group
Myxococcales
Micrococcus
C1_B045
Eubacterium_hallii_group
Lachnospiraceae_UCG_010
Tyzzerella_4
Melainabacteria
Gastranaerophilales
Cyanobacteria
Ruminiclostridium_6
Gottschalkia
Acidaminobacter
Butyrivibrio
Lachnospiraceae_UCG_003
Bacillales_Family_XII
Exiguobacterium
Porticoccaceae
Ectothiorhodospirales
Eubacterium_ventriosum_group
Ectothiorhodospiraceae
Lachnospiraceae_UCG_004
Izhakiella
Haemophilus_influenzae
Ruminococcus_2
Deinococcaceae
Deinococcus
Sneathia
Coprococcus_3
Fusicatenibacter
Erysipelotrichaceae_UCG_004
Intrasporangiaceae
Eggerthellaceae
Rikenellaceae_RC9_gut_group
Prevotellaceae_NK3B31_group
Prevotella_1
Catenibacterium
Prevotella_2
Alloprevotella
Bacteroidetes
Bacteroidia
Bacteroidales
Prevotella_9
Prevotellaceae
−4
−2
0
2
4
LDA SCORE (log 10)

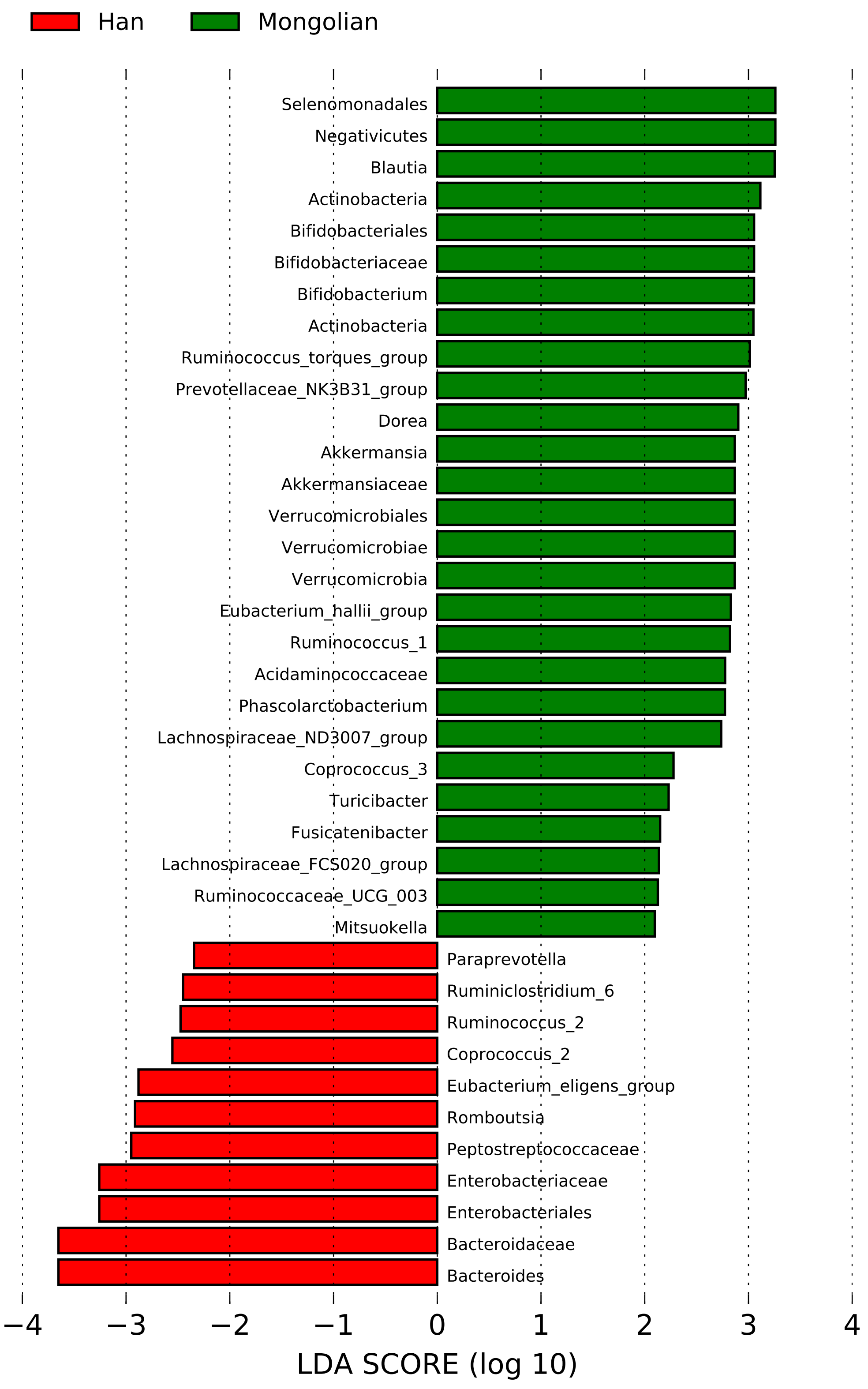

Han
Mongolian
Selenomonadales
Negativicutes
Blautia
Actinobacteria
Bifidobacteriales
Bifidobacteriaceae
Bifidobacterium
Actinobacteria
Ruminococcus_torques_group
Prevotellaceae_NK3B31_group
Dorea
Akkermansia
Akkermansiaceae
Verrucomicrobiales
Verrucomicrobiae
Verrucomicrobia
Eubacterium_hallii_group
Ruminococcus_1
Acidaminococcaceae
Phascolarctobacterium
Lachnospiraceae_ND3007_group
Coprococcus_3
Turicibacter
Fusicatenibacter
Lachnospiraceae_FCS020_group
Ruminococcaceae_UCG_003
Mitsuokella
Paraprevotella
Ruminiclostridium_6
Ruminococcus_2
Coprococcus_2
Eubacterium_eligens_group
Romboutsia
Peptostreptococcaceae
Enterobacteriaceae
Enterobacteriales
Bacteroidaceae
Bacteroides
−4
−3
−2
−1
0
1
2
3
4
LDA SCORE (log 10)

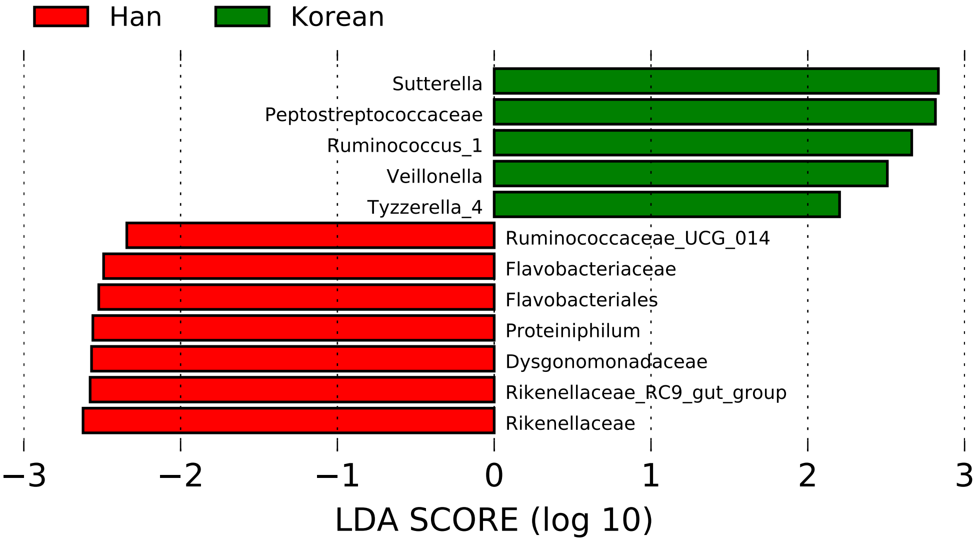
Han
Korean
Sutterella
Peptostreptococcaceae
Ruminococcus_1
Veillonella
Tyzzerella_4
Ruminococcaceae_UCG_014
Flavobacteriaceae
Flavobacteriales
Proteiniphilum
Dysgonomonadaceae
Rikenellaceae_RC9_gut_group
Rikenellaceae
−3
−2
−1
0
1
2
3
LDA SCORE (log 10)
