# Supplementary material for: The Core and Distinction of the Gut Microbiota in Chinese Populations across Geography and Ethnicity
Source: Microorganisms. 2020 Oct 14;8(10):1579. doi: 10.3390/microorganisms8101579 (PMC7602275; doi:10.3390/microorganisms8101579)
Supplement: Supplementary file 1 [file microorganisms-08-01579-s001.zip › major figures/Figure 4.pdf]

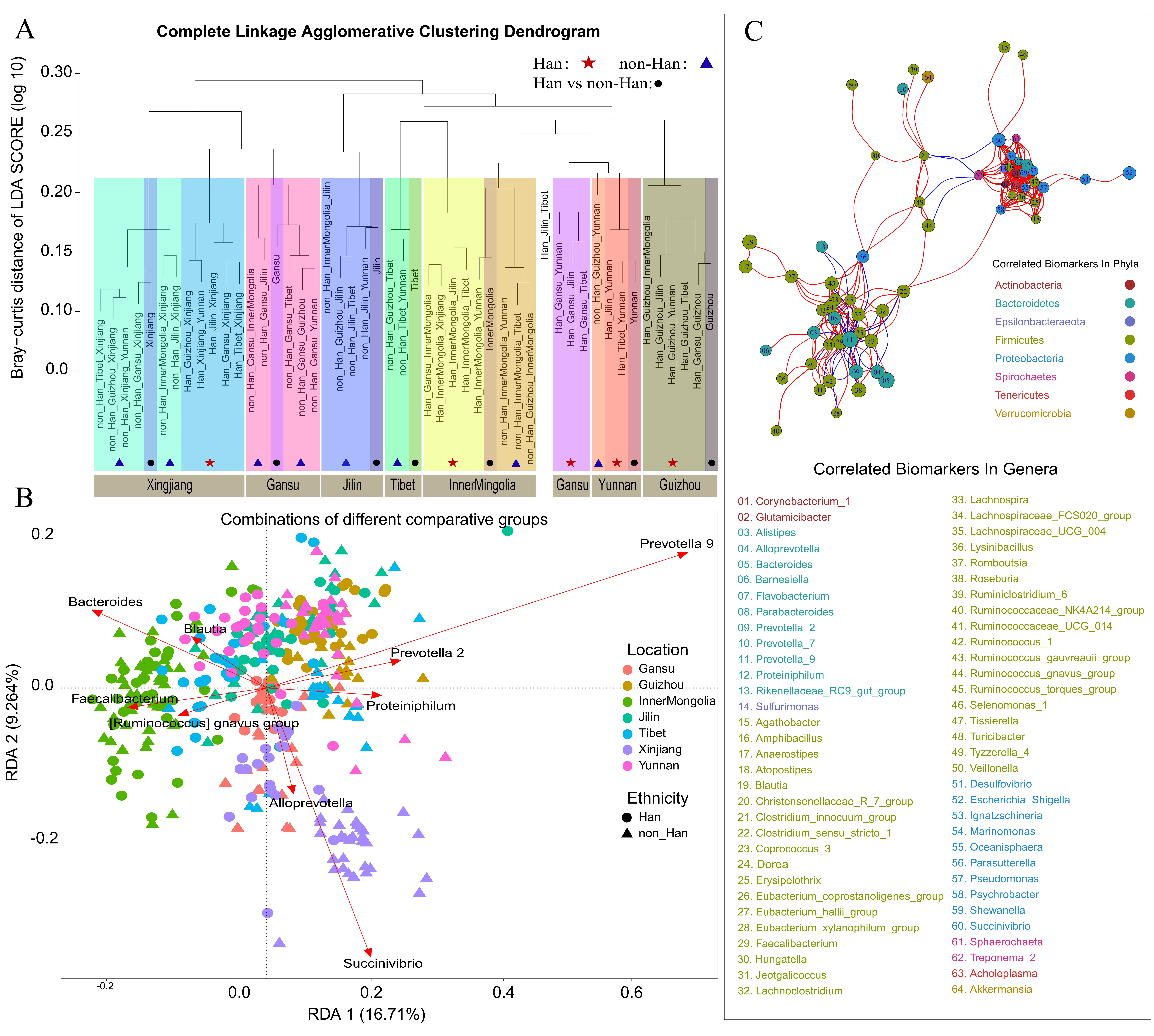

A
Complete Linkage Agglomerative Clustering Dendrogram
Han: ★ non-Han: ▲
Han vs non-Han: ●
Bray-curtis distance of LDA SCORE (log 10)
0.30
0.25
0.20
0.15
0.10
0.0
non_Han_Tibet_Xinjiang
non_Han_Guizhou_Xinjiang
non_Han_Xinjiang_Yunnan
non_Han_Gansu_Xinjiang
Xinjiang
non_Han_InnerMongolia_Xinjiang
non_Han_Jilin_Xinjiang
Han_Guizhou_Xinjiang
Han_Xinjiang_Yunnan
Han_Jilin_Xinjiang
Han_Gansu_Xinjiang
Han_Tibet_Xinjiang
non_Han_Gansu_InnerMongolia
non_Han_Gansu_Jilin
Gansu
non_Han_Gansu_Tibet
non_Han_Gansu_Guizhou
non_Han_Gansu_Yunnan
non_Han_InnerMongolia_Jilin
non_Han_Guizhou_Jilin
non_Han_Jilin_Tibet
non_Han_Jilin_Yunnan
Jilin
non_Han_Guizhou_Tibet
non_Han_Tibet_Yunnan
Tibet
Han_Gansu_InnerMongolia
Han_InnerMongolia_Xinjiang
Han_InnerMongolia_Jilin
Han_InnerMongolia_Tibet
Han_InnerMongolia_Yunnan
InnerMongolia
non_Han_InnerMongolia_Yunnan
non_Han_InnerMongolia_Tibet
non_Han_Guizhou_InnerMongolia
Han_Jilin_Tibet
Han_Gansu_Yunnan
Han_Gansu_Jilin
Han_Gansu_Tibet
non_Han_Guizhou_Yunnan
Han_Jilin_Yunnan
Han_Tibet_Yunnan
Yunnan
Han_Guizhou_InnerMongolia
Han_Guizhou_Jilin
Han_Guizhou_Yunnan
Han_Guizhou_Tibet
Han_Gansu_Guizhou
Guizhou
Xingjiang
Gansu
Jilin
Tibet
InnerMingolia
Gansu
Yunnan
Guizhou
B
Combinations of different comparative groups
Prevotella 9
Bacteroides
Blautia
Prevotella 2
Faecalibacterium
Proteiniphilum
[Ruminococcus] gnavus group
Alloprevotella
Succinivibrio
RDA 2 (9.264%)
RDA 1 (16.71%)
0.2
0.0
-0.2
-0.2
0.0
0.2
0.4
0.6
Location
Gansu
Guizhou
InnerMongolia
Jilin
Tibet
Xinjiang
Yunnan
Ethnicity
Han
non_Han
C
Correlated Biomarkers In Phyla
Actinobacteria
Bacteroidetes
Epsilonbacteraeota
Firmicutes
Proteobacteria
Spirochaetes
Tenericutes
Verrucomicrobia
Correlated Biomarkers In Genera
01. Corynebacterium_1
02. Glutamicibacter
03. Alistipes
04. Alloprevotella
05. Bacteroides
06. Barnesiella
07. Flavobacterium
08. Parabacteroides
09. Prevotella_2
10. Prevotella_7
11. Prevotella_9
12. Proteiniphilum
13. Rikenellaceae_RC9_gut_group
14. Sulfurimonas
15. Agathobacter
16. Amphibacillus
17. Anaerostipes
18. Atopostipes
19. Blautia
20. Christensenellaceae_R_7_group
21. Clostridium_innocuum_group
22. Clostridium_sensu_stricto_1
23. Coprococcus_3
24. Dorea
25. Erysipelothrix
26. Eubacterium_coprostanoligenes_group
27. Eubacterium_hallii_group
28. Eubacterium_xylanophilum_group
29. Faecalibacterium
30. Hungatella
31. Jeotgalicoccus
32. Lachnoclostridium
33. Lachnospira
34. Lachnospiraceae_FCS020_group
35. Lachnospiraceae_UCG_004
36. Lysinibacillus
37. Romboutsia
38. Roseburia
39. Ruminiclostridium_6
40. Ruminococcaceae_NK4A214_group
41. Ruminococcaceae_UCG_014
42. Ruminococcus_1
43. Ruminococcus_gauvreauii_group
44. Ruminococcus_gnavus_group
45. Ruminococcus_torques_group
46. Selenomonas_1
47. Tissierella
48. Turicibacter
49. Tyzzerella_4
50. Veillonella
51. Desulfovibrio
52. Escherichia_Shigella
53. Ignatzschineria
54. Marinomonas
55. Oceanisphaera
56. Parasutterella
57. Pseudomonas
58. Psychrobacter
59. Shewanella
60. Succinivibrio
61. Sphaerochaeta
62. Treponema_2
63. Acholeplasma
64. Akkermansia
