# Supplementary figures and images for: The Core and Distinction of the Gut Microbiota in Chinese Populations across Geography and Ethnicity
Source: Microorganisms. 2020 Oct 14;8(10):1579. doi: 10.3390/microorganisms8101579 (PMC7602275; doi:10.3390/microorganisms8101579)

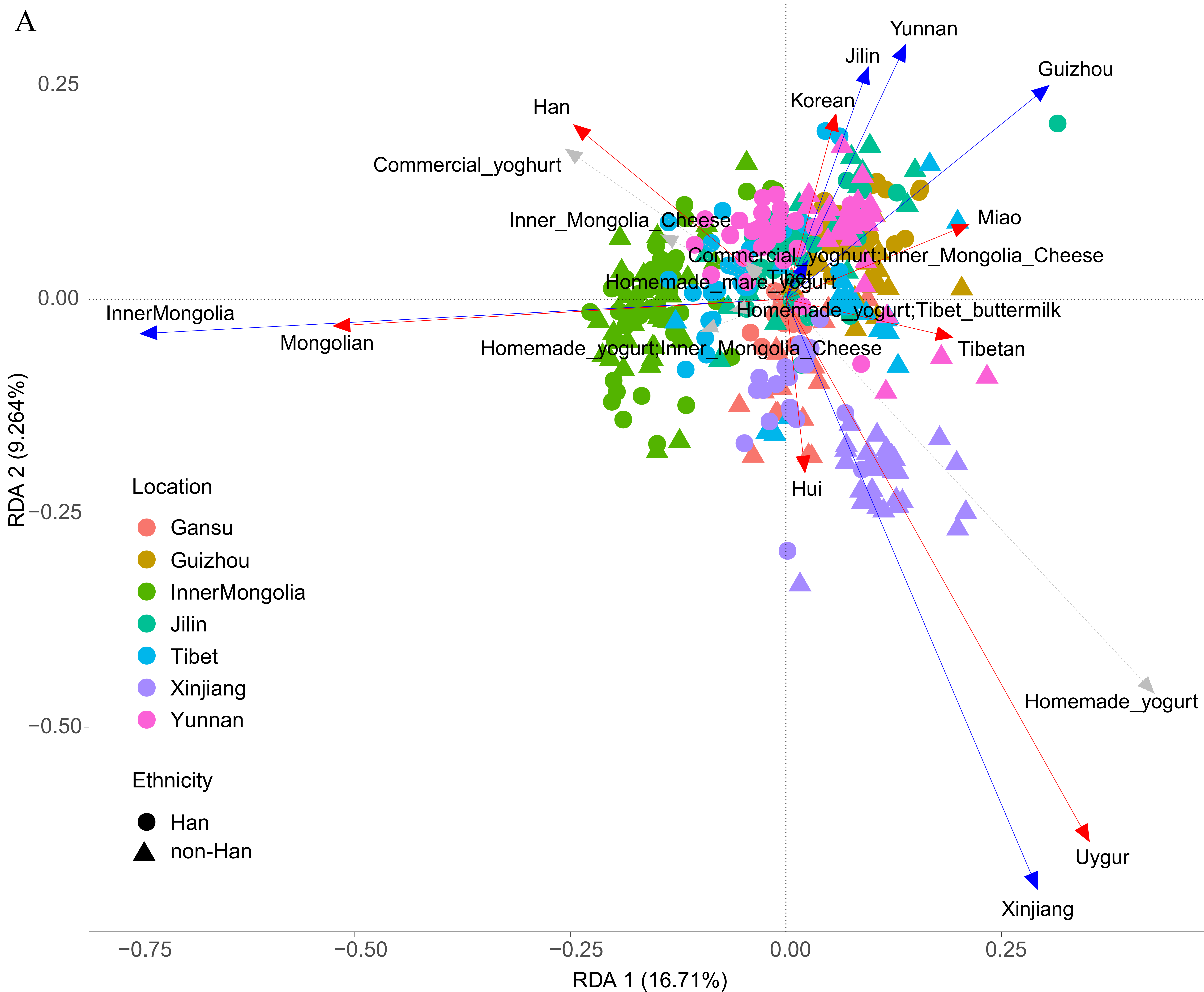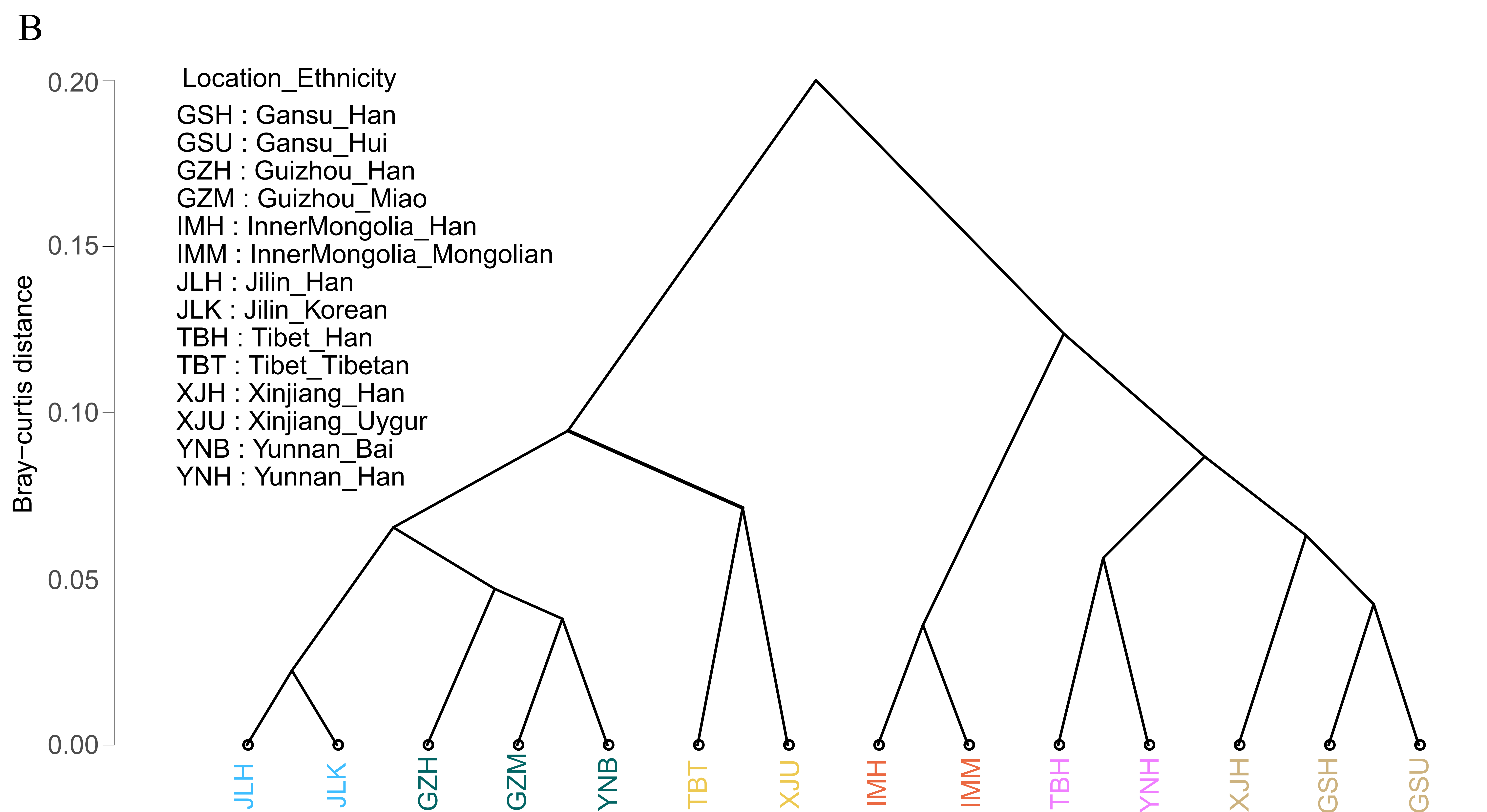

Supplement: Supplementary file 1 [file microorganisms-08-01579-s001.zip › major figures/Figure 1.pdf]

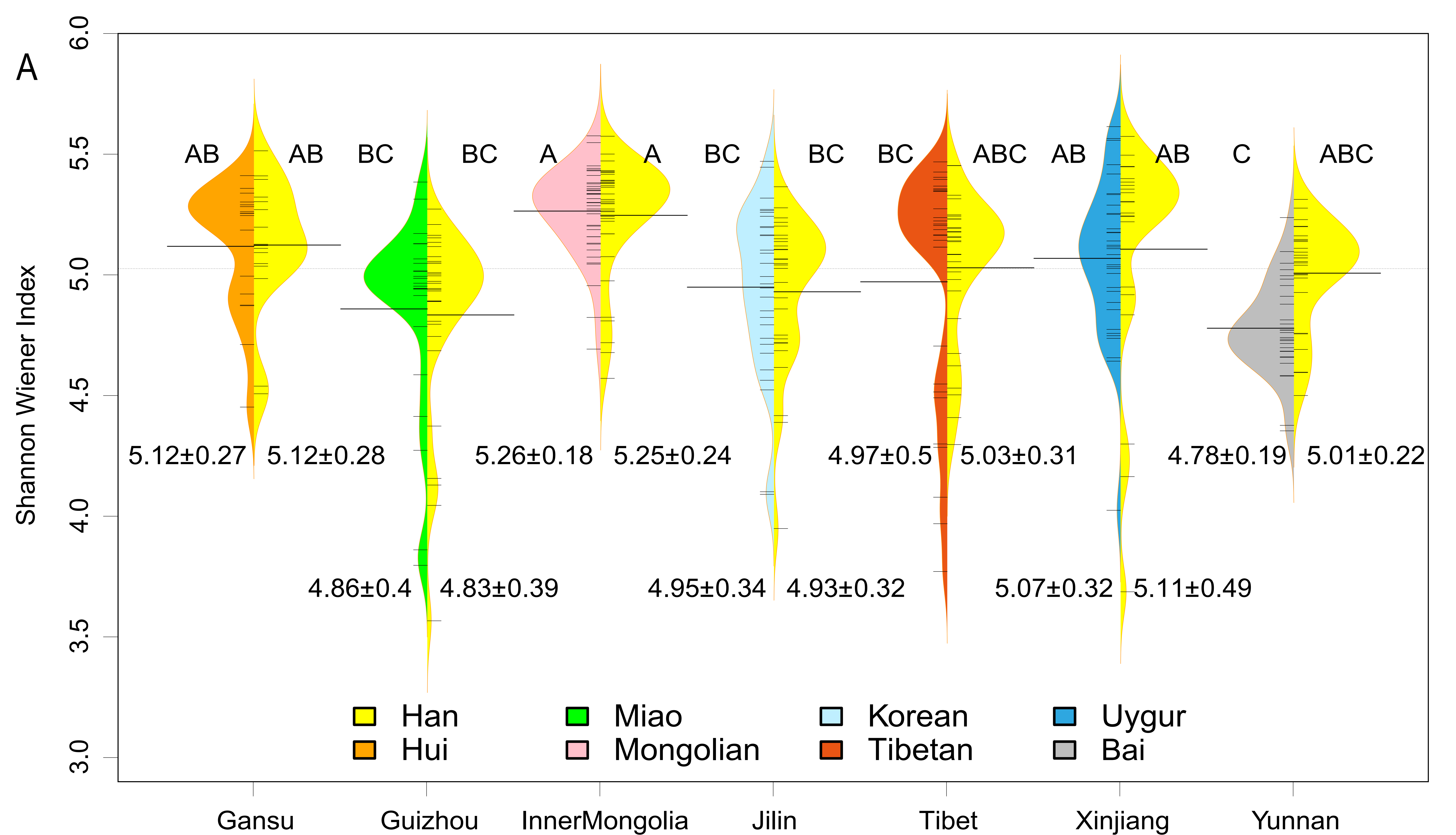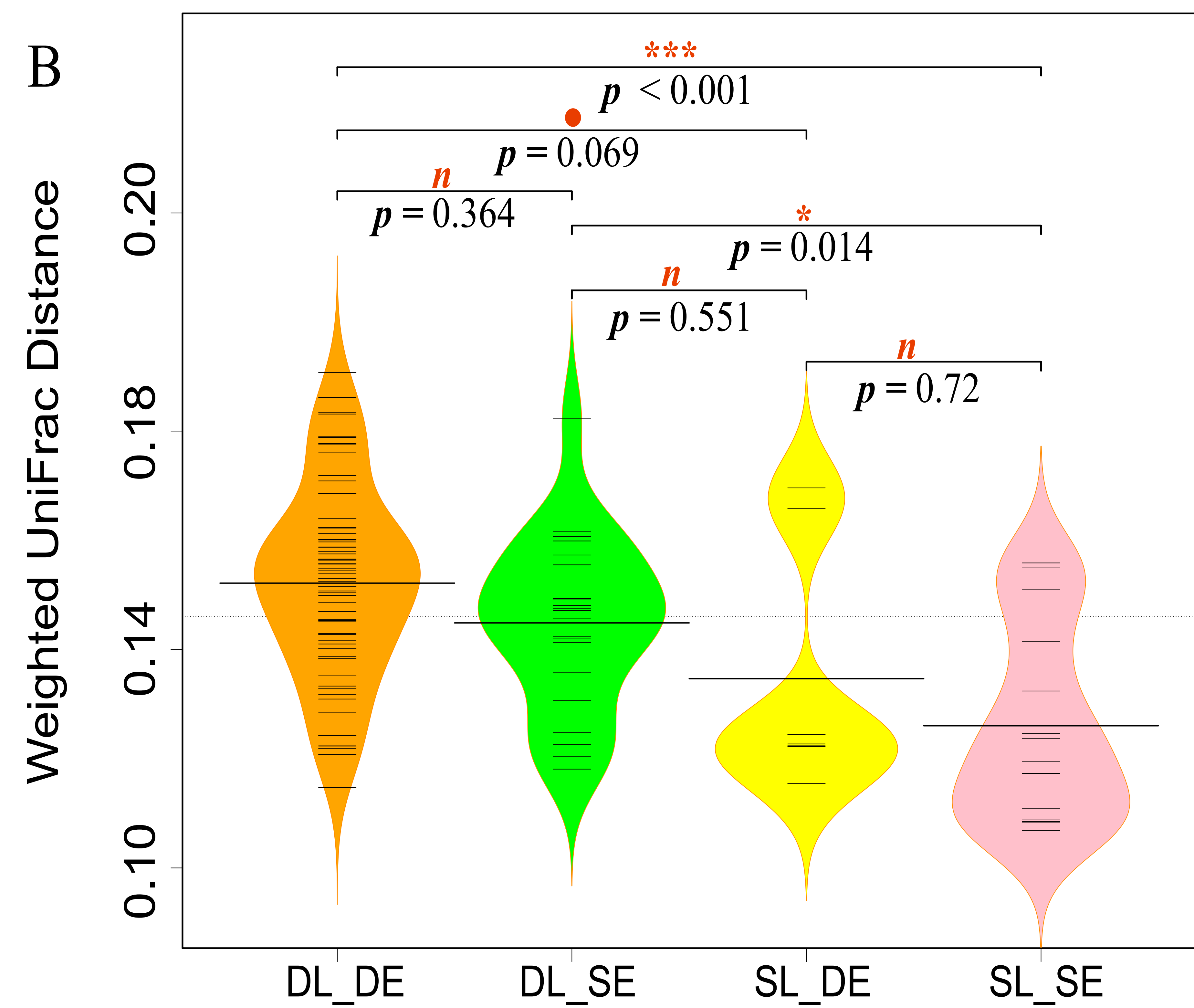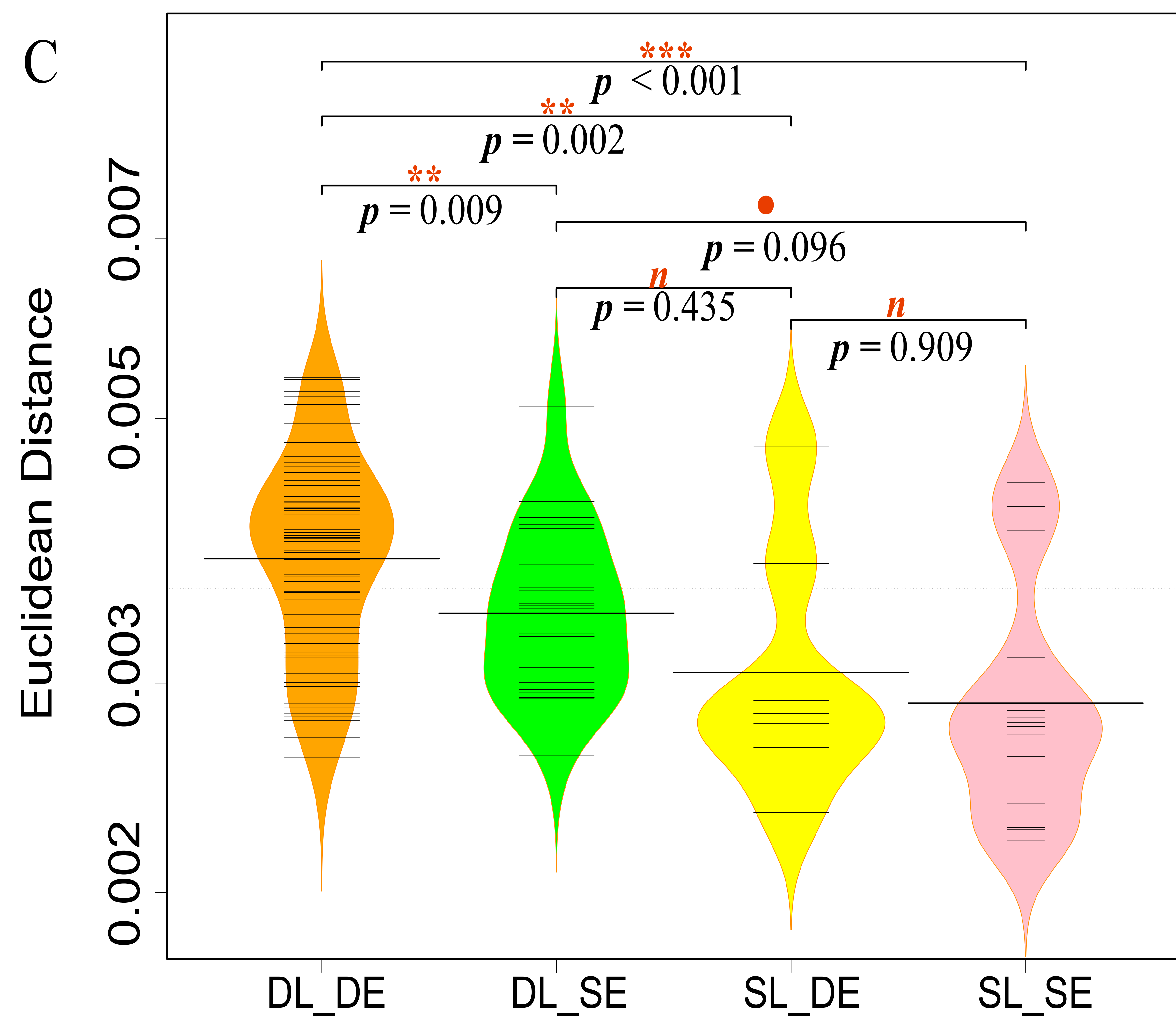

Supplement: Supplementary file 1 [file microorganisms-08-01579-s001.zip › major figures/Figure 2.pdf]

A

## Proportion of different enterotypes

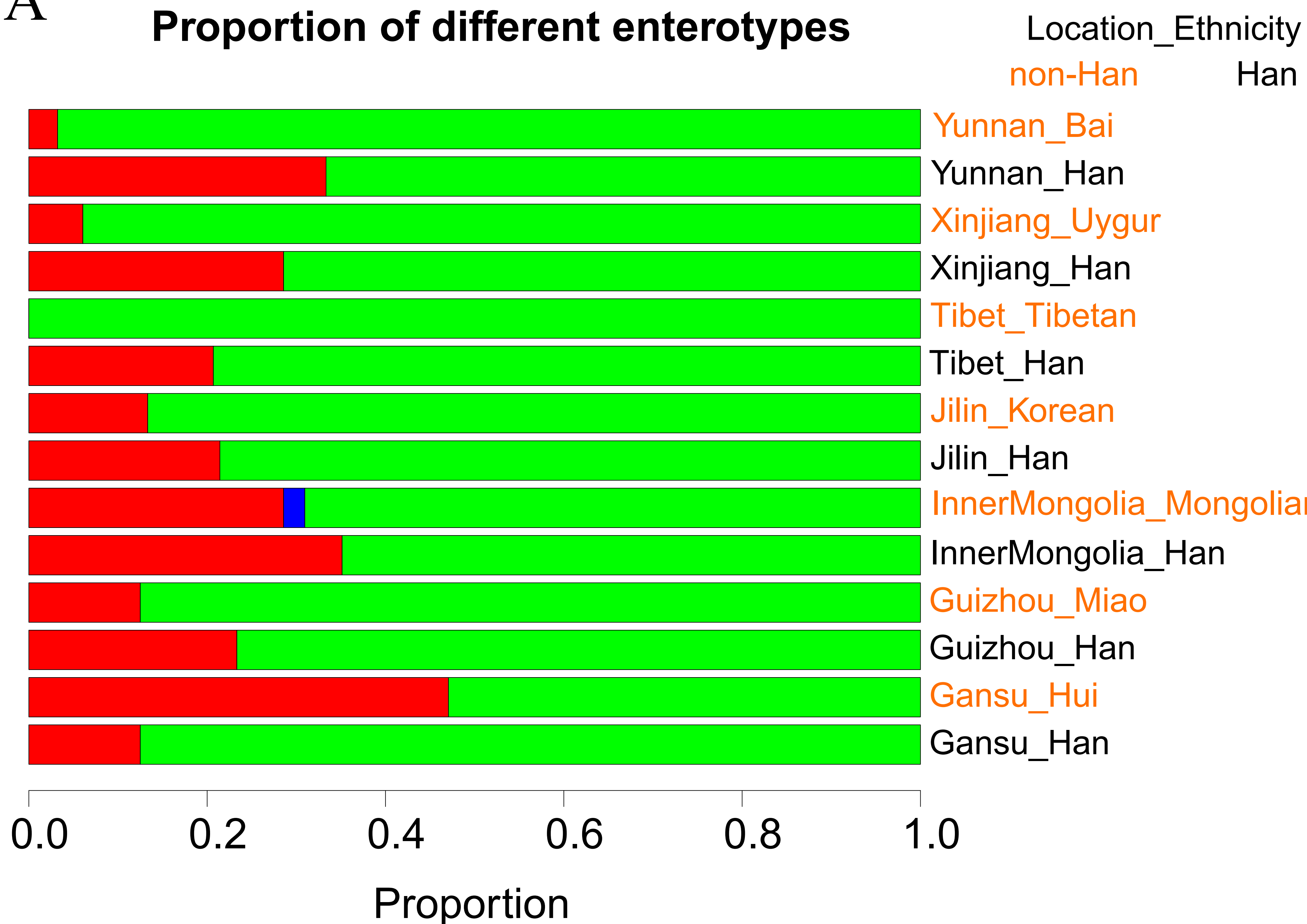

## Frequency of different enterotypes

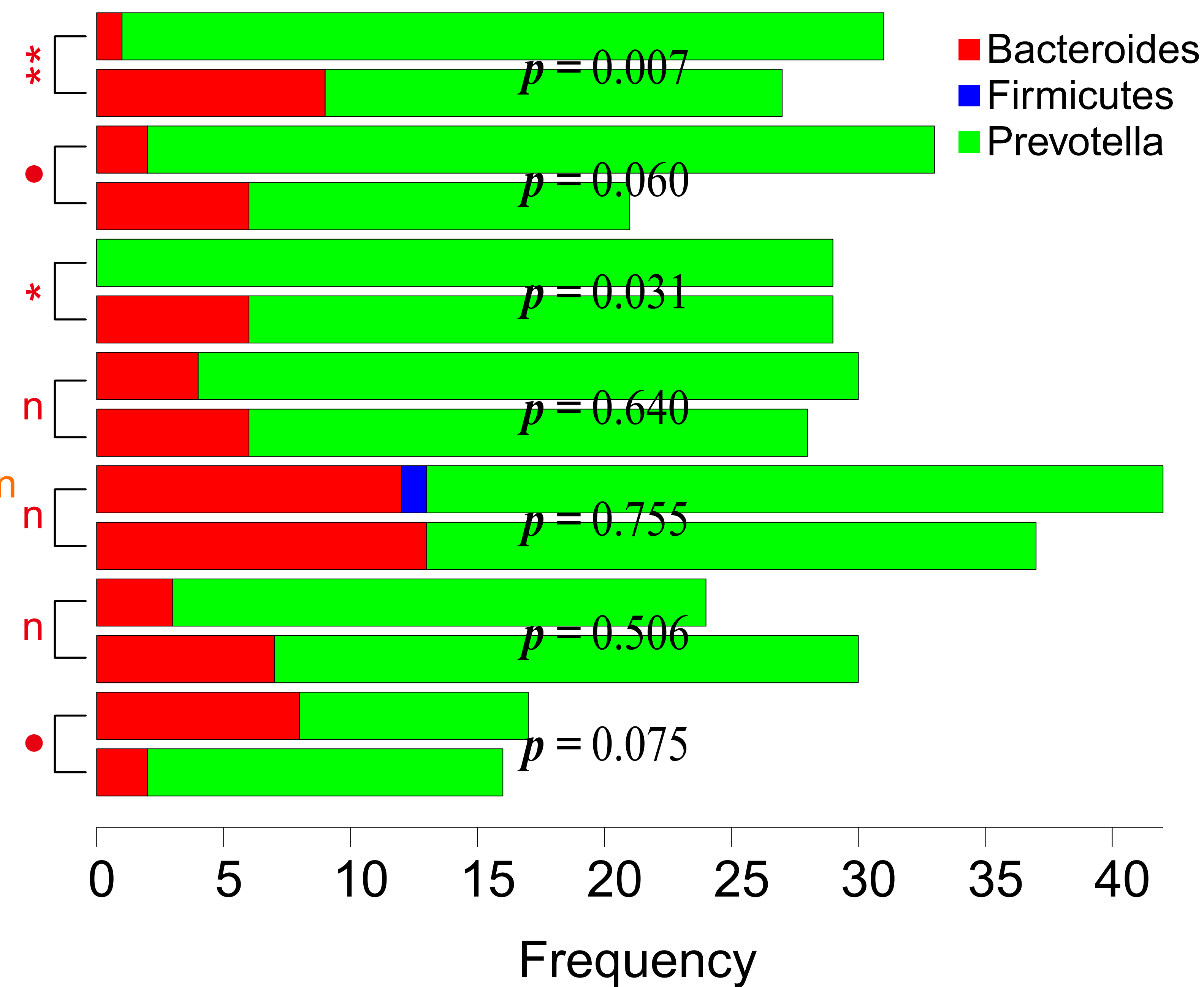

B

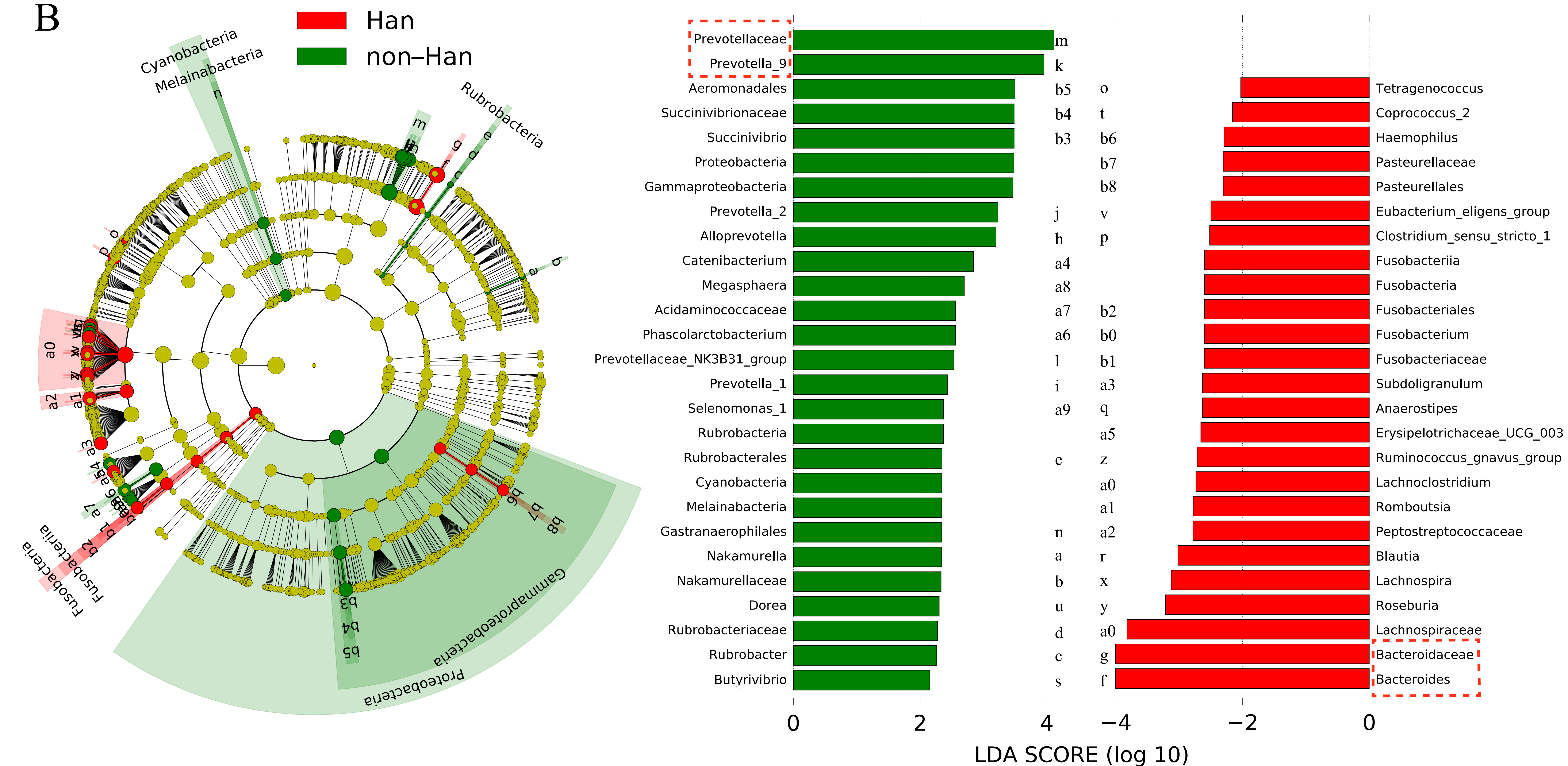

Supplement: Supplementary file 1 [file microorganisms-08-01579-s001.zip › major figures/Figure 3.pdf]
